# Supplementary material for: Contraceptive-induced menstrual changes in low- and middle-income countries: a systematic scoping review
Source: Commun Med (Lond). 2025 Dec 17;6:43. doi: 10.1038/s43856-025-01297-5 (PMC12820136; doi:10.1038/s43856-025-01297-5)
Supplement: Supplementary file 2 — Supplemental Information [file 43856_2025_1297_MOESM2_ESM.pdf]

Supplementary material - Contraceptive-induced menstrual changes in low- and middle-income countries: a systematic scoping review

Table of Contents

Supplementary Table S1A. MEDLINE Database search 3

Supplementary Table S1B. Embase Database search 6

Supplementary Table S1C. Emcare Database search 9

Supplementary Table S1D. Scopus Database search 12

Supplementary Table S1E. PsycInfo Database search 14

Supplementary Table S1F. Global Health Database search 16

Supplementary Table S1G. Global Index Medicus Database search 18

Supplementary Table S1H. Web of Science Database search 19

Supplementary Table S1I. CINAHL Database search 21

Supplementary Table S2. Distribution of studies reporting the prevalence or health impact of contraceptive-induced menstrual changes in low- and middle-income countries 26

Supplementary Table S3. Type of contraceptive used in included studies 28

Supplementary Table S4. Prevalence of contraceptive-induced menstrual changes by type of contraceptive used (220 studies) 29

Supplementary Table S5. Prevalence of contraceptive discontinuation due to menstrual changes 30

Supplementary Figure S1. Distribution of studies reporting prevalence, health impact, and treatment of CIMC by study design 33

Supplementary Figure S2. Distribution of studies reporting the prevalence or health impact of contraceptive-induced menstrual changes (a) in low- and middle-income countries (b) by 2022 World Bank country classifications by income levels 35

Supplementary Figure S3. Distribution of studies reporting treatment of contraceptive-induced menstrual changes by 2022 World Bank country classifications by income levels ... 36

Supplementary Table S1A. MEDLINE Database search
Database(s): Ovid MEDLINE(R) and Epub Ahead of Print, In-Process, In-Data-Review & Other Non-Indexed Citations, Daily and Versions 1946 to December 15, 2024
Search Strategy:

#	Searches
1	Africa/ or Africa south of the Sahara/ or North Africa/ or angola/ or benin/ or Botswana/ or Burkina Faso/ or Burundi/ or Cameroon/ or Cape Verde/ or Central Africa/ or Central African Republic/ or Chad/ or Comoros/ or Congo/ or Cote dIvoire/ or Democratic Republic Congo/ or Djibouti/ or Equatorial Guinea/ or Eritrea/ or Eswatini/ or Ethiopia/ or Gabon/ or Gambia/ or Ghana/ or Guinea/ or Guinea-Bissau/ or Kenya/ or Lesotho/ or Liberia/ or Madagascar/ or Malawi/ or Mali/ or Mozambique/ or Namibia/ or Niger/ or Nigeria/ or Rwanda/ or Sahel/ or Senegal/ or Sierra Leone/ or Somalia/ or South Africa/ or South Sudan/ or Sudan/ or Tanzania/ or Togo/ or Uganda/ or Zambia/ or Zimbabwe/ or Algeria/ or Egypt/ or Libyan Arab Jamahiriya/ or Mauritania/ or Morocco/ or Tunisia/ or Western Sahara/ or Central Africa/ or North Africa/ or African Caribbean/ or Caribbean/ or Central America/ or "South and Central America"/ or Belize/ or Costa Rica/ or El Salvador/ or Guatemala/ or Honduras/ or Nicaragua/ or Panama/ or Antillean/ or Caribbean Islands/ or Cuba/ or Dominica/ or Dominican Republic/ or Grenada/ or Guadeloupe/ or Jamaica/ or Haiti/ or Martinique/ or Saint Lucia/ or "Saint Vincent and the Grenadines"/ or "caribbean (person)"/ or Cuban/ or "dominican (dominica)"/ or "dominican (dominican republic)"/ or Haitian/ or Jamaican/ or South America/ or Argentina/ or Bolivia/ or Brazil/ or Colombia/ or Ecuador/ or French Guiana/ or Guyana/ or Paraguay/ or Peru/ or Suriname/ or Venezuela/ or Mexico/ or Asia/ or central Asia/ or Far East/ or Middle East/ or northern Asia/ or South Asia/ or western Asia/ or Kazakhstan/ or Kyrgyzstan/ or Tajikistan/ or Turkmenistan/ or Uzbekistan/ or China/ or Korea/ or Mongolia/ or Philippines/ or Southeast Asia/ or North Korea/ or Borneo/ or Cambodia/ or Indonesia/ or Laos/ or Malaysia/ or Myanmar/ or Papua New Guinea/ or Singapore/ or Thailand/ or Timor-Leste/ or Viet Nam/ or Iran/ or Iraq/ or Jordan/ or Lebanon/ or Palestine/ or Syrian Arab Republic/ or "turkey (republic)"/ or Yemen/ or Afghanistan/ or Bangladesh/ or Bhutan/ or India/ or Nepal/ or Pakistan/ or Sri Lanka/ or Armenia/ or Azerbaijan/ or "georgia (republic)"/ or "Sao Tome and Principe"/ or Mauritius/ or Pacific Islands/ or Federated States of Micronesia/ or Fiji/ or Kiribati/ or Marshall Islands/ or Melanesia/ or Nauru/ or Palau/ or Polynesia/ or Samoan Islands/ or Solomon Islands/ or Timor-Leste/ or Tonga/ or Tuvalu/ or Vanuatu/ or American Samoa/ or Samoa/ or Romania/ or Russian Federation/ or USSR/ or Croatia/ or Albania/ or Belarus/ or "Bosnia and Herzegovina"/ or Bulgaria/ or Kosovo/ or Moldova/ or "Montenegro (republic)"/ or Republic of North Macedonia/ or Serbia/ or Ukraine/ or "Federation of Bosnia and Herzegovina"/
2	developing country/ or low income country/ or middle income country/
3	(Afghanistan* or Albania* or Algeria* or Angola* or Argentina* or Armenia* or Azerbaijan* or Bangladesh* or Beliz* or Benin* or Bhutan* or Bolivia* or Bosnia* or Herzegovin* or Botswan* or Brazil* or Bulgaria* or Burkina* or Burundi* or Cabo Verde* or Cape Verde* or Cambodia* or Cameroon* or Chad* or China or Chinese or Colombia* or Comor* or Congo* or Costa Rica* or Cote d'Ivoir* or Ivory Coast or Cuba* or Djibouti* or Dominica* or Ecuador* or Egypt* or El Salvador* or Eritrea* or Ethiopia* or Fiji* or Gabon* or Gambia* or Georgia* or Ghana* or Grenad* or Guatemala* or Guinea* or Guyan* or Haiti* or Hondura* or Hungar* or India* or Indonesia* or Iran* or Iraq* or Jamaica* or Jordan* or Kazakhstan* or Kenya* or Kiribati* or Korea* or Kosov* or Kyrgyz* or Lao* or Leban* or Lesotho* or Liberia* or Libya* or Macedonia* or Madagascar* or Malawi* or Malaysia* or Maldiv* or Mali* or Marshall Island* or Mauritania* or Mauriti* or Mexic* or Moldova* or Mongolia* or Montenegr* or Morocc* or Mozambi* or Myanma* or Burmese or Namibia* or Nepal* or Nicaragua* or Niger* or Pakistan* or Palau* or Panama* or Papua New Guinea* or Paraguay* or Peru* or Philippines or Filipino or Romania* or Rwanda* or Samoa* or Sao Tome* or Senegal* or Serbia* or Seychell* or Sierra Leon* or Solomon Island* or Somalia* or Sudan* or Sri Lanka* or St Lucia* or Saint Lucia or St Vincent or Saint Vincent or Grenadines or Surinam* or Swazi* or Syria* or Tajikistan* or Tanzania* or Thai* or Timor* or Togo* or Tonga* or Tunisia* or Turk* or Tuvalu* or Uganda* or Ukrain* or Uzbekistan* or Vanuatu* or Venezuela* or Vietnam* or Viet-Nam* or West Bank or Gaza or Yemen* or Zambia* or Zimbabwe* or Russia* or Croatia* or Nauru* or Yugoslavia* or USSR or Soviet* or Byelarus* or Belarus*).mp.
4	(africa* or asia* or caribbean or central america* or latin america* or south america* or melanesia* or micronesia* or polynesia*).mp.
5	(resource-limit* or resource-poor or low-resource* or limited-resource* or resource-constrain* or constrain*-resource* or under-resource* or poor*-resource* or resource-scarce* or scarce*-resource* or low-income or middle-income or lowincome or middleincome or (low* adj3 middle-income)).mp.

6	((developing or underdeveloped or under-developed or emerging or less-developed or least-developed or less-economically developed or least-economically developed or less-affluent or least-affluent or least-industriali#ed or non-industriali#ed or deprived or poor) adj (country or countries or nation? or region? or economy or economies)).mp.
7	((developing or underdeveloped or under-developed or less-developed or least-developed) adj (population* or world)).mp.
8	(third-world* or thirdworld* or 3rd-world* or lmic or lmics or lami countr* or lalmi countr* or transitional countr*).mp.
9	(low* adj (gdp or gnp or gross domestic or gross national)).mp.
10	((underserved or under-served) adj (countr* or nation? or population*)).mp.
11	1 or 2 or 3 or 4 or 5 or 6 or 7 or 8 or 9 or 10
12	contraception/ or birth control/ or hormonal contraception/ or long-acting reversible contraception/ or oral contraception/ or ovulation inhibition/
13	contraceptive behavior/ or "oral contraceptive use"/
14	contraceptive agent/ or exp hormonal contraceptive agent/ or exp injectable contraceptive agent/ or menstruation inducing agent/ or exp oral contraceptive agent/
15	female contraceptive device/ or birth control implant/ or contraceptive patch/ or intrauterine contraceptive device/
16	female contraceptive device/ or exp birth control implant/ or contraceptive patch/ or intrauterine contraceptive device/
17	(contracepti* or anticonceptive or antifertility or antiovulatory or anti-conceptive* or anti-fertility or anti-ovulatory).mp.
18	((intrauterine or intracervical or intra-uterine or intra-cervical) adj (device* or coil*)).mp.
19	((hormonal or copper or copper releasing) adj (IUD or IUDs)).mp.
20	(LNG-IUD or levonorgestrel IUD* or levonorgestrel releasing intrauterine).mp.
21	(anovulatory agent* or anovulatory drug* or ovulation inhibitor* or ovulation block* or ovulat* inhibiting hormone* or ovulat* suppression).mp.
22	12 or 13 or 14 or 15 or 16 or 17 or 18 or 19 or 20 or 21
23	menstrual cycle/ or menstruation/ or uterine bleeding pattern/ or menstrual cycle length/
24	"amenorrhea and oligomenorrhea"/ or menstruation disorder/ or amenorrhea/ or menstrual irregularity/ or oligomenorrhea/ or dysmenorrhea/ or "menorrhagia and metrorrhagia"/ or menstrual related disorder/ or menorrhagia/ or metrorrhagia/ or spotting/ or menometrorrhagia/
25	(menstrua* or menses or catamenia or menstruum or eumeno?rh?ea* or meno?rh?ea*).mp.
26	(ameno?rh?ea* or amenor*ea or amenia or hypomeno?rh?ea* or hypomenor*ea or oligomeno?rh?oea* or oligomenor*ea or spaniomeno?rh?ea* or spaniomenor*ea or spanomeno?rh?ea* or spanomenor*ea or meno?rhagi* or menorr?agi* or menor*agi* or hypermeno?rhea* or hypermenorr?oea* or hypermeno?rh?ea* or meno?rhagy or menorr?agy or polymenor?hea* or polymenorr?ea or polymeno?rh?ea or menometro?rhagi* or menometrorr?agi* or menometror*agi? or metro?rhag* or metror*agy or metro?rh?ea* or metror*ea).mp.
27	(dysfunctional uterine bleeding or dysfunctional uterus bleeding or intermenstrual bleeding or intermenstrual h?emo?r?age* or intermenstrual h?em?or*age).mp.
28	((bleed* or bled or blood*) and monthly period?).mp.
29	((heavy or light) adj period?).mp.
30	((change* or irregular* or disturb* or disorder* or pattern* or cycle or cycle length) and monthly period?).mp.
31	((suffer* or disabl* or debilitat* or burden* or impact*) adj3 (((change* or irregular* or disturb* or disorder* or pattern* or cycle or cycle length) and menstrual) or monthly period?)).mp. [mp=title, book title, abstract, original title, name of substance word, subject heading word, floating sub-heading word, keyword heading word, organism supplementary concept word, protocol supplementary concept word, rare disease supplementary concept word, unique identifier, synonyms, population supplementary concept word, anatomy supplementary concept word]
32	23 or 24 or 25 or 26 or 27 or 28 or 29 or 30 or 31
33	exp nonsteroid antiinflammatory agent/ or exp prostaglandin synthase inhibitor/ or exp cyclooxygenase 2 inhibitor/

34	(non-steroid* antiinflammatory or non-steroid* anti-inflammatory or nonsteroid antiinflammatory or nonsteroid* anti-inflammatory or NSAID or NSAIDs).mp.
35	(cyclooxygenase inhibitor* or cyclo-oxygenase inhibitor* or prostaglandin synthetase inhibitor* or cyclooxygenase-2 inhibitor* or COX-2 inhibitor* or COX-2 specific inhibitor* or COX2 inhibitor* or COX2 specific inhibitor* or coxib or coxibs).mp.
36	tranexamic acid/
37	mifepristone.mp.
38	(antifibrinolytic* or fibrinolysis inhibitor* or aminomethyl cyclohexane carboxylic acid* or aminomethyl cyclohexanecarboxylic acid* or aminomethylcyclohexane carbonic acid* or aminomethylcyclohexane carboxylic acid* or aminomethylcyclohexanecarbonic acid* or aminomethylcyclohexanecarboxylic acid* or aminomethylcyclohexanocarboxylic acid* or aminomethylcyclohexanoic acid* or tranexamic acid* or tranexam or tranexanic acid* or transexamic acid* or traxamic or TXA).mp.
39	33 or 34 or 35 or 36 or 37 or 38
40	11 and 22 and 32
41	22 and 32 and 39
42	exp animals/ not humans.sh.
43	40 or 41
44	43 not 42
45	limit 44 to yr="2000 -Current"
46	limit 45 to (comment or editorial or letter)
47	45 not 46

Supplementary Table S1B. Embase Database search
Database(s): **Embase Classic+Embase** 1947 to 2024 December 15
Search Strategy:

#	Searches
1	Africa/ or Africa south of the Sahara/ or North Africa/ or angola/ or benin/ or Botswana/ or Burkina Faso/ or Burundi/ or Cameroon/ or Cape Verde/ or Central Africa/ or Central African Republic/ or Chad/ or Comoros/ or Congo/ or Cote dIvoire/ or Democratic Republic Congo/ or Djibouti/ or Equatorial Guinea/ or Eritrea/ or Eswatini/ or Ethiopia/ or Gabon/ or Gambia/ or Ghana/ or Guinea/ or Guinea-Bissau/ or Kenya/ or Lesotho/ or Liberia/ or Madagascar/ or Malawi/ or Mali/ or Mozambique/ or Namibia/ or Niger/ or Nigeria/ or Rwanda/ or Sahel/ or Senegal/ or Sierra Leone/ or Somalia/ or South Africa/ or South Sudan/ or Sudan/ or Tanzania/ or Togo/ or Uganda/ or Zambia/ or Zimbabwe/ or Algeria/ or Egypt/ or Libyan Arab Jamahiriya/ or Mauritania/ or Morocco/ or Tunisia/ or Western Sahara/ or Central Africa/ or North Africa/ or African Caribbean/ or Caribbean/ or Central America/ or "South and Central America"/ or Belize/ or Costa Rica/ or El Salvador/ or Guatemala/ or Honduras/ or Nicaragua/ or Panama/ or Antillean/ or Caribbean Islands/ or Cuba/ or Dominica/ or Dominican Republic/ or Grenada/ or Guadeloupe/ or Jamaica/ or Haiti/ or Martinique/ or Saint Lucia/ or "Saint Vincent and the Grenadines"/ or "caribbean (person)"/ or Cuban/ or "dominican (dominica)"/ or "dominican (dominican republic)"/ or Haitian/ or Jamaican/ or South America/ or Argentina/ or Bolivia/ or Brazil/ or Colombia/ or Ecuador/ or French Guiana/ or Guyana/ or Paraguay/ or Peru/ or Suriname/ or Venezuela/ or Mexico/ or Asia/ or central Asia/ or Far East/ or Middle East/ or northern Asia/ or South Asia/ or western Asia/ or Kazakhstan/ or Kyrgyzstan/ or Tajikistan/ or Turkmenistan/ or Uzbekistan/ or China/ or Korea/ or Mongolia/ or Philippines/ or Southeast Asia/ or North Korea/ or Borneo/ or Cambodia/ or Indonesia/ or Laos/ or Malaysia/ or Myanmar/ or Papua New Guinea/ or Singapore/ or Thailand/ or Timor-Leste/ or Viet Nam/ or Iran/ or Iraq/ or Jordan/ or Lebanon/ or Palestine/ or Syrian Arab Republic/ or "turkey (republic)"/ or Yemen/ or Afghanistan/ or Bangladesh/ or Bhutan/ or India/ or Nepal/ or Pakistan/ or Sri Lanka/ or Armenia/ or Azerbaijan/ or "georgia (republic)"/ or "Sao Tome and Principe"/ or Mauritius/ or Pacific Islands/ or Federated States of Micronesia/ or Fiji/ or Kiribati/ or Marshall Islands/ or Melanesia/ or Nauru/ or Palau/ or Polynesia/ or Samoan Islands/ or Solomon Islands/ or Timor-Leste/ or Tonga/ or Tuvalu/ or Vanuatu/ or American Samoa/ or Samoa/ or Romania/ or Russian Federation/ or USSR/ or Croatia/ or Albania/ or Belarus/ or "Bosnia and Herzegovina"/ or Bulgaria/ or Kosovo/ or Moldova/ or "Montenegro (republic)"/ or Republic of North Macedonia/ or Serbia/ or Ukraine/ or "Federation of Bosnia and Herzegovina"/
2	developing country/ or low income country/ or middle income country/
3	(Afghanistan* or Albania* or Algeria* or Angola* or Argentina* or Armenia* or Azerbaijan* or Bangladesh* or Beliz* or Benin* or Bhutan* or Bolivia* or Bosnia* or Herzegovin* or Botswan* or Brazil* or Bulgaria* or Burkina* or Burundi* or Cabo Verde* or Cape Verde* or Cambodia* or Cameroon* or Chad* or China or Chinese or Colombia* or Comor* or Congo* or Costa Rica* or Cote d'Ivoir* or Ivory Coast or Cuba* or Djibouti* or Dominica* or Ecuador* or Egypt* or El Salvador* or Eritrea* or Ethiopia* or Fiji* or Gabon* or Gambia* or Georgia* or Ghana* or Grenad* or Guatemala* or Guinea* or Guyan* or Haiti* or Hondura* or Hungar* or India* or Indonesia* or Iran* or Iraq* or Jamaica* or Jordan* or Kazakhstan* or Kenya* or Kiribati* or Korea* or Kosov* or Kyrgyz* or Lao* or Leban* or Lesotho* or Liberia* or Libya* or Macedonia* or Madagascar* or Malawi* or Malaysia* or Maldiv* or Mali* or Marshall Island* or Mauritania* or Mauriti* or Mexic* or Moldova* or Mongolia* or Montenegr* or Morocc* or Mozambi* or Myanma* or Burmese or Namibia* or Nepal* or Nicaragua* or Niger* or Pakistan* or Palau* or Panama* or Papua New Guinea* or Paraguay* or Peru* or Philippines or Filipino or Romania* or Rwanda* or Samoa* or Sao Tome* or Senegal* or Serbia* or Seychell* or Sierra Leon* or Solomon Island* or Somalia* or Sudan* or Sri Lanka* or St Lucia* or Saint Lucia or St Vincent or Saint Vincent or Grenadines or Surinam* or Swazi* or Syria* or Tajikistan* or Tanzania* or Thai* or Timor* or Togo* or Tonga* or Tunisia* or Turk* or Tuvalu* or Uganda* or Ukrain* or Uzbekistan* or Vanuatu* or Venezuela* or Vietnam* or Viet-Nam* or West Bank or Gaza or Yemen* or Zambia* or Zimbabwe* or Russia* or Croatia* or Nauru* or Yugoslavia* or USSR or Soviet* or Byelarus* or Belarus*).mp.
4	(africa* or asia* or caribbean or central america* or latin america* or south america* or melanesia* or micronesia* or polynesia*).mp.
5	(resource-limit* or resource-poor or low-resource* or limited-resource* or resource-constrain* or constrain*-resource* or under-resource* or poor*-resource* or resource-scarce* or scarce*-resource* or low-income or middle-income or lowincome or middleincome or (low* adj3 middle-income)).mp.
6	((developing or underdeveloped or under-developed or emerging or less-developed or least-developed or less-economically developed or least-economically developed or less-affluent or least-affluent or least-

	industriali#ed or non-industriali#ed or deprived or poor) adj (country or countries or nation? or region? or economy or economies)).mp.
7	((developing or underdeveloped or under-developed or less-developed or least-developed) adj (population* or world)).mp.
8	(third-world* or thirdworld* or 3rd-world* or lmic or lmics or lami countr* or lalmi countr* or transitional countr*).mp.
9	(low* adj (gdp or gnp or gross domestic or gross national)).mp.
10	((underserved or under-served) adj (countr* or nation? or population*)).mp.
11	1 or 2 or 3 or 4 or 5 or 6 or 7 or 8 or 9 or 10
12	contraception/ or birth control/ or hormonal contraception/ or long-acting reversible contraception/ or oral contraception/ or ovulation inhibition/
13	contraceptive behavior/ or "oral contraceptive use"/
14	contraceptive agent/ or exp hormonal contraceptive agent/ or exp injectable contraceptive agent/ or menstruation inducing agent/ or exp oral contraceptive agent/
15	female contraceptive device/ or birth control implant/ or contraceptive patch/ or intrauterine contraceptive device/
16	female contraceptive device/ or exp birth control implant/ or contraceptive patch/ or intrauterine contraceptive device/
17	etonogestrel/
18	(contracepti* or anticonceptive or antifertility or antiovulatory or anti-conceptive* or anti-fertility or anti-ovulatory).mp.
19	((intrauterine or intracervical or intra-uterine or intra-cervical) adj (device* or coil*)).mp.
20	((hormonal or copper or copper releasing) adj (IUD or IUDs)).mp.
21	(LNG-IUD or levonorgestrel IUD* or levonorgestrel releasing intrauterine).mp.
22	(anovulatory agent* or anovulatory drug* or ovulation inhibitor* or ovulation block* or ovulat* inhibiting hormone* or ovulat* suppression).mp.
23	12 or 13 or 14 or 15 or 16 or 17 or 18 or 19 or 20 or 21 or 22
24	menstrual cycle/ or menstruation/ or uterine bleeding pattern/ or menstrual cycle length/
25	"amenorrhea and oligomenorrhea"/ or menstruation disorder/ or amenorrhea/ or menstrual irregularity/ or oligomenorrhea/ or dysmenorrhea/ or "menorrhagia and metrorrhagia"/ or menstrual related disorder/ or menorrhagia/ or metrorrhagia/ or spotting/ or menometrorrhagia/
26	uterus bleeding/
27	(menstrua* or menses or catamenia or menstruum or eumeno?rh?ea* or meno?rh?ea*).mp.
28	(ameno?rh?ea* or amenor*ea or amenia or hypomeno?rh?ea* or hypomenor*ea or oligomeno?rh?oea* or oligomenor*ea or spaniomeno?rh?ea* or spaniomenor*ea or spanomeno?rh?ea* or spanomenor*ea or meno?rhagi* or menorr?agi* or menor*agi* or hypermeno?rhea* or hypermenorr?oea* or hypermeno?rh?ea* or meno?rhagy or menorr?agy or polymenor?hea* or polymenorr?ea or polymeno?rh?ea or menometro?rhagi* or menometrorr?agi* or menometror*agi? or metro?rhag* or metror*agy or metro?rh?ea* or metror*ea).mp.
29	(dysfunctional uterine bleeding or dysfunctional uterus bleeding or intermenstrual bleeding or intermenstrual h?emo?r?age* or intermenstrual h?em?or*age).mp.
30	((bleed* or bled or blood*) and monthly period?).mp.
31	((heavy or light) adj period?).mp.
32	((change* or irregular* or disturb* or disorder* or pattern* or cycle or cycle length) and monthly period?).mp.
33	((suffer* or disabl* or debilitat* or burden* or impact*) adj3 (((change* or irregular* or disturb* or disorder* or pattern* or cycle or cycle length) and menstrual) or monthly period?)).mp.
34	24 or 25 or 26 or 27 or 28 or 29 or 30 or 31 or 32 or 33
35	exp nonsteroid antiinflammatory agent/ or exp prostaglandin synthase inhibitor/ or exp cyclooxygenase 2 inhibitor/
36	(non-steroid* antiinflammatory or non-steroid* anti-inflammatory or nonsteroid antiinflammatory or nonsteroid* anti-inflammatory or NSAID or NSAIDs).mp.

37	(cyclooxygenase inhibitor* or cyclo-oxygenase inhibitor* or prostaglandin synthetase inhibitor* or cyclooxygenase-2 inhibitor* or COX-2 inhibitor* or COX-2 specific inhibitor* or COX2 inhibitor* or COX2 specific inhibitor* or coxib or coxibs).mp.
38	tranexamic acid/
39	mifepristone.mp.
40	(antifibrinolytic* or fibrinolysis inhibitor* or aminomethyl cyclohexane carboxylic acid* or aminomethyl cyclohexanecarboxylic acid* or aminomethylcyclohexane carbonic acid* or aminomethylcyclohexane carboxylic acid* or aminomethylcyclohexanecarbonic acid* or aminomethylcyclohexanecarboxylic acid* or aminomethylcyclohexanocarboxylic acid* or aminomethylcyclohexanoic acid* or tranexamic acid* or tranexam or tranexanic acid* or transexamic acid* or traxamic or TXA).mp.
41	35 or 36 or 37 or 38 or 39 or 40
42	11 and 23 and 34
43	23 and 34 and 41
44	(exp animal/ or nonhuman/ or exp invertebrate/ or animal.hw.) not exp human/
45	42 or 43
46	45 not 44
47	limit 46 to yr="2000 -Current"
48	limit 47 to (conference abstract or editorial or letter)
49	47 not 48

Supplementary Table S1C. Emcare Database search
Database(s): **Ovid Emcare** 1995 to 2024 Week 49
Search Strategy:

#	Searches
1	Africa/ or Africa south of the Sahara/ or North Africa/ or angola/ or benin/ or Botswana/ or Burkina Faso/ or Burundi/ or Cameroon/ or Cape Verde/ or Central Africa/ or Central African Republic/ or Chad/ or Comoros/ or Congo/ or Cote dIvoire/ or Democratic Republic Congo/ or Djibouti/ or Equatorial Guinea/ or Eritrea/ or Eswatini/ or Ethiopia/ or Gabon/ or Gambia/ or Ghana/ or Guinea/ or Guinea-Bissau/ or Kenya/ or Lesotho/ or Liberia/ or Madagascar/ or Malawi/ or Mali/ or Mozambique/ or Namibia/ or Niger/ or Nigeria/ or Rwanda/ or Sahel/ or Senegal/ or Sierra Leone/ or Somalia/ or South Africa/ or South Sudan/ or Sudan/ or Tanzania/ or Togo/ or Uganda/ or Zambia/ or Zimbabwe/ or Algeria/ or Egypt/ or Libyan Arab Jamahiriya/ or Mauritania/ or Morocco/ or Tunisia/ or Western Sahara/ or Central Africa/ or North Africa/ or African Caribbean/ or Caribbean/ or Central America/ or "South and Central America"/ or Belize/ or Costa Rica/ or El Salvador/ or Guatemala/ or Honduras/ or Nicaragua/ or Panama/ or Antillean/ or Caribbean Islands/ or Cuba/ or Dominica/ or Dominican Republic/ or Grenada/ or Guadeloupe/ or Jamaica/ or Haiti/ or Martinique/ or Saint Lucia/ or "Saint Vincent and the Grenadines"/ or "caribbean (person)"/ or Cuban/ or "dominican (dominica)"/ or "dominican (dominican republic)"/ or Haitian/ or Jamaican/ or South America/ or Argentina/ or Bolivia/ or Brazil/ or Colombia/ or Ecuador/ or French Guiana/ or Guyana/ or Paraguay/ or Peru/ or Suriname/ or Venezuela/ or Mexico/ or Asia/ or central Asia/ or Far East/ or Middle East/ or northern Asia/ or South Asia/ or western Asia/ or Kazakhstan/ or Kyrgyzstan/ or Tajikistan/ or Turkmenistan/ or Uzbekistan/ or China/ or Korea/ or Mongolia/ or Philippines/ or Southeast Asia/ or North Korea/ or Borneo/ or Cambodia/ or Indonesia/ or Laos/ or Malaysia/ or Myanmar/ or Papua New Guinea/ or Singapore/ or Thailand/ or Timor-Leste/ or Viet Nam/ or Iran/ or Iraq/ or Jordan/ or Lebanon/ or Palestine/ or Syrian Arab Republic/ or "turkey (republic)"/ or Yemen/ or Afghanistan/ or Bangladesh/ or Bhutan/ or India/ or Nepal/ or Pakistan/ or Sri Lanka/ or Armenia/ or Azerbaijan/ or "georgia (republic)"/ or "Sao Tome and Principe"/ or Mauritius/ or Pacific Islands/ or Federated States of Micronesia/ or Fiji/ or Kiribati/ or Marshall Islands/ or Melanesia/ or Nauru/ or Palau/ or Polynesia/ or Samoan Islands/ or Solomon Islands/ or Timor-Leste/ or Tonga/ or Tuvalu/ or Vanuatu/ or American Samoa/ or Samoa/ or Romania/ or Russian Federation/ or USSR/ or Croatia/ or Albania/ or Belarus/ or "Bosnia and Herzegovina"/ or Bulgaria/ or Kosovo/ or Moldova/ or "Montenegro (republic)"/ or Republic of North Macedonia/ or Serbia/ or Ukraine/ or "Federation of Bosnia and Herzegovina"/
2	developing country/ or low income country/ or middle income country/
3	(Afghanistan* or Albania* or Algeria* or Angola* or Argentina* or Armenia* or Azerbaijan* or Bangladesh* or Beliz* or Benin* or Bhutan* or Bolivia* or Bosnia* or Herzegovin* or Botswan* or Brazil* or Bulgaria* or Burkina* or Burundi* or Cabo Verde* or Cape Verde* or Cambodia* or Cameroon* or Chad* or China or Chinese or Colombia* or Comor* or Congo* or Costa Rica* or Cote d'Ivoir* or Ivory Coast or Cuba* or Djibouti* or Dominica* or Ecuador* or Egypt* or El Salvador* or Eritrea* or Ethiopia* or Fiji* or Gabon* or Gambia* or Georgia* or Ghana* or Grenad* or Guatemala* or Guinea* or Guyan* or Haiti* or Hondura* or Hungar* or India* or Indonesia* or Iran* or Iraq* or Jamaica* or Jordan* or Kazakhstan* or Kenya* or Kiribati* or Korea* or Kosov* or Kyrgyz* or Lao* or Leban* or Lesotho* or Liberia* or Libya* or Macedonia* or Madagascar* or Malawi* or Malaysia* or Maldiv* or Mali* or Marshall Island* or Mauritania* or Mauriti* or Mexic* or Moldova* or Mongolia* or Montenegr* or Morocc* or Mozambi* or Myanma* or Burmese or Namibia* or Nepal* or Nicaragua* or Niger* or Pakistan* or Palau* or Panama* or Papua New Guinea* or Paraguay* or Peru* or Philippines or Filipino or Romania* or Rwanda* or Samoa* or Sao Tome* or Senegal* or Serbia* or Seychell* or Sierra Leon* or Solomon Island* or Somalia* or Sudan* or Sri Lanka* or St Lucia* or Saint Lucia or St Vincent or Saint Vincent or Grenadines or Surinam* or Swazi* or Syria* or Tajikistan* or Tanzania* or Thai* or Timor* or Togo* or Tonga* or Tunisia* or Turk* or Tuvalu* or Uganda* or Ukrain* or Uzbekistan* or Vanuatu* or Venezuela* or Vietnam* or Viet-Nam* or West Bank or Gaza or Yemen* or Zambia* or Zimbabwe* or Russia* or Croatia* or Nauru* or Yugoslavia* or USSR or Soviet* or Byelarus* or Belarus*).mp.
4	(africa* or asia* or caribbean or central america* or latin america* or south america* or melanesia* or micronesia* or polynesia*).mp.
5	(resource-limit* or resource-poor or low-resource* or limited-resource* or resource-constrain* or constrain*-resource* or under-resource* or poor*-resource* or resource-scarce* or scarce*-resource* or low-income or middle-income or lowincome or middleincome or (low* adj3 middle-income)).mp.

6	((developing or underdeveloped or under-developed or emerging or less-developed or least-developed or less-economically developed or least-economically developed or less-affluent or least-affluent or least-industriali#ed or non-industriali#ed or deprived or poor) adj (country or countries or nation? or region? or economy or economies)).mp.
7	((developing or underdeveloped or under-developed or less-developed or least-developed) adj (population* or world)).mp.
8	(third-world* or thirdworld* or 3rd-world* or lmic or lmics or lami countr* or lalmi countr* or transitional countr*).mp.
9	(low* adj (gdp or gnp or gross domestic or gross national)).mp.
10	((underserved or under-served) adj (countr* or nation? or population*)).mp.
11	1 or 2 or 3 or 4 or 5 or 6 or 7 or 8 or 9 or 10
12	contraception/ or birth control/ or hormonal contraception/ or long-acting reversible contraception/ or oral contraception/ or ovulation inhibition/
13	contraceptive behavior/ or "oral contraceptive use"/
14	contraceptive agent/ or exp hormonal contraceptive agent/ or exp injectable contraceptive agent/ or menstruation inducing agent/ or exp oral contraceptive agent/
15	female contraceptive device/ or birth control implant/ or contraceptive patch/ or intrauterine contraceptive device/
16	female contraceptive device/ or exp birth control implant/ or contraceptive patch/ or intrauterine contraceptive device/
17	etonogestrel/
18	(contracepti* or anticonceptive or antifertility or antiovulatory or anti-conceptive* or anti-fertility or anti-ovulatory).mp.
19	((intrauterine or intracervical or intra-uterine or intra-cervical) adj (device* or coil*)).mp.
20	((hormonal or copper or copper releasing) adj (IUD or IUDs)).mp.
21	(LNG-IUD or levonorgestrel IUD* or levonorgestrel releasing intrauterine).mp.
22	(anovulatory agent* or anovulatory drug* or ovulation inhibitor* or ovulation block* or ovulat* inhibiting hormone* or ovulat* suppression).mp.
23	12 or 13 or 14 or 15 or 16 or 17 or 18 or 19 or 20 or 21 or 22
24	menstrual cycle/ or menstruation/ or uterine bleeding pattern/ or menstrual cycle length/
25	"amenorrhea and oligomenorrhea"/ or menstruation disorder/ or amenorrhea/ or menstrual irregularity/ or oligomenorrhea/ or dysmenorrhea/ or "menorrhagia and metrorrhagia"/ or menstrual related disorder/ or menorrhagia/ or metrorrhagia/ or spotting/ or menometrorrhagia/
26	uterus bleeding/
27	(menstrua* or menses or catamenia or menstruum or eumeno?rh?ea* or meno?rh?ea*).mp.
28	(ameno?rh?ea* or amenor*ea or amenia or hypomeno?rh?ea* or hypomenor*ea or oligomeno?rh?oea* or oligomenor*ea or spaniomeno?rh?ea* or spaniomenor*ea or spanomeno?rh?ea* or spanomenor*ea or meno?rhagi* or menorr?agi* or menor*agi* or hypermeno?rhea* or hypermenorr?oea* or hypermeno?rh?ea* or meno?rhagy or menorr?agy or polymenor?hea* or polymenorr?ea or polymeno?rh?ea or menometro?rhagi* or menometrorr?agi* or menometror*agi? or metro?rhag* or metror*agy or metro?rh?ea* or metror*ea).mp.
29	(dysfunctional uterine bleeding or dysfunctional uterus bleeding or intermenstrual bleeding or intermenstrual h?emo?r?age* or intermenstrual h?em?or*age).mp.
30	((bleed* or bled or blood*) and monthly period?).mp.
31	((heavy or light) adj period?).mp.
32	((change* or irregular* or disturb* or disorder* or pattern* or cycle or cycle length) and monthly period?).mp.
33	((suffer* or disabl* or debilitat* or burden* or impact*) adj3 (((change* or irregular* or disturb* or disorder* or pattern* or cycle or cycle length) and menstrual) or monthly period?)).mp.
34	24 or 25 or 26 or 27 or 28 or 29 or 30 or 31 or 32 or 33
35	exp nonsteroid antiinflammatory agent/ or exp prostaglandin synthase inhibitor/ or exp cyclooxygenase 2 inhibitor/
36	(non-steroid* antiinflammatory or non-steroid* anti-inflammatory or nonsteroid antiinflammatory or nonsteroid* anti-inflammatory or NSAID or NSAIDs).mp.

37	(cyclooxygenase inhibitor* or cyclo-oxygenase inhibitor* or prostaglandin synthetase inhibitor* or cyclooxygenase-2 inhibitor* or COX-2 inhibitor* or COX-2 specific inhibitor* or COX2 inhibitor* or COX2 specific inhibitor* or coxib or coxibs).mp.
38	tranexamic acid/
39	mifepristone.mp.
40	(antifibrinolytic* or fibrinolysis inhibitor* or aminomethyl cyclohexane carboxylic acid* or aminomethyl cyclohexanecarboxylic acid* or aminomethylcyclohexane carbonic acid* or aminomethylcyclohexane carboxylic acid* or aminomethylcyclohexanecarbonic acid* or aminomethylcyclohexanecarboxylic acid* or aminomethylcyclohexanocarboxylic acid* or aminomethylcyclohexanoic acid* or tranexamic acid* or tranexam or tranexanic acid* or transexamic acid* or traxamic or TXA).mp.
41	35 or 36 or 37 or 38 or 39 or 40
42	11 and 23 and 34
43	23 and 34 and 41
44	(exp animal/ or nonhuman/ or exp invertebrate/ or animal.hw.) not exp human/
45	42 or 43
46	45 not 44
47	limit 46 to yr="2000 -Current"
48	limit 47 to (conference abstract or editorial or letter)
49	47 not 48

Supplementary Table S1D. Scopus Database search

((TITLE-ABS-KEY ("menstrual cycle" OR menstruation OR "uterine bleeding pattern" OR "menstrual cycle length" OR amenorrh?ea OR "menstrual related disorder" OR oligomenorrh?ea OR "menstruation disorder" OR "menstural irregular*" OR "menstrual change*" OR (("contraceptive associated" OR "contraceptive induced") W/2 ("menstrual change*" OR "menstrual irregular*")) OR "dysfunctional uterine bleeding" OR "dysfunctional uterus bleeding" OR "intermenstrual bleeding" OR "intermenstrual h?emo?r?age*" OR "intermenstrual h?em?or*age" OR ((change* OR irregular* OR disturb* OR disorder* OR pattern* OR cycle OR "cycle length") AND "monthly period?") OR ((suffer* OR disabl* OR debilitat* OR burden* OR impact*) W/3 (((change* OR irregular* OR disturb* OR disorder* OR pattern* OR cycle OR "cycle length") AND menstrual) OR "monthly period?")))) AND (TITLE-ABS-KEY (contracepti* OR "birth control" OR "hormon* contracepti*" OR "oral contracepti* agent" OR "birth control implant" OR "intrauterine contracepti* device" OR iud OR iucd OR "injectable contracepti*" OR "contracepti* patch" OR ((hormonal OR copper OR "copper releasing") W/0 (iud OR iuds OR "intrauterine contracepti*")) OR lng-iud OR "levonorgestrel IUD" OR "levonorgestrel releasing intrauterine")) AND ((TITLE-ABS-KEY ((low* W/0 (gdp OR gnp OR "gross domestic" OR "gross national"))) OR TITLE-ABS-KEY (((underserved OR "under-served") W/0 (countr* OR nation? OR population*))) OR TITLE-ABS-KEY (("resource-limit*" OR "resource-poor" OR "low-resource*" OR "limited-resource*" OR "resource-constrain*" OR "constrain*-resource*" OR "under-resource*" OR "poor*-resource*" OR "resource-scarce*" OR "scarce*-resource*" OR "low-income" OR "middle-income" OR lowincome OR middleincome OR (low* W/2 "middle-income"))) OR TITLE-ABS-KEY ((("third-world*" OR thirdworld* OR "3rd-world*" OR lmic OR lmics OR "lami countr*" OR "lalmi countr*" OR "transitional countr*"))) OR TITLE-ABS-KEY (((developing OR underdeveloped OR "under-developed" OR "less-developed" OR "least-developed") W/0 (population* OR world))) OR TITLE-ABS-KEY (((developing OR underdeveloped OR "under-developed" OR emerging OR "less-developed" OR "least-developed" OR "less-economically developed" OR "least-economically developed" OR "less-affluent" OR "least-affluent" OR "least-industriali?ed" OR "non-industriali?ed" OR deprived OR poor) W/0 (country OR countries OR nation? OR region? OR economy OR economies))) OR TITLE-ABS-KEY ((afghanistan* OR albania* OR algeria* OR angola* OR argentina* OR armenia* OR azerbaijan* OR bangla desh* OR beliz* OR benin* OR bhutan* OR bolivia* OR bosnia* OR herzegovin* OR botswan* OR brazil* OR bulgaria* OR burkina* OR burundi* OR "Cabo Verde*" OR "Cape Verde*" OR cambodia* OR cameroon* OR chad* OR china OR chinese OR colombia* OR comor* OR con go* OR "Costa Rica*" OR "Cote d'Ivoir*" OR "Ivory Coast" OR cuba* OR djibouti* OR dominica* OR ecuador* OR egypt* OR "El Salvador*" OR eritrea* OR ethiopia* OR fiji* OR gabon* OR gambia* OR georgia* OR ghana* OR grenad * OR guatemala* OR guinea* OR guyan* OR haiti* OR hondura* OR hungar* OR india* OR indonesia* O R iran* OR iraq* OR jamaica* OR jordan* OR kazakhstan* OR kenya* OR kiribati* OR korea* OR kosov* OR kyrgyz* OR lao* OR leban* OR lesotho* OR liberia* OR libya* OR macedonia* OR madagascar* OR malawi* OR malaysia* OR maldiv* OR mali* OR "Marshall Island*" OR mauritania* OR mauriti* OR mexic* OR moldova* OR mongolia* OR montenegr* OR morocc * OR mozambi* OR myanma* OR burmese OR namibia* OR nepal* OR nicaragua* OR niger* OR pakistan * OR palau* OR panama* OR "Papua New Guinea*" OR paraguay* OR peru* OR philippines OR filipino OR romania* OR rwanda* OR samoa* OR "S ao Tome*" OR senegal* OR serbia* OR seychell* OR "Sierra Leon*" OR "Solomon Island*" OR somalia* OR sudan* OR "Sri Lanka*" OR "St Lucia*" OR "Saint Lucia" OR "St Vincent" OR "Saint Vincent" OR grenadines OR surinam* OR swazi* OR syria* OR tajikistan* OR tanzania* OR thai* OR timo r* OR togo* OR tonga* OR tunisia* OR turk* OR tuvalu* OR uganda* OR ukrain* OR uzbekistan* OR van uatu* OR venezuela* OR vietnam* OR "Viet-Nam*" OR "West Bank" OR gaza OR yemen* OR zambia* OR zimbabwe* OR russia* OR croatia* OR nauru* OR yugoslavia * OR ussr OR soviet* OR byelarus* OR belarus*))))) OR ((TITLE-ABS-KEY ("nonsteroid* anti-inflammatory" OR "non-steroid* anti-inflammatory" OR "nonsteroid anti-inflammatory" OR "nonsteroid* anti-inflammatory" OR nsaid OR nsaids OR "cyclooxygenase inhibitor*" OR "cyclo-oxygenase inhibitor*" OR "prostaglandin synthetase inhibitor*" OR "cyclooxygenase-2 inhibitor*" OR "COX-2 inhibitor*" OR "COX-2 specific inhibitor*" OR "COX2 inhibitor*" OR "COX2 specific inhibitor*" OR coxib OR coxibs OR mifepristone OR antifibrinolytic* OR "fibrinolysis inhibitor*" OR "aminomethyl cyclohexane carboxylic acid*" OR "aminomethyl cyclohexanecarboxylic acid*" OR "aminomethylcyclohexane carbonic acid*" OR "aminomethylcyclohexane carboxylic acid*" OR "aminomethylcyclohexanecarbonic acid*" OR "aminomethylcyclohexanecarboxylic

acid*" OR "aminomethylcyclohexanocarboxylic acid*" OR "aminomethylcyclohexanoic acid*" OR "tranexamic acid*" OR tranexam OR "tranexanic acid*" OR "transexamic acid*" OR traxamic OR txa)) AND (TITLE-ABS-KEY ("menstrual cycle" OR menstruation OR "uterine bleeding pattern" OR "menstrual cycle length" OR amenorrh?ea OR "menstrual related disorder" OR oligomenorrh?ea OR "menstruation disorder" OR "menstural irregular*" OR "menstrual change*" OR (("contraceptive associated" OR "contraceptive induced") W/2 ("menstrual change*" OR "menstrual irregular*")) OR "dysfunctional uterine bleeding" OR "dysfunctional uterus bleeding" OR "intermenstrual bleeding" OR "intermenstrual h?emo?r?age*" OR "intermenstrual h?em?or*age" OR ((change* OR irregular* OR disturb* OR disorder* OR pattern* OR cycle OR "cycle length") AND "monthly period?") OR ((suffer* OR disabl* OR debilitat* OR burden* OR impact*) W/3 (((change* OR irregular* OR disturb* OR disorder* OR pattern* OR cycle OR "cycle length") AND menstrual) OR "monthly period?")))) AND (TITLE-ABS-KEY (contracepti* OR "birth control" OR "hormon* contracepti*" OR "oral contracepti* agent" OR "birth control implant" OR "intrauterine contracepti* device" OR iud OR iucd OR "injectable contracepti*" OR "contracepti* patch" OR ((hormonal OR copper OR "copper releasing") W/0 (iud OR iuds OR "intrauterine contracepti*")) OR lng-iud OR "levonorgestrel IUD" OR "levonorgestrel releasing intrauterine"))) AND PUBYEAR > 1999 AND PUBYEAR < 2024 AND (EXCLUDE (DOCTYPE , "cp") OR EXCLUDE (DOCTYPE , "le") OR EXCLUDE (DOCTYPE , "ed") OR EXCLUDE (DOCTYPE , "tb"))

Supplementary Table S1E. PsycInfo Database search
Database(s): **APA PsycInfo** 1806 to December 2024

#	Searches
1	Africa/ or Africa south of the Sahara/ or North Africa/ or angola/ or benin/ or Botswana/ or Burkina Faso/ or Burundi/ or Cameroon/ or Cape Verde/ or Central Africa/ or Central African Republic/ or Chad/ or Comoros/ or Congo/ or Cote dIvoire/ or Democratic Republic Congo/ or Djibouti/ or Equatorial Guinea/ or Eritrea/ or Eswatini/ or Ethiopia/ or Gabon/ or Gambia/ or Ghana/ or Guinea/ or Guinea-Bissau/ or Kenya/ or Lesotho/ or Liberia/ or Madagascar/ or Malawi/ or Mali/ or Mozambique/ or Namibia/ or Niger/ or Nigeria/ or Rwanda/ or Sahel/ or Senegal/ or Sierra Leone/ or Somalia/ or South Africa/ or South Sudan/ or Sudan/ or Tanzania/ or Togo/ or Uganda/ or Zambia/ or Zimbabwe/ or Algeria/ or Egypt/ or Libyan Arab Jamahiriya/ or Mauritania/ or Morocco/ or Tunisia/ or Western Sahara/ or Central Africa/ or North Africa/ or African Caribbean/ or Caribbean/ or Central America/ or "South and Central America"/ or Belize/ or Costa Rica/ or El Salvador/ or Guatemala/ or Honduras/ or Nicaragua/ or Panama/ or Antillean/ or Caribbean Islands/ or Cuba/ or Dominica/ or Dominican Republic/ or Grenada/ or Guadeloupe/ or Jamaica/ or Haiti/ or Martinique/ or Saint Lucia/ or "Saint Vincent and the Grenadines"/ or "caribbean (person)"/ or Cuban/ or "dominican (dominica)"/ or "dominican (dominican republic)"/ or Haitian/ or Jamaican/ or South America/ or Argentina/ or Bolivia/ or Brazil/ or Colombia/ or Ecuador/ or French Guiana/ or Guyana/ or Paraguay/ or Peru/ or Suriname/ or Venezuela/ or Mexico/ or Asia/ or central Asia/ or Far East/ or Middle East/ or northern Asia/ or South Asia/ or western Asia/ or Kazakhstan/ or Kyrgyzstan/ or Tajikistan/ or Turkmenistan/ or Uzbekistan/ or China/ or Korea/ or Mongolia/ or Philippines/ or Southeast Asia/ or North Korea/ or Borneo/ or Cambodia/ or Indonesia/ or Laos/ or Malaysia/ or Myanmar/ or Papua New Guinea/ or Singapore/ or Thailand/ or Timor-Leste/ or Viet Nam/ or Iran/ or Iraq/ or Jordan/ or Lebanon/ or Palestine/ or Syrian Arab Republic/ or "turkey (republic)"/ or Yemen/ or Afghanistan/ or Bangladesh/ or Bhutan/ or India/ or Nepal/ or Pakistan/ or Sri Lanka/ or Armenia/ or Azerbaijan/ or "georgia (republic)"/ or "Sao Tome and Principe"/ or Mauritius/ or Pacific Islands/ or Federated States of Micronesia/ or Fiji/ or Kiribati/ or Marshall Islands/ or Melanesia/ or Nauru/ or Palau/ or Polynesia/ or Samoan Islands/ or Solomon Islands/ or Timor-Leste/ or Tonga/ or Tuvalu/ or Vanuatu/ or American Samoa/ or Samoa/ or Romania/ or Russian Federation/ or USSR/ or Croatia/ or Albania/ or Belarus/ or "Bosnia and Herzegovina"/ or Bulgaria/ or Kosovo/ or Moldova/ or "Montenegro (republic)"/ or Republic of North Macedonia/ or Serbia/ or Ukraine/ or "Federation of Bosnia and Herzegovina"/
2	(Afghanistan* or Albania* or Algeria* or Angola* or Argentina* or Armenia* or Azerbaijan* or Bangladesh* or Beliz* or Benin* or Bhutan* or Bolivia* or Bosnia* or Herzegovin* or Botswan* or Brazil* or Bulgaria* or Burkina* or Burundi* or Cabo Verde* or Cape Verde* or Cambodia* or Cameroon* or Chad* or China or Chinese or Colombia* or Comor* or Congo* or Costa Rica* or Cote d'Ivoir* or Ivory Coast or Cuba* or Djibouti* or Dominica* or Ecuador* or Egypt* or El Salvador* or Eritrea* or Ethiopia* or Fiji* or Gabon* or Gambia* or Georgia* or Ghana* or Grenad* or Guatemala* or Guinea* or Guyan* or Haiti* or Hondura* or Hungar* or India* or Indonesia* or Iran* or Iraq* or Jamaica* or Jordan* or Kazakhstan* or Kenya* or Kiribati* or Korea* or Kosov* or Kyrgyz* or Lao* or Leban* or Lesotho* or Liberia* or Libya* or Macedonia* or Madagascar* or Malawi* or Malaysia* or Maldiv* or Mali* or Marshall Island* or Mauritania* or Mauriti* or Mexic* or Moldova* or Mongolia* or Montenegr* or Morocc* or Mozambi* or Myanma* or Burmese or Namibia* or Nepal* or Nicaragua* or Niger* or Pakistan* or Palau* or Panama* or Papua New Guinea* or Paraguay* or Peru* or Philippines or Filipino or Romania* or Rwanda* or Samoa* or Sao Tome* or Senegal* or Serbia* or Seychell* or Sierra Leon* or Solomon Island* or Somalia* or Sudan* or Sri Lanka* or St Lucia* or Saint Lucia or St Vincent or Saint Vincent or Grenadines or Surinam* or Swazi* or Syria* or Tajikistan* or Tanzania* or Thai* or Timor* or Togo* or Tonga* or Tunisia* or Turk* or Tuvalu* or Uganda* or Ukrain* or Uzbekistan* or Vanuatu* or Venezuela* or Vietnam* or Viet-Nam* or West Bank or Gaza or Yemen* or Zambia* or Zimbabwe* or Russia* or Croatia* or Nauru* or Yugoslavia* or USSR or Soviet* or Byelarus* or Belarus*).mp.
3	(africa* or asia* or caribbean or central america* or latin america* or south america* or melanesia* or micronesia* or polynesia*).mp.
4	(resource-limit* or resource-poor or low-resource* or limited-resource* or resource-constrain* or constrain*-resource* or under-resource* or poor*-resource* or resource-scarce* or scarce*-resource* or low-income or middle-income or lowincome or middleincome or (low* adj3 middle-income)).mp.
5	((developing or underdeveloped or under-developed or emerging or less-developed or least-developed or less-economically developed or least-economically developed or less-affluent or least-affluent or least-industriali#ed or non-industriali#ed or deprived or poor) adj (country or countries or nation? or region? or economy or economies)).mp.

6	((developing or underdeveloped or under-developed or less-developed or least-developed) adj (population* or world)).mp.
7	(third-world* or thirdworld* or 3rd-world* or lmic or lmics or lami countr* or lalmi countr* or transitional countr*).mp.
8	(low* adj (gdp or gnp or gross domestic or gross national)).mp.
9	((underserved or under-served) adj (countr* or nation? or population*)).mp.
10	1 or 2 or 3 or 4 or 5 or 6 or 7 or 8 or 9
11	contraception/ or birth control/ or hormonal contraception/ or long-acting reversible contraception/ or oral contraception/ or ovulation inhibition/
12	(contracepti* or anticonceptive or antifertility or antiovulatory or anti-conceptive* or anti-fertility or anti-ovulatory).mp.
13	((intrauterine or intracervical or intra-uterine or intra-cervical) adj (device* or coil*)).mp.
14	((hormonal or copper or copper releasing) adj (IUD or IUDs)).mp.
15	(LNG-IUD or levonorgestrel IUD* or levonorgestrel releasing intrauterine).mp.
16	(anovulatory agent* or anovulatory drug* or ovulation inhibitor* or ovulation block* or ovulat* inhibiting hormone* or ovulat* suppression).mp.
17	11 or 12 or 13 or 14 or 15 or 16
18	menstrual cycle/ or menstruation/ or uterine bleeding pattern/ or menstrual cycle length/
19	"amenorrhea and oligomenorrhea"/ or menstruation disorder/ or amenorrhea/ or menstrual irregularity/ or oligomenorrhea/ or dysmenorrhea/ or "menorrhagia and metrorrhagia"/ or menstrual related disorder/ or menorrhagia/ or metrorrhagia/ or spotting/ or menometrorrhagia/
20	(menstrua* or menses or catamenia or menstruum or eumeno?rh?ea* or meno?rh?ea*).mp.
21	(ameno?rh?ea* or amenor*ea or amenia or hypomeno?rh?ea* or hypomenor*ea or oligomeno?rh?oea* or oligomenor*ea or spaniomeno?rh?ea* or spaniomenor*ea or spanomeno?rh?ea* or spanomenor*ea or meno?rhagi* or menorr?agi* or menor*agi* or hypermeno?rhea* or hypermenorr?oea* or hypermeno?rh?ea* or meno?rhagy or menorr?agy or polymenor?hea* or polymenorr?ea or polymeno?rh?ea or menometro?rhagi* or menometrorr?agi* or menometror*agi? or metro?rhag* or metror*agy or metro?rh?ea* or metror*ea).mp.
22	(dysfunctional uterine bleeding or dysfunctional uterus bleeding or intermenstrual bleeding or intermenstrual h?emo?r?age* or intermenstrual h?em?or*age).mp.
23	((bleed* or bled or blood*) and monthly period?).mp.
24	((heavy or light) adj period?).mp.
25	((change* or irregular* or disturb* or disorder* or pattern* or cycle or cycle length) and monthly period?).mp.
26	((suffer* or disabl* or debilitat* or burden* or impact*) adj3 (((change* or irregular* or disturb* or disorder* or pattern* or cycle or cycle length) and menstrual) or monthly period?)).mp. [mp=title, abstract, heading word, table of contents, key concepts, original title, tests & measures, mesh word]
27	18 or 19 or 20 or 21 or 22 or 23 or 24 or 25 or 26
28	(non-steroid* antiinflammatory or non-steroid* anti-inflammatory or nonsteroid antiinflammatory or nonsteroid* anti-inflammatory or NSAID or NSAIDs).mp.
29	(cyclooxygenase inhibitor* or cyclo-oxygenase inhibitor* or prostaglandin synthetase inhibitor* or cyclooxygenase-2 inhibitor* or COX-2 inhibitor* or COX-2 specific inhibitor* or COX2 inhibitor* or COX2 specific inhibitor* or coxib or coxibs).mp.
30	mifepristone.mp.
31	(antifibrinolytic* or fibrinolysis inhibitor* or aminomethyl cyclohexane carboxylic acid* or aminomethyl cyclohexanecarboxylic acid* or aminomethylcyclohexane carbonic acid* or aminomethylcyclohexane carboxylic acid* or aminomethylcyclohexanecarbonic acid* or aminomethylcyclohexanecarboxylic acid* or aminomethylcyclohexanocarboxylic acid* or aminomethylcyclohexanoic acid* or tranexamic acid* or tranexam or tranexanic acid* or transexamic acid* or traxamic or TXA).mp.
32	28 or 29 or 30 or 31
33	10 and 17 and 27
34	17 and 27 and 32
35	33 or 34
36	limit 35 to yr="2000 -Current"

Supplementary Table S1F. Global Health Database search

Database(s): **Global Health** 1910 to 2024 Week 49

#	Searches
1	Africa/ or Africa south of the Sahara/ or North Africa/ or angola/ or benin/ or Botswana/ or Burkina Faso/ or Burundi/ or Cameroon/ or Cape Verde/ or Central Africa/ or Central African Republic/ or Chad/ or Comoros/ or Congo/ or Cote dIvoire/ or Democratic Republic Congo/ or Djibouti/ or Equatorial Guinea/ or Eritrea/ or Eswatini/ or Ethiopia/ or Gabon/ or Gambia/ or Ghana/ or Guinea/ or Guinea-Bissau/ or Kenya/ or Lesotho/ or Liberia/ or Madagascar/ or Malawi/ or Mali/ or Mozambique/ or Namibia/ or Niger/ or Nigeria/ or Rwanda/ or Sahel/ or Senegal/ or Sierra Leone/ or Somalia/ or South Africa/ or South Sudan/ or Sudan/ or Tanzania/ or Togo/ or Uganda/ or Zambia/ or Zimbabwe/ or Algeria/ or Egypt/ or Libyan Arab Jamahiriya/ or Mauritania/ or Morocco/ or Tunisia/ or Western Sahara/ or Central Africa/ or North Africa/ or African Caribbean/ or Caribbean/ or Central America/ or "South and Central America"/ or Belize/ or Costa Rica/ or El Salvador/ or Guatemala/ or Honduras/ or Nicaragua/ or Panama/ or Antillean/ or Caribbean Islands/ or Cuba/ or Dominica/ or Dominican Republic/ or Grenada/ or Guadeloupe/ or Jamaica/ or Haiti/ or Martinique/ or Saint Lucia/ or "Saint Vincent and the Grenadines"/ or "caribbean (person)"/ or Cuban/ or "dominican (dominica)"/ or "dominican (dominican republic)"/ or Haitian/ or Jamaican/ or South America/ or Argentina/ or Bolivia/ or Brazil/ or Colombia/ or Ecuador/ or French Guiana/ or Guyana/ or Paraguay/ or Peru/ or Suriname/ or Venezuela/ or Mexico/ or Asia/ or central Asia/ or Far East/ or Middle East/ or northern Asia/ or South Asia/ or western Asia/ or Kazakhstan/ or Kyrgyzstan/ or Tajikistan/ or Turkmenistan/ or Uzbekistan/ or China/ or Korea/ or Mongolia/ or Philippines/ or Southeast Asia/ or North Korea/ or Borneo/ or Cambodia/ or Indonesia/ or Laos/ or Malaysia/ or Myanmar/ or Papua New Guinea/ or Singapore/ or Thailand/ or Timor-Leste/ or Viet Nam/ or Iran/ or Iraq/ or Jordan/ or Lebanon/ or Palestine/ or Syrian Arab Republic/ or "turkey (republic)"/ or Yemen/ or Afghanistan/ or Bangladesh/ or Bhutan/ or India/ or Nepal/ or Pakistan/ or Sri Lanka/ or Armenia/ or Azerbaijan/ or "georgia (republic)"/ or "Sao Tome and Principe"/ or Mauritius/ or Pacific Islands/ or Federated States of Micronesia/ or Fiji/ or Kiribati/ or Marshall Islands/ or Melanesia/ or Nauru/ or Palau/ or Polynesia/ or Samoan Islands/ or Solomon Islands/ or Timor-Leste/ or Tonga/ or Tuvalu/ or Vanuatu/ or American Samoa/ or Samoa/ or Romania/ or Russian Federation/ or USSR/ or Croatia/ or Albania/ or Belarus/ or "Bosnia and Herzegovina"/ or Bulgaria/ or Kosovo/ or Moldova/ or "Montenegro (republic)"/ or Republic of North Macedonia/ or Serbia/ or Ukraine/ or "Federation of Bosnia and Herzegovina"/
2	(Afghanistan* or Albania* or Algeria* or Angola* or Argentina* or Armenia* or Azerbaijan* or Bangladesh* or Beliz* or Benin* or Bhutan* or Bolivia* or Bosnia* or Herzegovin* or Botswan* or Brazil* or Bulgaria* or Burkina* or Burundi* or Cabo Verde* or Cape Verde* or Cambodia* or Cameroon* or Chad* or China or Chinese or Colombia* or Comor* or Congo* or Costa Rica* or Cote d'Ivoir* or Ivory Coast or Cuba* or Djibouti* or Dominica* or Ecuador* or Egypt* or El Salvador* or Eritrea* or Ethiopia* or Fiji* or Gabon* or Gambia* or Georgia* or Ghana* or Grenad* or Guatemala* or Guinea* or Guyan* or Haiti* or Hondura* or Hungar* or India* or Indonesia* or Iran* or Iraq* or Jamaica* or Jordan* or Kazakhstan* or Kenya* or Kiribati* or Korea* or Kosov* or Kyrgyz* or Lao* or Leban* or Lesotho* or Liberia* or Libya* or Macedonia* or Madagascar* or Malawi* or Malaysia* or Maldiv* or Mali* or Marshall Island* or Mauritania* or Mauriti* or Mexic* or Moldova* or Mongolia* or Montenegr* or Morocc* or Mozambi* or Myanma* or Burmese or Namibia* or Nepal* or Nicaragua* or Niger* or Pakistan* or Palau* or Panama* or Papua New Guinea* or Paraguay* or Peru* or Philippines or Filipino or Romania* or Rwanda* or Samoa* or Sao Tome* or Senegal* or Serbia* or Seychell* or Sierra Leon* or Solomon Island* or Somalia* or Sudan* or Sri Lanka* or St Lucia* or Saint Lucia or St Vincent or Saint Vincent or Grenadines or Surinam* or Swazi* or Syria* or Tajikistan* or Tanzania* or Thai* or Timor* or Togo* or Tonga* or Tunisia* or Turk* or Tuvalu* or Uganda* or Ukrain* or Uzbekistan* or Vanuatu* or Venezuela* or Vietnam* or Viet-Nam* or West Bank or Gaza or Yemen* or Zambia* or Zimbabwe* or Russia* or Croatia* or Nauru* or Yugoslavia* or USSR or Soviet* or Byelarus* or Belarus*).mp.
3	(africa* or asia* or caribbean or central america* or latin america* or south america* or melanesia* or micronesia* or polynesia*).mp.
4	(resource-limit* or resource-poor or low-resource* or limited-resource* or resource-constrain* or constrain*-resource* or under-resource* or poor*-resource* or resource-scarce* or scarce*-resource* or low-income or middle-income or lowincome or middleincome or (low* adj3 middle-income)).mp.
5	((developing or underdeveloped or under-developed or emerging or less-developed or least-developed or less-economically developed or least-economically developed or less-affluent or least-affluent or least-industriali#ed or non-industriali#ed or deprived or poor) adj (country or countries or nation? or region? or economy or economies)).mp.

6	((developing or underdeveloped or under-developed or less-developed or least-developed) adj (population* or world)).mp.
7	(third-world* or thirdworld* or 3rd-world* or lmic or lmics or lami countr* or lalmi countr* or transitional countr*).mp.
8	(low* adj (gdp or gnp or gross domestic or gross national)).mp.
9	((underserved or under-served) adj (countr* or nation? or population*)).mp.
10	1 or 2 or 3 or 4 or 5 or 6 or 7 or 8 or 9
11	contraception/ or birth control/ or hormonal contraception/ or long-acting reversible contraception/ or oral contraception/ or ovulation inhibition/
12	(contracepti* or anticonceptive or antifertility or antiovulatory or anti-conceptive* or anti-fertility or anti-ovulatory).mp.
13	((intrauterine or intracervical or intra-uterine or intra-cervical) adj (device* or coil*)).mp.
14	((hormonal or copper or copper releasing) adj (IUD or IUDs)).mp.
15	(LNG-IUD or levonorgestrel IUD* or levonorgestrel releasing intrauterine).mp.
16	(anovulatory agent* or anovulatory drug* or ovulation inhibitor* or ovulation block* or ovulat* inhibiting hormone* or ovulat* suppression).mp.
17	11 or 12 or 13 or 14 or 15 or 16
18	menstrual cycle/ or menstruation/ or uterine bleeding pattern/ or menstrual cycle length/
19	(menstrua* or menses or catamenia or menstruum or eumeno?rh?ea* or meno?rh?ea*).mp.
20	(ameno?rh?ea* or amenor*ea or amenia or hypomeno?rh?ea* or hypomenor*ea or oligomeno?rh?oea* or oligomenor*ea or spaniomeno?rh?ea* or spaniomenor*ea or spanomeno?rh?ea* or spanomenor*ea or meno?rhagi* or menorr?agi* or menor*agi* or hypermeno?rhea* or hypermenorr?oea* or hypermeno?rh?ea* or meno?rhagy or menorr?agy or polymenor?hea* or polymenorr?ea or polymeno?rh?ea or menometro?rhagi* or menometrorr?agi* or menometror*agi? or metro?rhag* or metror*agy or metro?rh?ea* or metror*ea).mp.
21	(dysfunctional uterine bleeding or dysfunctional uterus bleeding or intermenstrual bleeding or intermenstrual h?emo?r?age* or intermenstrual h?em?or*age).mp.
22	((bleed* or bled or blood*) and monthly period?).mp.
23	((heavy or light) adj period?).mp.
24	((change* or irregular* or disturb* or disorder* or pattern* or cycle or cycle length) and monthly period?).mp.
25	((suffer* or disabl* or debilitat* or burden* or impact*) adj3 (((change* or irregular* or disturb* or disorder* or pattern* or cycle or cycle length) and menstrual) or monthly period?)).mp. [mp=abstract, title, original title, heading words, cabicodes words]
26	18 or 19 or 20 or 21 or 22 or 23 or 24 or 25
27	(non-steroid* antiinflammatory or non-steroid* anti-inflammatory or nonsteroid antiinflammatory or nonsteroid* anti-inflammatory or NSAID or NSAIDs).mp.
28	(cyclooxygenase inhibitor* or cyclo-oxygenase inhibitor* or prostaglandin synthetase inhibitor* or cyclooxygenase-2 inhibitor* or COX-2 inhibitor* or COX-2 specific inhibitor* or COX2 inhibitor* or COX2 specific inhibitor* or coxib or coxibs).mp.
29	mifepristone.mp.
30	(antifibrinolytic* or fibrinolysis inhibitor* or aminomethyl cyclohexane carboxylic acid* or aminomethyl cyclohexanecarboxylic acid* or aminomethylcyclohexane carbonic acid* or aminomethylcyclohexane carboxylic acid* or aminomethylcyclohexanecarbonic acid* or aminomethylcyclohexanecarboxylic acid* or aminomethylcyclohexanocarboxylic acid* or aminomethylcyclohexanoic acid* or tranexamic acid* or tranexam or tranexanic acid* or transexamic acid* or traxamic or TXA).mp.
31	27 or 28 or 29 or 30
32	10 and 17 and 26
33	17 and 26 and 31
34	32 or 33
35	limit 34 to yr="2000 -Current"

Supplementary Table S1G. Global Index Medicus Database search

tw:((tw:((contraceptive OR "birth control" OR implant OR patch OR "family planning" OR "the pill" OR "long-acting contraceptive" OR levonorgestrel OR iud OR iucd OR "intrauterine device"))) AND (tw:(("menstrual irregularity" OR “menstrual change” OR "contraceptive-induced menstrual change" OR "contraceptive-associated menstrual irregularity" OR "menstruation disorder" OR "intermenstrual bleeding" OR "breakthrough bleeding" OR "heavy menstrual bleeding" OR "dysfunctional uterine bleeding" OR "menstrual disturbances")))) AND (year_cluster:[2000 TO 2024])

Supplementary Table S1H. Web of Science Database search

Search: low* NEAR/0 (gdp OR gnp OR "gross domestic" OR "gross national") (Topic) OR (underserved OR "under-served") NEAR/0 (countr* OR nation? OR population*) (Topic) OR "resource-limit*" OR "resource-poor" OR "low-resource*" OR "limited-resource*" OR "resource-constrain*" OR "constrain*-resource*" OR "under-resource*" OR "poor*-resource*" OR "resource-scarce*" OR "scarce*-resource*" OR "low-income" OR "middle-income" OR lowincome OR middleincome OR (low* NEAR/2 "middle-income") (Topic) OR "third-world*" OR thirdworld* OR "3rd-world*" OR lmic OR lmics OR "lami countr*" OR "lalmi countr*" OR "transitional countr*" (Topic) OR (developing OR underdeveloped OR "under-developed" OR "less-developed" OR "least-developed") NEAR/0 (population* OR world) (Topic) OR (developing OR underdeveloped OR "under-developed" OR emerging OR "less-developed" OR "least-developed" OR "less-economically developed" OR "least-economically developed" OR "less-affluent" OR "least-affluent" OR "least-industriali?ed" OR "non-industriali?ed" OR deprived OR poor) NEAR/0 (country OR countries OR nation? OR region? OR economy OR economies) (Topic) OR afghanistan* OR albania* OR algeria* OR angola* OR argentina* OR armenia* OR azerbaijan* OR bangladesh* OR beliz* OR benin* OR bhutan* OR bolivia* OR bosnia* OR herzegovin* OR botswan* OR brazil* OR bulgaria* OR burkina* OR burundi* OR "Cabo Verde*" OR "Cape Verde*" OR cambodia* OR cameroon* OR chad* OR china OR chinese OR colombia* OR comor* OR congo* OR "Costa Rica*" OR "Cote d'Ivoir*" OR "Ivory Coast" OR cuba* OR djibouti* OR dominica* OR ecuador* OR egypt* OR "El Salvador*" OR eritrea* OR ethiopia* OR fiji* OR gabon* OR gambia* OR georgia* OR ghana* OR grenad* OR guatemala* OR guinea* OR guyan* OR haiti* OR hondura* OR hungar* OR india* OR indonesia* OR iran* OR iraq* OR jamaica* OR jordan* OR kazakhstan* OR kenya* OR kiribati* OR korea* OR kosov* OR kyrgyz* OR lao* OR leban* OR lesotho* OR liberia* OR libya* OR macedonia* OR madagascar* OR malawi* OR malaysia* OR maldiv* OR mali* OR "Marshall Island*" OR mauritania* OR mauriti* OR mexic* OR moldova* OR mongolia* OR montenegr* OR morocc* OR mozambi* OR myanma* OR burmese OR namibia* OR nepal* OR nicaragua* OR niger* OR pakistan* OR palau* OR panama* OR "Papua New Guinea*" OR paraguay* OR peru* OR philippines OR filipino OR romania* OR rwanda* OR samoa* OR "Sao Tome*" OR senegal* OR serbia* OR seychell* OR "Sierra Leon*" OR "Solomon Island*" OR somalia* OR sudan* OR "Sri Lanka*" OR "St Lucia*" OR "Saint Lucia" OR "St Vincent" OR "Saint Vincent" OR grenadines OR surinam* OR swazi* OR syria* OR tajikistan* OR tanzania* OR thai* OR timor* OR togo* OR tonga* OR tunisia* OR turk* OR tuvalu* OR uganda* OR ukrain* OR uzbekistan* OR vanuatu* OR venezuela* OR vietnam* OR "Viet-Nam*" OR "West Bank" OR gaza OR yemen* OR zambia* OR zimbabwe* OR russia* OR croatia* OR nauru* OR yugoslavia* OR ussr OR soviet* OR byelarus* OR belarus* (Topic)<br><br>Search: (TS=("menstrual cycle" OR menstruation OR "uterine bleeding pattern" OR "menstrual cycle length" OR amenorrh?ea OR "menstrual related disorder" OR oligomenorrh?ea OR "menstruation disorder" OR "menstural irregular*" OR "menstrual change*" OR (("contraceptive associated" OR "contraceptive induced") NEAR/2 ("menstrual change*" OR "menstrual irregular*")) OR "dysfunctional uterine bleeding" OR "dysfunctional uterus bleeding" OR "intermenstrual bleeding" OR "intermenstrual h?emo?r?age*" OR "intermenstrual h?em?or*age" OR ((change* OR irregular* OR disturb* OR disorder* OR pattern* OR cycle OR "cycle length") AND "monthly period?")) OR AU=(((suffer* OR disabl* OR debilitat* OR burden* OR impact*) NEAR/3 (change* OR irregular* OR disturb* OR disorder* OR pattern* OR cycle OR "cycle length") AND menstrual)))<br><br>Search: (TS=(((contracepti* OR "birth control" OR "hormon* contracepti*" OR "oral contracepti* agent" OR "birth control implant" OR "intrauterine contracepti* device" OR iud OR iucd OR "injectable contracepti*" OR "contracepti* patch" OR ((hormonal OR copper OR "copper releasing") NEAR/0 (iud OR iuds OR "intrauterine contracepti*")) OR lng-iud OR "levonorgestrel IUD" OR "levonorgestrel releasing intrauterine")))) Search: (TS=((("nonsteroid* anti-inflammatory" OR "non-steroid* anti-inflammatory" OR "nonsteroid anti-inflammatory" OR "nonsteroid* anti-inflammatory" OR nsaid OR nsaids OR "cyclooxygenase inhibitor*" OR "cyclo-oxygenase inhibitor*" OR "prostaglandin synthetase inhibitor*" OR "cyclooxygenase-2 inhibitor*" OR "COX-2 inhibitor*" OR "COX-2 specific inhibitor*" OR "COX2 inhibitor*" OR "COX2 specific inhibitor*" OR coxib OR coxibs OR mifepristone OR antifibrinolytic* OR "fibrinolysis inhibitor*" OR "aminomethyl cyclohexane

carboxylic acid*" OR "aminomethyl cyclohexanecarboxylic acid*" OR "aminomethylcyclohexane carbonic acid*" OR "aminomethylcyclohexane carboxylic acid*" OR "aminomethylcyclohexanecarbonic acid*" OR "aminomethylcyclohexanecarboxylic acid*" OR "aminomethylcyclohexanocarboxylic acid*" OR "aminomethylcyclohexanoic acid*" OR "tranexamic acid*" OR tranexam OR "tranexanic acid*" OR "transexamic acid*" OR traxamic OR txa))))
Database: All Databases
Entitlements:
- WOS: 1900 to 2024
- BIOABS: 1980 to 2024
- BIOSIS: 1926 to 2024
- CSCD: 1989 to 2024
- CCC: 1998 to 2024
- DRCI: 1900 to 2024
- KJD: 1980 to 2024
- MEDLINE: 1950 to 2024
- PPRN: 1991 to 2024
- SCIELO: 2002 to 2024
Searches:
Search: (#4 OR #6) and Editorial Material or Abstract or Letter or Retracted Publication (Exclude – Document Types)

Supplementary Table S1I. CINAHL Database search

December 16, 2024	Query	Limiters/Expanders	Last Run Via
S18	s16 not s17	Limiters<br>- PublishedDate: 20000101-20241231<br>Search modes<br>-Boolean/Phrase	Interface<br>- EBSCOhostResearch Databases<br>Search Screen<br>- AdvancedSearch<br>Database<br>- CINAHL Complete
S17	PT Commentary orDoctoral Dissertation orEditorial or Letter orMasters Thesis	Search modes<br>-Boolean/Phrase	Interface<br>- EBSCOhostResearch Databases<br>Search Screen<br>- AdvancedSearch<br>Database<br>- CINAHL Complete
S16	s14 not s15	Search modes<br>-Boolean/Phrase	Interface<br>- EBSCOhostResearch Databases<br>Search Screen<br>- AdvancedSearch<br>Database<br>- CINAHL Complete
S15	(((MH "Animals+") OR(MH "Animal Studies")OR (TI "animal model*"))NOT (MH "human"))	Search modes<br>-Boolean/Phrase	Interface<br>- EBSCOhostResearch Databases<br>Search Screen<br>- AdvancedSearch<br>Database<br>- CINAHL Complete
S14	S12 OR S13	Search modes<br>-Boolean/Phrase	Interface<br>- EBSCOhostResearch Databases<br>Search Screen<br>- AdvancedSearch<br>Database<br>- CINAHL Complete
S13	S9 AND S10 AND S11	Search modes<br>-Boolean/Phrase	Interface<br>- EBSCOhostResearch Databases<br>Search Screen<br>- AdvancedSearch<br>Database<br>- CINAHL Complete
S12	S4 AND S9 AND S10	Search modes<br>-Boolean/Phrase	Interface<br>- EBSCOhostResearch Databases<br>Search Screen<br>- AdvancedSearch<br>Database<br>- CINAHL Complete

S11	(nonsteroid* anti-inflammatory drugs ornsaid?) OR (tranexamicacid or txa orantifibinylotic) OR ((cyclooxygenaseinhibitor* or cyclo-oxygenase inhibitor* orprostaglandin synthetaseinhibitor* orcyclooxygenase-2inhibitor* or COX-2inhibitor* or COX-2specific inhibitor* orCOX2 inhibitor* or COX2specific inhibitor* or coxibor coxibs)) ORmifepristone	Search modes -Boolean/Phrase	Interface - EBSCOhostResearch Databases Search Screen - AdvancedSearch Database - CINAHL Complet
S10	MH (contraception orbirth control or familyplanning or contraceptive) OR (oral contraceptivepills or birth control pillsor ocp or oralcontraceptives orcontraceptives) ORintrauterine device ORcontracepti* use OR birthcontrol implant ORcontracept* behavio?r	Search modes -Boolean/Phrase	Interface - EBSCOhostResearch Databases Search Screen - AdvancedSearch Database - CINAHL Complete
S9	S5 OR S6 OR S7 OR S8	Search modes -Boolean/Phrase	Interface - EBSCOhostResearch Databases Search Screen - AdvancedSearch Database - CINAHL Complete
S8	((suffer* or disabl* ordebilitat* or burden* orimpact*) N4 (((change*or irregular* or disturb* ordisorder* or pattern* orcycle or cycle length) andmenstrual) or monthlyperiod?)).	Search modes -Boolean/Phrase	Interface - EBSCOhostResearch Databases Search Screen - AdvancedSearch Database - CINAHL Complete
S7	MH (menstrual cycle orperiod or menstruation ormenses) OR (amenorrhea oroligomenorrhea ormenstrual disturbance)OR (menorrhagia orheavy bleeding) ORdysfunctional uterinebleeding ORintermenstrual bleedingOR intermenstrual h?emo?r?age OR uterinebleeding pattern	Search modes -Boolean/Phrase	Interface - EBSCOhostResearch Databases Search Screen - AdvancedSearch Database - CINAHL Complete
S6	irregular menstruationOR irregular menses ORirregular menstrual cycle	Search modes -Boolean/Phrase	Interface - EBSCOhostResearch Databases Search Screen - AdvancedSearch Database - CINAHL Complete
S5	menstrual irregularit*	Search modes -Boolean/Phrase	Interface - EBSCOhostResearch Databases Search Screen - AdvancedSearch Database - CINAHL Complete

S4	S1 or S2 or S3	Search modes -Boolean/Phrase	Interface - EBSCOhostResearch Databases Search Screen - AdvancedSearch Database - CINAHL Complete
S3	(("third-world*" orthirdworld* or "3rd-world*" or lmic or lmics or"lami countr*" or "lalmicountr*" or "transitionalcountr*")) OR ((low* N0(gdp or gnp or "grossdomestic" or "grossnational"))) OR (((underserved or "under-served") N0 (countr* ornation? or population*)))	Search modes -Boolean/Phrase	Interface - EBSCOhostResearch Databases Search Screen - AdvancedSearch Database - CINAHL Complete
S2	(("resource-limit*" or"resource-poor" or "low-resource*" or "limited-resource*" or "resource-constrain*" or"constrain*-resource*" or"under-resource*" or"poor*-resource*" or"resource-scarce*" or"scarce*-resource*" or"low-income" or "middle-income" or lowincome ormiddleincome or (low*N2 "middle-income")))OR (((developing orunderdeveloped or"under-developed" oremerging or "less-developed" or "least-developed" or "less-economically developed"or "least-economicallydeveloped" or "less-affluent" or "least-affluent" or "least-industriali#ed" or "non-industriali#ed" ordeprived or poor) N0(country or countries ornation? or region? oreconomy or economies))) OR (((developing orunderdeveloped or"under-developed" or"less-developed" or"least-developed") N0(population* or world)))	Search modes -Boolean/Phrase	Interface - EBSCOhostResearch Databases Search Screen - Advanced Search Database - CINAHL Complete
S1	((MH "DevelopingCountries") OR (MH"Low and Middle IncomeCountries" OR MH"Developing countries"OR MH "Africa" OR MH"Africa South of theSahara" OR MH "Africa,Western" OR MH "Africa,Southern" OR MH"Africa, Northern" OR MH"Africa, Eastern" OR MH "Guinea" OR MH"Guinea-Bissau" OR MH"Liberia" OR MH "Mali"OR MH "Mauritania" ORMH "Niger" OR MH"Nigeria" OR MH"Senegal" OR MH "SierraLeone" OR MH "Togo""Africa, Central" OR MH"South Africa" OR MH"Namibia" OR MH"Algeria" OR MH "Egypt"OR MH "Libya" OR MH"Morocco" OR MH"Tunisia" OR MH"Cameroon" OR MH"Central AfricanRepublic" OR MH "Chad"OR MH "Congo" OR MH"Democratic Republic ofthe Congo" OR MH"Equatorial Guinea" ORMH "Gabon" OR MH"Burundi" OR MH"Djibouti" OR MH"Eritrea" OR MH"Ethiopia" OR MH"Kenya" OR MH"Rwanda" OR MH"Somalia" OR MH"Sudan" OR MH"Tanzania" OR MH"Uganda" OR MH"Angola" OR MH"Botswana" OR MH"Lesotho" OR MH"Malawi" OR MH"Mozambique" OR MH"Swaziland" OR MH"Zambia" OR	Search modes -Boolean/Phrase	Interface - EBSCOhostResearch Databases Search Screen - AdvancedSearch Database - CINAHL Complete

MH“Zimbabwe” OR MH“Benin” OR MH “BurkinaFaso” OR MH “CapeVerde” OR MH “Coted’Ivoire” OR MH“Gambia” OR MH“Ghana” OR MH “East Timor” OR MH“Indonesia” OR MH“Laos” OR MH“Malaysia” OR MH“Myanmar” OR MH“Philippines” OR MH“Thailand” OR MH“Timor” OR MHOR MH “West Indies” ORMH “Cuba” OR MH“Dominica” OR MH“Dominican Republic”OR MH “Haiti” OR MH“Jamaica” OR MH“Martinique” OR MH“Central America” ORMH “Belize” OR MH“Costa Rica” OR MH “ElSalvador” OR MH“Guatemala” OR MH“Honduras” OR MH“Nicaragua” OR MH“Panama” OR MH “LatinAmerica” OR MH“Mexico” OR MH “SouthAmerica” OR MH“Argentina” OR MH“Bolivia” OR MH “Brazil”OR MH “Colombia” ORMH “Ecuador” OR MH“French Guiana” OR MH“Guyana” OR MH“Paraguay” OR MH“Peru” OR MH“Suriname” OR MH“Venezuela” OR MH“Asia” OR MH “Asia,Western” OR MH “Asia,Southeastern” OR MH“Asia, Central” OR MH“Kazakhstan” OR MH“Kyrgyzstan” OR MH“Tajikistan” OR MH“Turkmenistan” OR MH“Uzbekistan” OR MH“Borneo” OR MH“Cambodia” OR MH “Azerbaijan” or MH“Georgia (republic)”))OR ((Afghanistan* orAlbania* or Algeria* orAngola* or Argentina* orArmenia* or Azerbaijan*or Bangladesh* or Beliz*or Benin* or Bhutan* or“Vietnam” OR MH“Bangladesh” OR MH“Bhutan” OR MH “India”OR MH “Middle East” ORMH “Afghanistan” ORMH “Iran” OR MH “Iraq”OR MH “Jordan” OR MH“Lebanon” OR MH“Syria” OR MH “Turkey”OR MH “Yemen” OR MH“Nepal” OR MH“Pakistan” OR MH “SriLanka” OR MH “FarEast” OR MH “China” ORMH “Mongolia” OR MH“North Korea” OR MH“Indian Ocean Islands”OR MH “Madagascar”OR MH “Pacific Islands”OR MH “Melanesia” ORMH “Micronesia” OR MH“Polynesia” OR MH“Papua New Guinea” ORMH “Samoa” OR MH“American Samoa” ORMH “Independent Stateof Samoa” OR MH“Romania” OR MH“Russia” OR MH “USSR”OR MH “Croatia” or MH“Albania” or MH “Bosnia-Herzegovina” or MH“Bulgaria” or MH“Byelarus” or MH“Macedonia (Republic)”or MH “Moldova” or MH“Serbia” or MH “Ukraine”or MH “Yugoslavia” orMH “Armenia” or MH or Burmese or Namibia*or Nepal* or Nicaragua*or Niger* or Pakistan* orPalau* or Panama* or“Papua New Guinea*” orParaguay* or Peru* orPhilippines or Filipino orRomania* or Rwanda* orBolivia* or Bosnia* orHerzegovin* or Botswan*or Brazil* or Bulgaria* orBurkina* or Burundi* or“Cabo Verde*” or “CapeVerde*” or Cambodia* orCameroon* or Chad* orChina or Chinese orColombia* or Comor* orCongo* or

“Costa Rica*”or “Cote d'Ivoir*” or “IvoryCoast” or Cuba* orDjibouti* or Dominica* orEcuador* or Egypt* or “ElSalvador*” or Eritrea* orEthiopia* or Fiji* orGabon* or Gambia* orGeorgia* or Ghana* orGrenad* or Guatemala*or Guinea* or Guyan* orHaiti* or Hondura* orHungar* or India* orIndonesia* or Iran* orIraq* or Jamaica* orJordan* or Kazakhstan*or Kenya* or Kiribati* orKorea* or Kosov* orKyrgyz* or Lao* orLeban* or Lesotho* orLiberia* or Libya* orMacedonia* orMadagascar* or Malawi*or Malaysia* or Maldiv*or Mali* or “MarshallIsland*” or Mauritania* orMauriti* or Mexic* orMoldova* or Mongolia* orMontenegr* or Morocc*or Mozambi* or Myanma*
Samoa* or “Sao Tome*”or Senegal* or Serbia* orSeychell* or “SierraLeon*” or “SolomonIsland*” or Somalia* orSudan* or “Sri Lanka*” or“St Lucia*” or “SaintLucia” or “St Vincent” or“Saint Vincent” orGrenadines or Surinam*or Swazi* or Syria* orTajikistan* or Tanzania*or Thai* or Timor* orTogo* or Tonga* orTunisia* or Turk* orTuvalu* or Uganda* orUkrain* or Uzbekistan* orVanuatu* or Venezuela*or Vietnam* or “Viet-Nam*” or “West Bank” orGaza or Yemen* orZambia* or Zimbabwe*or Russia* or Croatia* orNauru* or Yugoslavia* orUSSR or Soviet* orByelarus* or Belarus*))OR ((africa* or asia* orcaribbean or "centralamerica*" or "latinamerica*" or "southamerica*" or melanesia*or micronesia* orpolynesia*))

Supplementary Table S2. Distribution of studies reporting the prevalence or health impact of contraceptive-induced menstrual changes in low- and middle-income countries

Country	Number	Economy	Type of data
Angola	1	LMIC	Health impact
Burkina Faso	1	LIC	Health impact
Burundi	1	LIC	Health impact
Ghana	2	LMIC	Health impact
Madagascar	1	LIC	Health impact
Zimbabwe	1	LMIC	Health impact
Tanzania	1	LMIC	Health impact
Armenia	1	UMIC	Prevalence
Cote D'Ivoire	1	LMIC	Prevalence
Haiti	1	LMIC	Prevalence
Indonesia	5	UMIC	Prevalence
Iraq	4	UMIC	Prevalence
Paraguay	1	UMIC	Prevalence
Papua New Guinea	1	LMIC	Prevalence
Venezuela	2	UMIC/temporarily unclassified	Prevalence
Argentina	4	UMIC	Prevalence, Health impact
Ecuador	2	UMIC	Prevalence, Health impact
Colombia	3	UMIC	Prevalence, Health impact
Mexico	6	UMIC	Prevalence, Health impact
Peru	1	UMIC	Prevalence, Health impact
Bangladesh	6	LMIC	Prevalence, Health impact
Dominican Republic	2	UMIC	Prevalence, Health impact
Kenya	9	LMIC	Prevalence, Health impact
Uganda	10	LIC	Prevalence, Health impact
Democratic Republic of Congo	3	LIC	Prevalence, Health impact
Ethiopia	12	LMIC	Prevalence, Health impact
Guatemala	1	UMIC	Prevalence, Health impact
Jordan	3	LMIC	Prevalence, Health impact
Malawi	4	LIC	Prevalence, Health impact
Nigeria	37	LMIC	Prevalence, Health impact
Zambia	4	LMIC	Prevalence, Health impact
Senegal	4	LMIC	Prevalence, Health impact
Turkey	7	UMIC	Prevalence, Health impact
Cuba	3	UMIC	Prevalence, Health impact
Brazil	25	UMIC	Prevalence, Health impact, Treatment
Thailand	15	UMIC	Prevalence, Health impact, Treatment
South Africa	16	UMIC	Prevalence, Health impact
China	12	UMIC	Prevalence, Health impact, Treatment

Pakistan	8	LMIC	Prevalence, Health impact, Treatment
Egypt	9	LMIC	Prevalence, Health impact, Treatment
India	43	LMIC	Prevalence, Health impact, Treatment
Iran	18	LMIC	Prevalence, Health impact, Treatment
Nepal	5	LMIC	Prevalence, Health impact, Treatment
Malaysia	1	UMIC	Prevalence, Treatment

Some studies were done across multiple countries

Supplementary Table S3. Type of contraceptive used in included studies

Contraceptives	Classification	Number of studies reporting prevalence	Total number
Combined oral contraceptives (levonorgestrel plus ethinyloestradiol; ethinyloestradiol plus gestodene; medroxyprogesterone acetate plus oestradiol cypionate; Yasmin – etinyloestradiol pus drospirenone)	Combined oral contraceptives	11	31
Combined injectable contraceptive (Mesigyna - norethisterone enanthate plus oestradiol valerate; dihydroxyprogesterone acetophenide plus oestradiol enanthate; Cyclofem; norethisterone oenanthate)	Combined injectable contraceptives	13	
Combined vaginal ring (NuvaRing)	Combined vaginal ring	5	
Patch - Ethinyl oestradiol and Norelgestromin	Combined Patch	2	
Progestin-only oral contraceptive (desogestrel, lynestrenol,	Progestin-only oral contraceptives	5	53
Progestin-only injectables (Depo medroxyprogesterone acetate; Norethisterone enanthate)	Progestin-only injectable contraceptives	47	
Progesterone vaginal ring	Progesterone vaginal ring	1	
Levonogestrel implant (Jadelle/ Norplant); etonogestrel (Implanon); nesterone; Nomegestrol acetate (Uniplant)	Progestin-containing implants	69	69
Levonorgestrel IUD (Mirena;	Progestin-containing intrauterine devices	68	68
Copper IUD	Copper intrauterine devices		
Ormeloxifene (Centchroman)	Selective oestrogen receptor modulator	4	4
Unspecified/any modern contraceptive	Oral contraceptive	15	15

Supplementary Table S4. Prevalence of contraceptive-induced menstrual changes by type of contraceptive used (220 studies)

**Type of menstrual change**	**Number of studies[a]**	**Heavy menstrual bleeding**	**Breakthrough (intermenstrual) bleeding/spotting**	**Amenorrhoea**	**Frequent bleeding**	**Infrequent bleeding**	**Prolonged bleeding**	**Menstrual changes (unspecified)**
Combined contraceptives	31	1.2 – 71.7%	0 – 48.0%	0 – 23.5%	1.6 – 16.0%	0 – 19.0%	0 – 77.5%	0 – 81.3%
Progestin-only contraceptives	53	0 – 44.1%	1.8 – 58.8%	2.5 – 74.4%	7.5 – 63.7%	4.6 – 47.9%	0 – 13.4%	0 – 90%
Implants	69	0.7 – 57.6%	2.2 – 54.7%	0 – 80.0%	0 – 28.5%	3.4 – 60.9%	0 – 60.7%	2.3 – 85.9%
Intrauterine devices (hormonal and copper)	68	0 – 64.3%	0 – 38.2%	2.0 – 80.0%	2.1% [b]	19.4 - 28.0%	1.1 – 50.0%	0.5 – 94.0%
Ormeloxifene	4	-	-	4.0 – 16.0%	8%[b]	11.0 – 16.0%	-	8.0 – 40.0%
Other/unspecified contraceptives	15	5.6 – 30.0%	10.6 – 43.3%	0 – 16.7%	-	26.4% [b]	-	7.3 – 90.3%

[a]Does not add up to total number of studies due to multiple reports; [b]Value from only one study

Supplementary Table S5. Prevalence of contraceptive discontinuation due to menstrual changes

**Type of contraceptive**	**Number of studies**	**Prevalence of contraceptive discontinuation due to menstrual changes**
Combined contraceptives	12	1.0 – 71.9%
Progestin-only contraceptives	27	0.7 – 95.0%
Implants	36	3.3 – 80.7%
Intrauterine devices (hormonal and copper)	37	1.2 – 100.0%
Ormeloxifene (Centchroman)	1	30%
All/unspecified contraceptives	19	0 – 73.8%

Supplementary Table S6. Drugs reported as being used in the treatment of women with contraceptive-induced menstrual changes

Drug used for CIMC management	Dosage	Regimen	Drug classification	Type of contraceptive	Number of studies	References[a]
Celecoxib	200 mg	Once daily for 5 days	NSAID	Implant	1	Buasang 2009
Mefenamic acid	500 mg	Two or three times daily for 5 days or 7 days in two consecutive cycles or every 4 weeks	NSAID	Copper IUD, DMPA, implant	9	Upawi 2020, Casey 2014, Rajabi 2015, Phaliwong 2004, Tantiwattanakul 2004, Sothornwit 2021, Wasim 2018, Saharkhiz 2017, Sordal 2013
Ibuprofen	400 mg	Three times daily for 5 days	NSAID	Copper IUD, implant	4	Bharati 2000, Hubacher 2006, Neinavaei 2014, Casey 2014
Naproxen	252 mg or 500 mg	252 mg three times daily or 500 mg two times daily	NSAID	Copper IUD, LNG-IUS	2	Neinavaei 2014, Madden 2012
Valdecoxib	40 mg	Daily for 5 days	NSAID	DMPA	1	Nathirojanakun 2006
Indomethacin	25mg	Every 12 hours for 5 days	NSAID	Copper IUD	1	Neinavaei 2014
Doxycycline	100 mg	Twice daily for 5 days	Antibiotic	DMPA, implant,	3	Abdel-Aleem 2012, Casey 2014, Weisberg 2009
Tranexamic acid	250 mg/500 mg/1000 mg	250 mg three/four times daily; 500 mg two/three times daily; 1000 mg two times daily for 3 – 5 days	Antifibrinolytic	Copper IUD, DMPA, implant, LNG-IUS	9	Wasim 2018, Alanwar 2018, Garg 2019, Shruti 2019, Saharkhiz 2017, Sordal 2013, Senthong 2009, Phupong 2006, Lin 2007
Combined oral contraceptive	20 µg ethinyloestradiol/150 mg desogestrel; Ethinyloestradiol 30 µg /Levonorgestrel 150 µg	Two continuous cycles; 21 days during cycle or until menstrual bleeding	Progestin + oestrogen	Implant; DMPA	3	Upawi 2020, Mohebbi-Kian 2014, Sadeghi-Bazargani 2006
Oestradiol etinyl	20 µg	Once daily for 10 days	Oestrogen	Implant	2	Archer 2008, Upawi 2020
Estriol	2 mg	Once daily 3 weeks/month for 3 months	Oestrogen	DMPA	1	MalunondAli 2009
Transdermal oestradiol	0.1 mg	Continuous use	Oestrogen	LNG-IUS	1	Madden 2012

Desmopressin	300 µg (one intranasal spray in each nostril)	The morning of the first 5 days of the menstrual cycle	Antidiuretic hormone	Copper IUD	1	Mercorio 2003
Tamoxifen	10 mg	Two times daily for 10 days	Selective oestrogen receptor modulator	Implant	1	Abdel-Aleem 2005
Ulipristal acetate	5 mg	Daily for 5 days	Selective progesterone receptor modulator	LNG-IUS	1	Fava 2020
Mifepristone	25 mg/50 mg/100 mg	100mg at insertion and then at 30-day intervals for 3 months; 100 mg/day for two consecutive days every 30 days from months 2 - 7 of implant use; 50 mg once every 4 weeks; 50 mg every two weeks for 24 weeks; 25 mg mifepristone twice daily on day 1 + etinyl oestradiol 20 ug for 4 days	Antiprogesterone	DMPA, implant	5	Lal 2010, Cheng 2000, Massai 2004, Weisberg 2009, Jain 2003
Multivitamin	vitamins A (1500 µg), B1 (1500 µg), B2 (1700 µg), B3 (20000 µg), B6 (2000 µg), B9/folic acid (400 µg), B12 (6 µg), C (60000 µg), D3 or Cholecalciferol (10 µg), E (10000 µg)	Once daily for 7 days prior to initiation of combined oral contraceptives and during the 7-day pill-free interval between pill packs for five cycles	Vitamin	Combined oral contraceptive	1	Mohammad-Alizadeh-Charandabi 2015
Vitamin B1	100 mg	Daily during the second, third and fourth months following the insertion of IUD	Vitamin	Copper IUD	1	Jafari 2014
Diosmine	500 mg	Two times daily 3 weeks/month for 3 months	Dietary supplement	DMPA	1	MalunondAli 2009
Cumin	500 mg	Every 8 hours for 7 days	Dietary supplement	IUD	1	Rajabi 2015
Oral micronized flavonoids	500 mg	Every 6 hours for the first three days of menstruation	Dietary supplement	Copper IUD	1	Alanwar 2018

[a]Reference list in Supplementary file 3

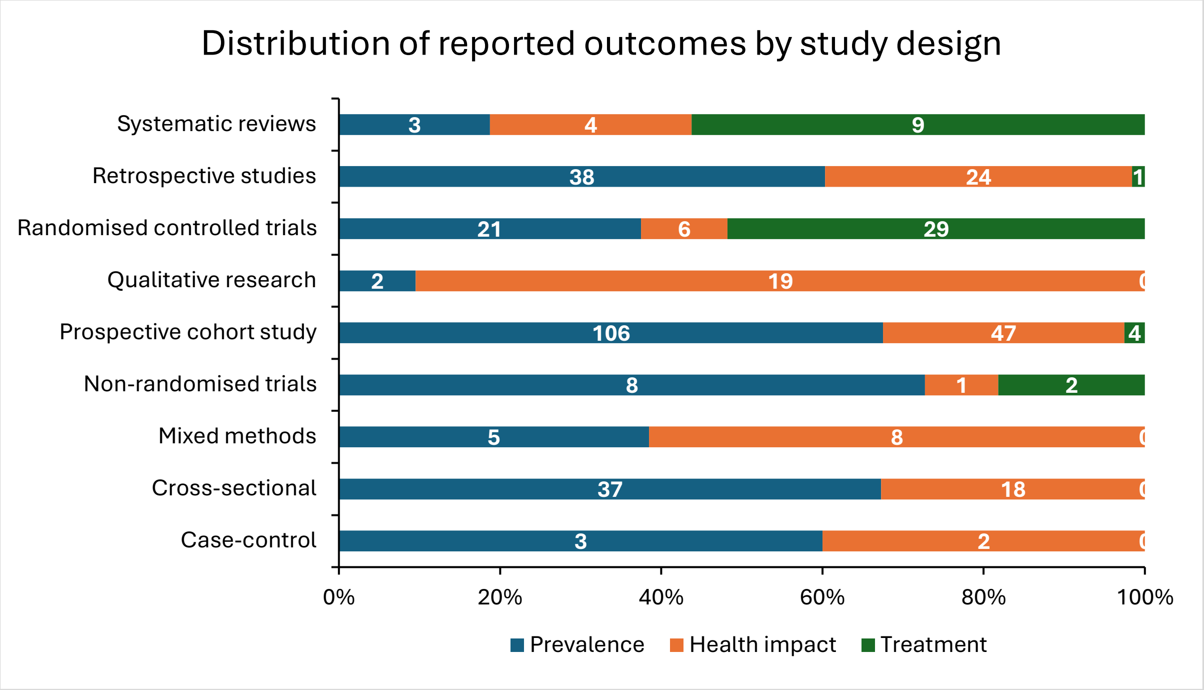

Supplementary Figure S1. Distribution of studies reporting prevalence, health impact, and treatment of CIMC by study design

a

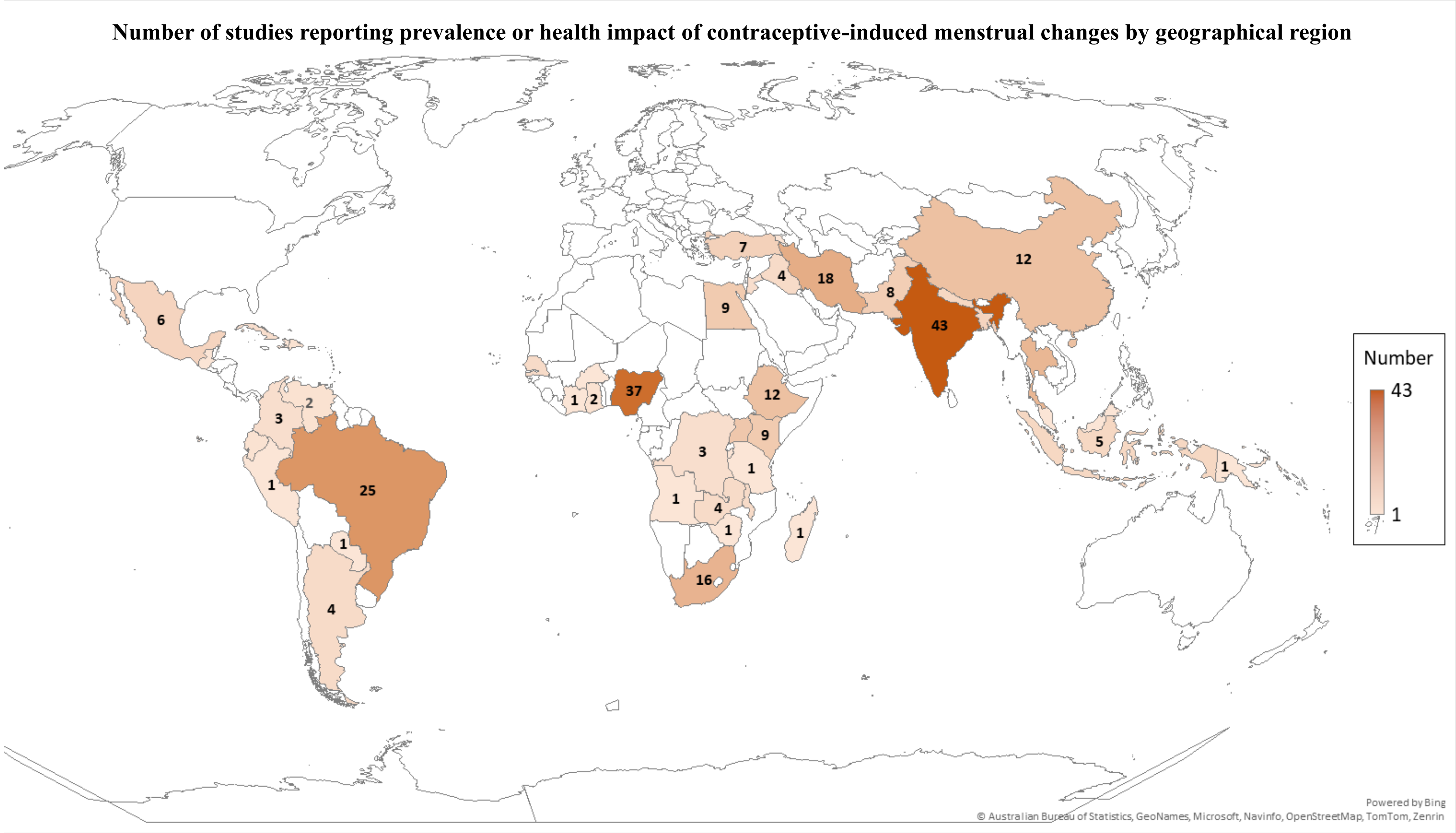
Number of studies reporting prevalence or health impact of contraceptive-induced menstrual changes by geographical region
Number
43
1
Powered by Bing
© Australian Bureau of Statistics, GeoNames, Microsoft, Navinfo, OpenStreetMap, TomTom, Zenrin

b

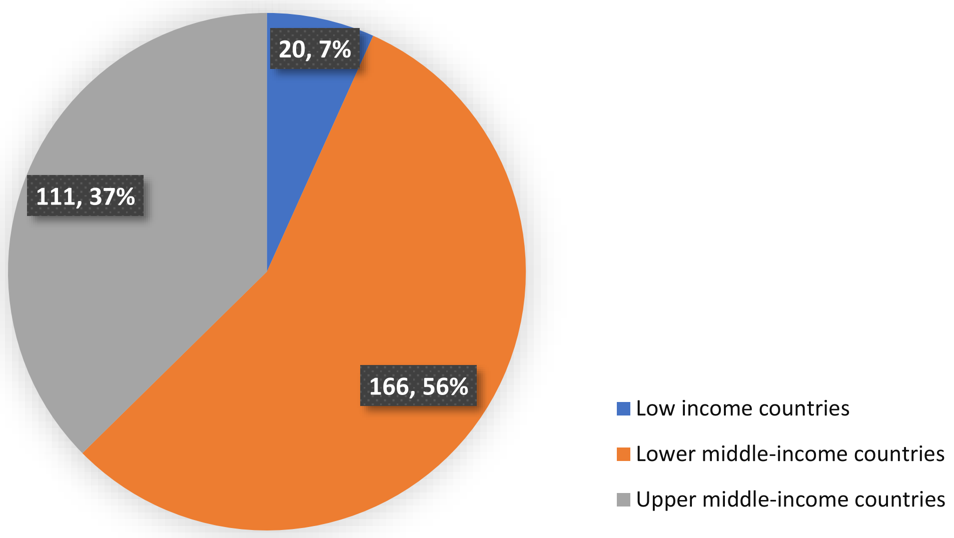

Supplementary Figure S2. Distribution of studies reporting the prevalence or health impact of contraceptive-induced menstrual changes (a) in low- and middle-income countries (b) by 2022 World Bank country classifications by income levels

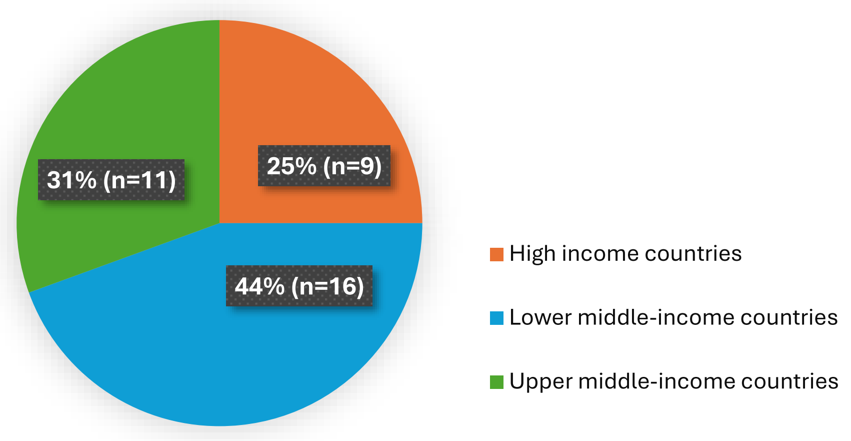

Supplementary Figure S3. Distribution of studies reporting treatment of contraceptive-induced menstrual changes by 2022 World Bank country classifications by income levels
