# Supplementary material for: Contraceptive-induced menstrual changes in low- and middle-income countries: a systematic scoping review
Source: Commun Med (Lond). 2025 Dec 17;6:43. doi: 10.1038/s43856-025-01297-5 (PMC12820136; doi:10.1038/s43856-025-01297-5)
Supplement: Supplementary file 3 — Description of Additional Supplementary files [file 43856_2025_1297_MOESM3_ESM.docx]

**Description of Additional Supplementary Files**

Supplementary Data 1- PRISMA-ScR-Checklist

Supplementary Data 2- Reference list of excluded studies and studies that could not be retrieved

Supplementary Data 3- Reference list of included studies
