# Supplementary material for: Contraceptive-induced menstrual changes in low- and middle-income countries: a systematic scoping review
Source: Commun Med (Lond). 2025 Dec 17;6:43. doi: 10.1038/s43856-025-01297-5 (PMC12820136; doi:10.1038/s43856-025-01297-5)
Supplement: Supplementary file 5 — Supplementary Data 2. [file 43856_2025_1297_MOESM5_ESM.pdf]

Supplementary Data 2. Reference list of excluded studies and studies that could not be retrieved

[1-172]

1. An Investigation of the Using of Implanon(Etonogestrel Implant) QIN Taizhou,XIE Li,QIAO Lin,et al (West China Second University Hospital,Sichuan Chengdu 610041,China) Corresponding author: XU Kehui. Journal of Practical Obstetrics and Gynecology, 2015. 31(3): p. 217-220.
2. User perspectives on new long-acting contraceptive technologies: Final Report. User perspectives on new long-acting contraceptive technologies: Final Report, 2017: p. 131-pp.
3. Abasiattai, A.M., et al., Combined oral contraceptive pills: profile of acceptors in a tertiary hospital in South-South Nigeria. Nigerian Medical Journal, 2011. 52(1): p. 19-23.
4. Abdi, F., et al., Determinants of satisfaction from depot medroxyprogesterone acetate, cyclofem, and low dose contraceptive pills: A descriptive study on health centers in Iran. Journal of Isfahan Medical School, 2012. 30(197).
5. Adanu, R.M., et al., Sexual and reproductive health in Accra, Ghana. Ghana medical journal, 2012. 46(2): p. 58-65.
6. Adeagbo, O., et al., Uptake and early removals of Implanon NXT in South Africa: Perceptions and attitudes of healthcare workers. South African medical journal = Suid-Afrikaanse tydskrif vir geneeskunde, 2017. 107(10): p. 822-826.
7. Adeoye, Y.R., et al., Determinants of Contraceptive Options among Postpartum Women Attending Selected Health Care Facilities in Nigeria: A Cross-Sectional Study. SAGE open nursing, 2024: p. 1-11.
8. Adeyemi-Fowode, O.A., et al., Levonorgestrel-Releasing Intrauterine Device Use in Female Adolescents with Heavy Menstrual Bleeding and Bleeding Disorders: Single Institution Review. Journal of Pediatric and Adolescent Gynecology, 2017. 30(4): p. 479-483.
9. Agyemang, J., et al., Contraceptive use and associated factors among sexually active female adolescents in Atwima Kwanwoma District, Ashanti region-Ghana. Pan African Medical Journal, 2019. 32: p. 182.
10. Aisien, A.O., et al., Safety, efficacy and acceptabilty of NorplantR implants in Jos, Northern Nigeria. Tropical Journal of Obstetrics and Gynaecology, 2004. 21(2): p. 95-99.
11. Al-Ghashri, F., et al., Discontinuation of hormonal contraception in Oman: prevalence and reasons. Eastern Mediterranean Health Journal, 2021. 27(10): p. 993-1000.
12. Almasi, J., T. Fel, and J. Revesz, First experiences with NuvaRing in Hungary: Investigation of reliability, acceptability and other non-contraceptive benefits, in a multicentric, open-label clinical study. Magyar Noorvosok Lapja, 2006. 69(5): p. 455-461.
13. Ammara, I., U.R. Khaliq, and B. Yusra, <The> frequency of different contraceptives uses and their side effects in clients visiting family planning center, BVH Bahawalpur. 2016. p. 75-77.
14. Anonymous, The efficacy, side-effects and continuation rates in women with symptomatic endometriosis undergoing treatment with an intra-uterine administered progestogen (levonorgestrel): A 3 year follow-up. Obstetrical and Gynecological Survey, 2005. 60(7): p. 443-445.
15. Arowojolu, A.O., I.A. Okewole, and A.O. Adekunle, Comparative evaluation of the effectiveness and safety of two regimens of levonorgestrel for emergency contraception in Nigerians. Contraception, 2002. 66(4): p. 269-273.
16. Ayele, S.G., et al., Prevalence of modern contraceptive discontinuation and associated factors among married reproductive age group women in Debre Berhan town, Ethiopia: a community-based cross-sectional study. BMJ Open, 2024. 14(4): p. e066605.
17. Bahamondes, L., et al., A 3-year multicentre randomized controlled trial of etonogestrel- and levonorgestrel-releasing contraceptive implants, with non-randomized matched copper-intrauterine device controls. Human Reproduction, 2015. 30(11): p. 2527-2538.
18. Bahamondes, L., et al., Aceitabilidade, desempenho clínico e padrão de sangramento em usuárias do sistema intrauterino liberador de Levonorgestrel (Mirena) durante três anos. Reprod. clim, 2003. 18: p. 37-40.
19. Bameka, A., et al., Early discontinuation of long-acting reversible contraceptives and associated factors among women discontinuing long-acting reversible contraceptives at national referral hospital, Kampala-Uganda; a cross-sectional study. Contraception and reproductive medicine, 2023. 8(1): p. 27.
20. Bandora, R.M., Fake-talk, side effects and the trouble with hormonal contraceptives among women in Dar es Salaam. Medicine Anthropology Theory, 2023. 10(3).

21. Baroti, E., E.N. Samani, and N. Valaei, The usage of minipill (progesterone only pill), maintenance of its consumption & related factors among lactating women in Tehran City 2005-2007. Pejouhandeh, 2008. 13(5): p. Pe439-En11.
22. Bassol, S., et al., Latin American experience with two low-dose oral contraceptives containing 30 microg ethinylestradiol/75 microg gestodene and 20 microg ethinylestradiol/150 microg desogestrel. Contraception, 2000. 62(3): p. 131-135.
23. Baum, A., et al., Factors Predicting Removals of the Levonorgestrel-Releasing Intrauterine System in an Adolescent Cohort. Journal of Pediatric and Adolescent Gynecology, 2024. 37(2): p. 171 EP-176.
24. Bhatia, P., Study on emergency contraception with Cu T 200 B and 0.75 mg levonorgestrel (2 doses) using cafeteria approach. Journal of Obstetrics and Gynecology of India, 2011. 61(3): p. 317-322.
25. Blumenthal, P.D., L. Dawson, and R. Hurskainen, Cost-effectiveness and quality of life associated with heavy menstrual bleeding among women using the levonorgestrel-releasing intrauterine system. International Journal of Gynecology and Obstetrics, 2011. 112(3): p. 171-178.
26. Brandão, E.R., Desafios da contracepção juvenil: interseções entre gênero, sexualidade e saúde. Ciênc. Saúde Colet. (Impr.), 2009. 14(4): p. 1063-1071.
27. Brasil. Ministério da Saúde. Comissão Nacional de Incorporação de Tecnologias do, S.U.S., Sistema intrauterino liberador de levonorgestrel 52 mg para anticoncepção em mulheres de 15 a 19 anos de idade. 2016.
28. Brunie, A., et al., What are the prospects for the hormonal IUD in the public sector? A mixed-method study of the user population in Zambia. BMC Women's Health, 2022. 22(1): p. 1-13.
29. Brunie, A., et al., Factors affecting uptake of the levonorgestrelreleasing intrauterine device: A mixedmethod study of social franchise clients in Nigeria. PLoS One, 2021. 16(9 September): p. e0257769.
30. Burke, H.M., et al., A Field Test of the NORMAL Job Aid With Community Health Workers in Kenya to Address Contraceptive-Induced Menstrual Changes. Global health, science and practice, 2023. 11(1).
31. Caiyan, W., et al., Efficacy and safety of a combined oral contraceptive containing drospirenone 3 mg and ethinylestradiol 20 microg in a 24+4-day regimen in China. Zhonghua fu chan ke za zhi, 2014. 49(5): p. 355-359.
32. Carvajal, A., et al., Emergency contraceptive pill safety profile. Comparison of the results of a follow-up study to those coming from spontaneous reporting. Pharmacoepidemiology and Drug Safety, 2015. 24(1): p. 93-97.
33. Chen, Q.J., et al., Efficacy and safety of a levonorgestrel enteric-coated tablet as an over-the-counter drug for emergency contraception: A Phase IV clinical trial. Human Reproduction, 2011. 26(9): p. 2316-2321.
34. Choudhury, N., et al., "Even things they won't share with their sisters-in-law" - Assessing an integrated Community Health Worker intervention on person-centered postpartum contraception in rural Nepal. medRxiv, 2024.
35. Clark, L.R., et al., Menstrual irregularity from hormonal contraception: a cause of reproductive health concerns in minority adolescent young women. Contraception, 2006. 74(3): p. 214-9.
36. Cremer, M., et al., Depot-medroxyprogesterone acetate contraception use among salvadoran women: An in-depth analysis of attitudes and experiences. Journal of Women's Health, 2011. 20(11): p. 1751-1756.
37. D'Arcangues, C., et al., Women's views and experiences of their vaginal bleeding patterns: An international perspective from Norplant users. European Journal of Contraception and Reproductive Health Care, 2011. 16(1): p. 9-17.
38. Darney, P., et al., Safety and efficacy of a single-rod etonogestrel implant (Implanon): results from 11 international clinical trials. Fertility and sterility, 2009. 91(5): p. 1646-1653.
39. Das, S., et al., Combined contraceptive vaginal ring- its acceptability in Indian women. Indian Journal of Public Health Research and Development, 2016. 7(1): p. 57-63.
40. Deokar, A.M., W. Jackson, and H.A. Omar, Menstrual bleeding patterns in adolescents using etonogestrel (ENG) implant. International Journal of Adolescent Medicine and Health, 2011. 23(1): p. 75-77.
41. Dewan, R. and P. Rani, Clinical outcome of post-abortion intrauterine contraceptive device insertion. 2020. 9.
42. Diaz, J., et al., Acceptability and performance of the levonorgestrel-releasing intrauterine system (Mirena()) in Campinas, Brazil. Contraception, 2000. 62(2): p. 59-61.
43. Do Ngoc, T., Assessment of side - effects of DMPA. Journal of Medical Research, 2003: p. 135-140.
44. Draper, B.H., et al., Depot medroxyprogesterone versus norethisterone oenanthate for long-acting progestogenic contraception. Cochrane database of systematic reviews (Online), 2006. 3: p. CD005214.

45. Elsedeek, M.S. and M.S.E. Elsedeek, Puerperal and menstrual bleeding patterns with different types of contraceptive device fitted during elective cesarean delivery. International Journal of Gynecology & Obstetrics, 2012. 116(1): p. 31-34.
46. Endrikat, J.S., et al., Bleeding pattern, tolerance and patient satisfaction with a drospirenone-containing oral contraceptive evaluated in 3488 women in Europe, the Middle East and Canada. Contraception, 2009. 79(6): p. 428-32.
47. Enyindah, C.E., J.D. Ojule, and G. Bassey, Contraception with intrauterine contraceptive device (IUCD) in port harcourt, south-south Nigeria. Journal of Medicine and Biomedical Research, 2012. 11(1): p. 35-45.
48. F, R., et al., Continuation rate of contraceptive methods and causes of their discontinuation in Zahedan. 2002. p. 41-49.
49. Fan, G., et al., A single-arm phase III study exploring the efficacy and safety of LNG-IUS 8, a low-dose levonorgestrel intrauterine contraceptive system (total content 13.5 mg), in an Asia-Pacific population. Contraception, 2017. 95(4): p. 371-377.
50. Feng, Z. and L. Feng, Investigation on side effects after placing IUD in bearing-age women of Longgang district of Shenzhen. Maternal and Child Health Care of China, 2010. 25(14): p. 1951-1953.
51. Fraser, I.S., et al., A detailed analysis of menstrual blood loss in women using Norplant (R) and Nestorone (R) progestogen-only contraceptive implants or vaginal rings. Contraception, 2000. 61(4): p. 241-251.
52. Gainer, E., et al., Menstrual bleeding patterns following levonorgestrel emergency contraception. Contraception, 2006. 74(2): p. 118-124.
53. Glasier, A.F., et al., Amenorrhea associated with contraception - An international study on acceptability. Contraception, 2003. 67(1): p. 1-8.
54. Gomez, L.M., et al., Efectos adversos y motivos de retiro de implante subdérmico Jadelle® en usuarias de Policlínica de salud Sexual y Reproductiva del Hospital de Clínicas en período junio 2015- diciembre 2017. Rev. chil. obstet. ginecol. (En línea), 2021. 86(1): p. 68-75.
55. Gómez-Sánchez, P.I. and Y. Pardo, Revista Colombiana de Obstetricia y Ginecologia, 2010. 61(1): p. 34-41.
56. Grimes, D.A., et al., Cochrane systematic reviews of IUD trials: lessons learned. Contraception, 2007. 75(6 SUPPL.): p. S55-S59.
57. Grimes, D.A., et al., Progestin-only pills for contraception. Cochrane Database of Systematic Reviews, 2013. 2013(11): p. CD007541.
58. Grunloh, D.S., et al., Characteristics associated with discontinuation of long-acting reversible contraception within the first 6 months of use. Obstetrics and gynecology, 2013. 122(6): p. 1214-21.
59. Gunardi, E.R., R.A. Putri, and Y. Pasidri, A prospective study of effectivity, expulsion, and acceptability of post-placental iud cu t380a insertion using clamp in a tertiary hospital. Journal of SAFOG, 2021. 13(2): p. 92-96.
60. Haddad, L., et al., Contraceptive discontinuation and switching among couples receiving integrated HIV and family planning services in Lusaka, Zambia. AIDS (London, England), 2013. 27 Suppl 1: p. S93-103.
61. Halpern, V., et al., Strategies to improve adherence and acceptability of hormonal methods of contraception. Cochrane Database of Systematic Reviews, 2013(10): p. N.PAG-N.PAG.
62. Halpern, V., E.G. Raymond, and L.M. Lopez, Repeated use of pre- and postcoital hormonal contraception for prevention of pregnancy. Cochrane Database of Systematic Reviews, 2014(9): p. N.PAG-N.PAG.
63. Hapangama, D.K., et al., Feasibility of administering mifepristone as a once a month contraceptive pill. Human Reproduction, 2001. 16(6): p. 1145-1150.
64. Hertzen, H.v., Efficacy and side effects of immediate postcoital levonorgestrel used repeatedly for contraception. Contraception (Stoneham), 2000. 61(5): p. 303-308.
65. Hlongwa, M., C. Mutambo, and K. Hlongwana, in fact, that's when i stopped using contraception': A qualitative study exploring women's experiences of using contraceptive methods in KwaZulu-Natal, South Africa. BMJ Open, 2023. 13(4): p. A342.
66. Holanda, A.A.R.d., et al., Controvérsias acerca do dispositivo intrauterino: uma revisão. Femina, 2013. 41(3).
67. Hou, S.P., et al., Acceptance and Continuation of Contraceptive Methods Immediate Postabortion. Gynecologic and Obstetric Investigation, 2017. 82(1): p. 86-95.

68. Hubacher, D., P.L. Chen, and S. Park, Side effects from the copper IUD: do they decrease over time? Contraception, 2009. 79(5): p. 356-362.
69. Jaisamrarn, U., D. Reinprayoon, and P. Virutamasen, Clinical study of a monophasic pill containing 20 microg ethinylestradiol and 150 microg desogestrel in Thai women. 2001.
70. Jensen, J., et al., Bleeding patterns with the levonorgestrel-releasing intrauterine system when used for heavy menstrual bleeding in women without structural pelvic pathology: A pooled analysis of randomized controlled studies. Contraception, 2013. 87(1): p. 107-112.
71. Jindabanjerd, K. and S. Taneepanichskul, The use of levonorgestrel - IUD in the treatment of uterine myoma in Thai women. Journal of the Medical Association of Thailand = Chotmaihet thangphaet, 2006. 89 Suppl 4: p. S147-151.
72. Kaewrudee, S. and S. Taneepanichskul, Norplant users with irregular bleeding. Ultrasonographic assessment and evaluation of serum concentrations of estradiol and progesterone. The Journal of reproductive medicine, 2000. 45(12): p. 983-6.
73. Kanakannavar, S.S. and A.R. S, Clinicomicrobiological study of the removed intrauterine device. 2019. 8.
74. Kangale, E., et al., Study Of Acceptance, Safety And Expulsion Of Post-placental And Intra-caesarean Intrauterine Contraceptive Device Among Postpartum Mothers In Tertiary Care Institute. Research Journal of Pharmaceutical, Biological and Chemical Sciences, 2023. 14(5): p. 228 EP-233.
75. Kariman, N., Z.P. Sheykhi, and H.M. Alavi, Comparison of effects of injectable contraceptives (cyclofem and depot medroxyprogesterone acetate) on short time side effects, acceptability and continuation rates. Iranian Journal of Obstetrics, Gynecology and Infertility, 2014. 17(91): p. 12-20.
76. Kelekci, S., K.H. Kelekci, and B. Yilmaz, Effects of levonorgestrel-releasing intrauterine system and T380A intrauterine copper device on dysmenorrhea and days of bleeding in women with and without adenomyosis. Contraception, 2012. 86(5): p. 458-463.
77. Khamees, R.E., et al., Effects of the levonorgestrel-releasing intrauterine system versus the copper intrauterine device on uterine artery Doppler indices. European Journal of Contraception and Reproductive Health Care, 2022. 27(1): p. 23-27.
78. Khan, S., H. Zareen, and S. Shahzad, Factors and Determinants of Unmet Needs: Identifying Association Between Variables and Unmet Needs Among Married Women of Child Bearing Age in Lahore, Pakistan. 2018. 4.
79. Kirkham, Y.A., et al., Trends in menstrual concerns and suppression in adolescents with developmental disabilities. Journal of Adolescent Health, 2013. 53(3): p. 407-412.
80. Kokonya, D.A., et al., Experience with IUCD insertion outside of menses in Kenya. East African Medical Journal, 2000. 77(7): p. 369-373.
81. Kriplani, A., et al., A 1-year comparison of TCu380Ag versus TCu380A intrauterine contraceptive devices in India. International Journal of Gynecology & Obstetrics, 2019. 145(3): p. 268-277.
82. Kumar, S., et al., Attitude of women towards family planning methods and its use--study from a slum of Delhi. Kathmandu University medical journal (KUMJ), 2005. 3(3): p. 259-262.
83. Kupoluyi, J.A., et al., Prevalence and associated factors of modern contraceptive discontinuation among sexually active married women in Nigeria. Contraception and reproductive medicine, 2023. 8(1): p. 8.
84. L'Engle, K.L., L. Hinson, and D. Chin-Quee, "I love my ECPs": Challenges to bridging emergency contraceptive users to more effective contraceptive methods in Ghana. Journal of Family Planning and Reproductive Health Care, 2011. 37(3): p. 146-151.
85. Lateef, M.R., The duration and the severity of menstrual bleeding in iraqi women using intrauterine contraceptive device (IUD): Cross sectional study. Indian Journal of Public Health Research and Development, 2020. 11(4): p. 1039-1042.
86. Lazorwitz, A., et al., Relationship between Etonogestrel Concentrations and Bleeding Patterns in Contraceptive Implant Users. Obstetrics and gynecology, 2019. 134(4): p. 807-813.
87. Leal F, I., et al., Patrón de sangrado uterino en adolescentes usuarias de implante anticonceptivo subdérmico de etonogestrel. Rev. chil. obstet. ginecol, 2016. 81(6): p. 489-495.
88. Lerkiatbundit, S. and W. Reanmongkol, Use of 0.75 mg Levonorgestrel for postcoital contraception in Thailand. Journal of Clinical Pharmacy and Therapeutics, 2000. 25(3): p. 185-190.
89. Lunde, B., et al., "Just Wear Dark Underpants Mainly": Learning from Adolescents' and Young Adults' Experiences with Early Discontinuation of the Contraceptive Implant. Journal of Pediatric and Adolescent Gynecology, 2017. 30(3): p. 395-399.

90. M, S., et al., [Investigating prevalence rate and effective parameters on switching contraceptive methods]. 2014. p. 1-10.
91. Mackenzie, A.C.L., et al., Women's Perspectives on Contraceptive-Induced Amenorrhea in Burkina Faso and Uganda. International Perspectives on Sexual & Reproductive Health, 2020. 46(1): p. 247-262.
92. Mansour, D., et al., The management of unacceptable bleeding patterns in etonogestrel-releasing contraceptive implant users. Contraception, 2011. 83(3): p. 202-210.
93. Mansour, D., et al., Pooled analysis of two randomized, open-label studies comparing the effects of nomegestrol acetate/17β-estradiol and drospirenone/ethinyl estradiol on bleeding patterns in healthy women. Contraception, 2017. 95(4): p. 390-397.
94. Marvi, K. and N. Howard, Objects of temporary contraception: An exploratory study of women's perspectives in Karachi, Pakistan. BMJ Open, 2013. 3(8): p. e003279.
95. McKinney, J.R., et al., Factors influencing use of family planning services among HIV-positive women in the PMTCT program at Clinica de Familia La Romana in the Dominican Republic. Sexuality Research and Social Policy, 2013. 10(3): p. 200-207.
96. McKinney, J.R., et al., Factors influencing use of family planning services among HIV-positive women in the PMTCT program at Clinica de Familia La Romana in the Dominican Republic. [References]. 2013: Sexuality Research & Social Policy: A Journal of the NSRC. Vol.10(3), 2013, pp. 200-207.
97. Mgobhozi, L.N., P.N. Mbeje, and G.G. McHunu, Women's experiences on the use of Implanon as a contraceptive method in a selected primary healthcare facility in KwaZulu-Natal. Curationis, 2021. 44(1): p. 1-9.
98. Mihretie, G.S., et al., Factors associated with discontinuation among long-acting reversible contraceptive users: a multisite prospective cohort study in urban public health facilities in Ethiopia. BMJ Open, 2022. 12(8): p. e059372.
99. Mihretie, G.S., et al., An Interpretative Study of LARCs Discontinuation in Ethiopia: The Experiences of Women Accessing Contraceptives in Selected Public Health Facilities. OPEN ACCESS JOURNAL OF CONTRACEPTION, 2023. 14: p. 41-51.
100. Milliren, C.E., et al., Contraceptive Implant-Associated Bleeding in Adolescent/Young Adult Clinical Practice: Associated Factors, Management, and Rates of Discontinuation. Journal of Adolescent Health, 2023. 72(4): p. 583-590.
101. Miranda, L., et al., Acceptance of progestogen-only contraceptives by indigenous women from south Mexico. International Journal of Gynecology and Obstetrics, 2016. 132(2): p. 236.
102. Miranda, L., et al., Continuation rates of the 52-mg levonorgestrel-releasing intrauterine system according to the primary reason for its use. Rev. bras. ginecol. obstet, 2021. 43(4): p. 291-296.
103. Modesto, W., M.V. Bahamondes, and L. Bahamondes, A randomized clinical trial of the effect of intensive versus non-intensive counselling on discontinuation rates due to bleeding disturbances of three long-Acting reversible contraceptives. Human Reproduction, 2014. 29(7): p. 1393-1399.
104. Modesto, W., M.V. Bahamondes, and L. Bahamondes, A randomized clinical trial of the effect of intensive versus non-intensive counseling on discontinuation rates due to bleeding disturbances of three long-acting reversible contraceptives. Obstetrical and Gynecological Survey, 2015. 70(1): p. 24-26.
105. Mohamad M, W., <The> effects of levonorgestrel releasing intrauterine device on menstrual bleeding pattern, endometrial thickness and uterine vasculature. 2006. p. 41-45.
106. Mohamad, W., et al., Intrauterine contraceptive device position: a possible cause for intrauterine contraceptive device associated menorrhagia. 2007. p. 323-326.
107. Mohammad, F., Intrauterine Device Insertion at Cesarean Section. 2004. p. 65-70.
108. Moradan, S., R. Ghorbani, and S. Baghani, Incidence of abnormal uterine bleeding in individuals who used hormonal contraceptive methods and referred to Semnan health centers (2006-2007). Koomesh, 2009. 10(3): p. 219.
109. Mumah, J.N., et al., Contraceptive Adoption, Discontinuation, and Switching among Postpartum Women in Nairobi's Urban Slums. Studies in Family Planning, 2015. 46(4): p. 369-386.
110. Mumah, J.N., et al., Contraceptive adoption, discontinuation, and switching among postpartum women in Nairobi's urban slums. [References]. 2015: Studies in Family Planning. Vol.46(4), 2015, pp. 369-386.
111. Mutihir, J.T., T. Iranloye, and P.F.K. Uduagbamen, Profile of clients requesting for removal of intrauterine devices in Jos, Nigeria. Annals of African Medicine, 2006. 5(2): p. 89-92.
112. Namir G, A.T., A.H. Abdul Hussein, and B. Najla N, Determinants of intrauterine device discontinuation among women attending some family planning clinics in Baghdad. 2004. p. 91-95.

113. Nanda, K., et al., Continuous compared with cyclic use of oral contraceptive pills in the Dominican Republic: a randomized controlled trial. Obstetrics & Gynecology, 2014. 123(5): p. 1012-1022.
114. Narvekar, N., et al., Mifepristone-induced amenorrhoea is associated with an increase in microvessel density and glucocorticoid receptor and a decrease in stromal vascular endothelial growth factor. Human Reproduction, 2006. 21(9): p. 2312-2318.
115. Nasrin, B., et al., [Comparison of the effect of TCU-380A and TCU safe-300 on menstrual blood loss]. 2006. p. 33-40.
116. Niño-Avendaño, C.A., L.J. Vargas-Rodríguez, and N.M. González-Jiménez, Abandono, cambio o falla de los anticonceptivos hormonales en población universitaria. Ginecol. obstet. Méx, 2019. 87(8): p. 499-505.
117. Oddsson, K., et al., Superior cycle control with a contraceptive vaginal ring compared with an oral contraceptive containing 30 mug ethinylestradiol and 150 mug levonorgestrel: A randomized trial. Human Reproduction, 2005. 20(2): p. 557-562.
118. Okunlola, M.A., et al., Discontinuation pattern among IUCD users at the family planning clinic, University College Hospital, Ibadan. Journal of Obstetrics and Gynaecology, 2006. 26(2): p. 152-156.
119. Ortayli, N., et al., Immediate postabortal contraception with the levonorgestrel intrauterine device, Norplant, and traditional methods. Contraception, 2001. 63(6): p. 309-14.
120. Parsey, K.S. and A. Pong, An open-label, multicenter study to evaluate yasmin, a low-dose combination oral contraceptive containing drospirenone, a new progestogen. Contraception, 2000. 61(2): p. 105-111.
121. Pham Ba, N., Study on oral contraceptive pill use in Hai Duong and Da Nang. Journal of Vietnamese Medicine, 2004: p. 50-55.
122. Pires, M.L.L., et al., Indications and reasons for discontinuing the levonorgestrel-releasing intrauterine system (LNG-IUS). Revista Brasileira de Saude Materno Infantil, 2020. 20(2): p. 479-484.
123. Polis, C.B., R. Hussain, and A. Berry, There might be blood: a scoping review on women's responses to contraceptive-induced menstrual bleeding changes. Reproductive health, 2018. 15(1): p. N.PAG-N.PAG.
124. Prilepskaya, V.N. and E.N. Andreeva, SATISFACTION OF YOUNG WOMEN WITH ESTRADIOL VALERATE/DIENOGEST IN REAL CLINICAL PRACTICE IN RUSSIA: RESULTS OF PROSPECTIVE MULTICENTER OBSERVATIONAL STUDY Q-SWAN. Akusherstvo i Ginekologiya (Russian Federation), 2024. 2024(3): p. 108 EP-117.
125. Qian, C.F., et al., [Efficacy and safety of low-dose levonorgestrel-releasing intrauterine system in Chinese women: a multicenter, single-arm, open labeled interventional trial]. Zhonghua fu chan ke za zhi, 2018. 53(6): p. 409-413.
126. Rademacher, K.H., et al., Provision of the levonorgestrel intrauterine system in Nigeria: Provider perspectives and service delivery costs. Gates Open Research, 2020. 4: p. 119.
127. Radesic, B. and A. Sharma, Levonorgestrel-releasing intrauterine system for treating menstrual disorders: A patient satisfaction questionnaire. Australian and New Zealand Journal of Obstetrics and Gynaecology, 2004. 44(3): p. 247-251.
128. Rana, M., P. Saxena, and N. Firdous, Comparison of levonorgestrel and copper releasing intrauterine contraceptive device on body iron stores and menstrual bleeding patterns: Experience on Indian women. European review for medical and pharmacological sciences, 2012. 16(2): p. 230-234.
129. Raymond, E.G., et al., Bleeding patterns after use of levonorgestrel emergency contraceptive pills. Contraception, 2006. 73(4): p. 376-381.
130. Ren, J.F., et al., Study on clinical efficacy and mechanism of xiaoyan zhixue capsule in treating menorrhagia caused by intrauterine device. Zhongguo Zhong xi yi jie he za zhi Zhongguo Zhongxiyi jiehe zazhi = Chinese journal of integrated traditional and Western medicine / Zhongguo Zhong xi yi jie he xue hui, Zhongguo Zhong yi yan jiu yuan zhu ban, 2004. 24(7): p. 605-609.
131. Rezk, M., et al., Effects of a levonorgestrel intrauterine system versus a copper intrauterine device on menstrual changes and uterine artery Doppler. International Journal of Gynecology and Obstetrics, 2019. 145(1): p. 18-22.
132. Rivera, R. and W. Rountree, Characteristics of menstrual problems associated with Norplant discontinuation: Results of a multinational study. Contraception, 2003. 67(5): p. 373-377.
133. Rowe, P., et al., Safety and efficacy in parous women of a 52-mg levonorgestrel-medicated intrauterine device: a 7-year randomized comparative study with the TCu380A. Contraception, 2016. 93(6): p. 498-506.
134. S, Z., et al., Evaluation of side effects of low dose contraceptive pills administerd by the vaginal route. 2002. p. 67-70.
135. Sadiq, R. and S.I. Rao, Frequency of menstrual disorders with depot medroxy progesterone acetate (DMPA) as contraceptive. Medical Forum Monthly, 2008. 19(2): p. 22-28.

136. Saleem, S., et al., Emergency contraception. Medical Forum Monthly, 2002. 13(11): p. 27-30.
137. Salhan, S. and V. Tripathi, Factors influencing discontinuation of intrauterine contraceptive devices: An assessment in the Indian context. European Journal of Contraception and Reproductive Health Care, 2004. 9(4): p. 245-259.
138. Sapna, S.P., K. Abdul Rashid, and K.A. Narayan, Unmet Needs for Contraception in Married Women in a Tribal Area of India. Malaysian Journal of Public Health Medicine, 2010: p. 44-51.
139. Sato, R., et al., Why do women discontinue contraception and what are the post-discontinuation outcomes? Evidence from the Arusha Region, Tanzania. Sexual and Reproductive Health Matters, 2020. 28(1): p. 1723321.
140. Schrager, S., K. Fox, and R. Lee, Abnormal Uterine Bleeding Associated With Hormonal Contraception. American Family Physician, 2024. 109(2): p. 161 EP-166.
141. Schwartz, B.I., M. Alexander, and L.L. Breech, Levonorgestrel Intrauterine Device Use for Medical Indications in Nulliparous Adolescents and Young Adults. Journal of Adolescent Health, 2021. 68(2): p. 357-363.
142. Shahnazi, M., et al., Comparison of vaginal bleeding in Multiload CU 375 and Copper T 380A intrauterine device users. Iranian Journal of Obstetrics, Gynecology and Infertility, 2013. 16(47/48): p. 13-21.
143. Shams, T.M., et al., Outcomes of etonogestrel subdermal contraceptive implants A single center cross-sectional study. Saudi Medical Journal, 2024. 45(3): p. 261
EP-266.
144. Shea, A.A., et al., A Bother or a Benefit? How Contraceptive Users Balance the Trade-Offs Between Preferred Menstrual Bleeding Patterns and Preferred Contraceptive Methods in India, South Africa, and the United States. Women's Reproductive Health, 2024. 11(2): p. 343-382.
145. Shuzhi, L.I., et al., A randomized clinical trial of intrauterine device MYCu. Chinese Journal of General Practitioners, 2010(6): p. 760-764.
146. Simbar, M., et al., A comparative study of Cyclofem and depot medroxyprogesterone acetate (DMPA) effects on endometrial vasculature. Journal of Family Planning and Reproductive Health Care, 2007. 33(4): p. 271-276.
147. Singata-Madliki, M., et al., Effects of injectable contraception with depot medroxyprogesterone acetate or norethisterone enanthate on estradiol levels and menstrual, psychological and behavioral measures relevant to HIV risk: The WHICH randomized trial. PLoS One, 2024. 19(3 March): p. 1
EP-19.
148. Staveteig, S., Understanding unmet need in Ghana: results from a follow-up study to the 2014 Ghana demographic and health survey. DHS Qualitative Research Studies, 2016(20): p. ix-pp.
149. Stovall, D.W., et al., Satisfaction and continuation with LNG-IUS 12: findings from the real-world kyleena satisfaction study. European Journal of Contraception and Reproductive Health Care, 2021. 26(6): p. 462-472.
150. Subakir, S.B., et al., Oxidative stress, vitamin E and progestin breakthrough bleeding. Human Reproduction, 2000. 15(SUPPL. 3): p. 18-23.
151. Sun, X., et al., Safety and Efficacy of Combined Oral Contraceptive Ethinyl Estradiol/Drospirenone (YAZ) in Chinese Women: A Single-Arm, Open-Label, Multicenter, Post-Authorization Study. Advances in Therapy, 2020. 37(2): p. 906-917.
152. T, S., et al., [Comparison of two commonly used IUDs in pain and menstrual bleeding]. 2008. p. 23-28.
153. Tantbirojn, P. and S. Taneepanichskul, Clinical comparative study of oral contraceptives containing 30 µg ethinylestradiol/150 µg levonorgestrel, and 35 µg ethinylestradiol/250 µg norgestimate in Thai women. Contraception, 2002. 66(6): p. 401-405.
154. Tripathi, V., D. Nandan, and S. Salhan, Determinants of early discontinuation of IUCD use in rural northern district of India: A multivariate analysis and its validation. Journal of biosocial science, 2005. 37(3): p. 319-332.
155. Turok, D.K., et al., Efficacy, Safety, and Tolerability of a New Low-Dose Copper and Nitinol Intrauterine Device Phase 2 Data to 36 Months. Obstetrics and gynecology, 2020. 135(4): p. 840-847.
156. Utaile, M.M., et al., A qualitative study on reasons for early removal of Implanon among users in Arba Minch town, Gamo Goffa zone, South Ethiopia: a phenomenological approach. BMC Women's Health, 2020. 20(1): p. 1-7.
157. Wado, Y.D., et al., Women's beliefs about methods and contraceptive discontinuation: Results from a prospective study from Nairobi and Homa Bay counties in Kenya. Frontiers in global women's health, 2023. 4: p. 1034634.

158. Wafaa A, R. and A.A. Shadia, Knowledge and practices of Saudi women regarding family planning methods. 2007. p. 147-156.
159. Wang, C., et al., Efficacy and safety of a combined oral contraceptive containing drospirenone 3 mg and ethinylestradiol 20 mug in a 24 + 4-day regimen in China. Chinese Journal of Obstetrics and Gynecology, 2014. 49(5): p. 355-359.
160. Wang, H., L. Xin, and G. Gong, Comparative Study of Clinical Effect of MCuII IUD,MCu IUD and TCu220C IUD. Journal of Practical Obstetrics and Gynecology, 2012. 28(10): p. 841-844.
161. Webb, A., et al., Effect of hormonal emergency contraception on bleeding patterns. Contraception, 2004. 69(2): p. 133-135.
162. Wiegratz, I., et al., Effect of a low-dose contraceptive patch on efficacy, bleeding pattern, and safety: a 1-year, multicenter, open-label, uncontrolled study. Reproductive sciences (Thousand Oaks, Calif.), 2014. 21(12): p. 1518-25.
163. Wildemeersch, D., Intrauterine contraceptives that do not fit well contribute to early discontinuation. European Journal of Contraception and Reproductive Health Care, 2011. 16(2): p. 135-141.
164. Wonglikhitpanya, N. and S. Taneepanichskul, Effects of biphasic oral contraceptives containing desogestrel (Oilezz (R)) on cycle control facial acne and seborrhea in healthy Thai women. Journal of the Medical Association of Thailand, 2006. 89(6): p. 755-760.
165. Xie, W., et al., Gongxuening capsule for abnormal uterine bleeding after using intrauterine device. Progress in Modern Biomedicine, 2011. 11(12): p. 2314-2316.
166. Yang, X., et al., The application of Anhuantang in the treatment of menstrual disorder after the intrauterine device (IUD) was fitted. Chinese Journal of Information on Traditional Chinese Medicine, 2006. 13(3): p. 63-64.
167. Yu, Q., et al., Contraceptive efficacy and safety of estradiol valerate/dienogest in a healthy female population: A multicenter, open-label, uncontrolled phase iii study. International journal of women's health, 2018. 10: p. 257-266.
168. Zahra Pahlavani, S., et al., [<The> comparison effect of cyclofem and depo - provera on menstrual bleeding patterns in women of referred to health centers depends on the Zahedan University of Medical Sciences in 2009-2010]. 2011. p. 256-261.
169. Zahradnik, H.P., A. Hanjalic-Beck, and A. Schilling Redlich, Beneficios no anticonceptivos de 0, 02 mg de etinilestradiol/2 mg de acetato de clormadinona administrados en un régimen de 24+4 días. Rev. chil. obstet. ginecol, 2011. 76(5): p. 344-353.
170. Zhang, L., Z. Xi, and J. Song, Analysis on adverse reactions and influencing factors of intrauterine device in urban and rural women of childbearing age. Maternal and Child Health Care of China, 2013. 28(3): p. 479-481.
171. Zulu, B., P. Maharaj, and S. Dunn, A qualitative study on contraceptive use among young female university students: what still matters? African Population Studies, 2023. 36(1).
172. رضایی, ن.ا., et al., تأثير قرص هاي خوراکی پیشگیري از بارداري بر کیفیت زندگی زنان مراجعه کننده به مراکز بهداشتی و درمانی ارومیه در سال 1392. Journal of Urmia Nursing & Midwifery Faculty, 2015. 13(5): p. 425-434.
