# Supplementary material for: Contraceptive-induced menstrual changes in low- and middle-income countries: a systematic scoping review
Source: Commun Med (Lond). 2025 Dec 17;6:43. doi: 10.1038/s43856-025-01297-5 (PMC12820136; doi:10.1038/s43856-025-01297-5)
Supplement: Supplementary file 6 — Supplementary Data 3 [file 43856_2025_1297_MOESM6_ESM.pdf]

Supplementary Data 3. Reference list of included studies

[[1-321]

1. Aamir, F., A. Mahesh, and S.A. Karim, *To Evaluate the Compliance of Postpartum Intrauterine Contraceptive Device at Jinnah Medical College Hospital.* ANNALS ABBASI SHAHEED HOSPITAL & KARACHI MEDICAL & DENTAL COLLEGE, 2018. **23**(2): p. 79-85.
2. Abasiattai, A.M., E.A. Bassey, and E.J. Udoma, *Profile of intrauterine contraceptive device acceptors at the University of Uyo Teaching Hospital, Uyo, Nigeria.* Annals of African Medicine, 2008. **7**(1): p. 1-5.
3. Abasiattai, A.M., E.J. Udoma, and E. Ukeme, *Depot medroxyprogesterone injectable contraception at the University of Uyo Teaching Hospital, Uyo.* Annals of African Medicine, 2010. **9**(2): p. 81-85.
4. Abdel-Aleem, H., et al., *Treatment of vaginal bleeding irregularities induced by progestin only contraceptives.* Cochrane Database of Systematic Reviews, 2013(10): p. N.PAG-N.PAG.
5. Abdel-Aleem, H., et al., *Treatment of vaginal bleeding irregularities induced by progestin only contraceptives.* Cochrane Database of Systematic Reviews, 2007(4): p. CD003449.
6. Abdel-Aleem, H., et al., *Doxycycline in the treatment of bleeding with DMPA: A double-blinded randomized controlled trial.* Contraception, 2012. **86**(3): p. 224-230.
7. Abdel-Aleem, H., et al., *Tamoxifen treatment of bleeding irregularities associated with Norplant use.* Contraception, 2005. **72**(6): p. 432-437.
8. Abiyo, J., et al., *"I have come to remove it because of heavy bleeding": a mixed-methods study on early contraceptive implant removal and the underlying factors in eastern Uganda.* Contraception and reproductive medicine, 2024. **9**(1): p. 17.
9. Adaji, S.E., S.O. Shittu, and S.T. Sule, *Attitude of Nigerian women to abnormal menstrual bleeding from injectable progestogen-only contraceptive.* Annals of African Medicine, 2005. **4**(4): p. 144-149.
10. Adderley, U., *Do nonsteroidal anti-inflammatory drugs reduce menstrual pain and heavy bleeding in women using an intrauterine device?* Nursing in Practice: The Journal for Today's Primary Care Nurse, 2007(36): p. 8-8.
11. Adegbola, O. and O.K. Ogedengbe, *The acceptance rate of intrauterine contraceptive device (IUCD) amongst family planning clinic users in Lagos University Teaching Hospital (LUTH).* Nigerian quarterly journal of hospital medicine, 2008. **18**(4): p. 175-180.
12. Adeyemi, A.S. and D.A. Adekanle, *Progestogen-only injectable contraceptive: Experience of women in Osogbo, southwestern Nigeria.* Annals of African Medicine, 2012. **11**(1): p. 27-31.
13. Agarwal, K. and R. Dewan, *Evaluation of acceptability, safety, and continuation rates of centchroman as postabortion nonsteroidal contraceptive pill.* Contraception, 2023. **121**: p. 109961.
14. Agarwal, K., U. Sharma, and V. Acharya, *Microbial and cytopathological study of intrauterine contraceptive device users.* Indian journal of medical sciences, 2004. **58**(9): p. 394-399.
15. Agrawal, P., V. Kushwa, and B.K. Mangal, *Evaluation of safety profile of centchroman for contraceptive purpose.* 2016.
16. Agrawal, S., et al., *Insights from Client Experience with Injection Medroxyprogesterone Acetate (MPA) in India: Lessons from the Field.* Journal of Obstetrics and Gynecology of India, 2022. **72**(Supplement 1): p. 262-266.
17. Aisha, S. and M. Kausar, *Cu-375 [multiload]; a safe cheap and effective method for reversible long-term contraception*. 2006. p. 211-215.
18. Aisien, A.O., *Contraception with levonorgestrel subdermal implants (NorplantR) in Benin-City, Nigeria: a 12-year review.* African Journal of Reproductive Health, 2007. **11**(1): p. 90-97.
19. Aisien, A.O. and M.E. Enosolease, *Haemostatic function in Norplant (levonorgestrel) users: a 3-year prospective experience in Benin-City, Nigeria.* The Nigerian postgraduate medical journal, 2009. **16**(2): p. 126-131.
20. Aisien, A.O. and M.E. Enosolease, *Safety, efficacy and acceptability of Implanon a single rod implantable contraceptive (etonogestrel) in University of Benin Teaching Hospital.* Nigerian Journal of Clinical Practice, 2010. **13**(3): p. 331-335.
21. Aisien, A.O., M.E. Enosolease, and M.O. Shobowale, *Evaluation of haemostatic function in Nigerian Norplant acceptors after 12 months of use.* Journal of obstetrics and gynaecology : the journal of the Institute of Obstetrics and Gynaecology, 2005. **25**(4): p. 377-381.
22. Aisien, A.O., et al., *Changes in menstrual and haematological indices among norplant acceptors.* Contraception, 2000. **61**(4): p. 283-286.
23. Aisien, A.O., et al., *Evaluation of menstrual and haematological parameters, after 36 months of NorplantR contraception.* Journal of Obstetrics and Gynaecology, 2002. **22**(4): p. 406-410.

24. Akadri, A.A. and O.I. Odelola, *Progestogen-only injectable contraceptive: Acceptor prevalence and client experience at Sagamu, Nigeria.* The Nigerian postgraduate medical journal, 2017. **24**(3): p. 178-181.
25. Akilimali, P.Z., et al., *Incidence and determinants of Implanon discontinuation: Findings from a prospective cohort study in three health zones in Kinshasa, DRC.* PLoS One, 2020. **15**(5): p. e0232582.
26. Aktun, H., et al., *Depo-Provera: Use of a long-acting progestin injectable contraceptive in Turkish women.* Contraception, 2005. **72**(1): p. 24-27.
27. Al-Hamzawi, S.A. and S.H. Ali Al-Tameemi, *Patterns of contraceptives use and their complications in iraqi reproductive age women.* International Journal of Research in Pharmaceutical Sciences, 2020. **11**(2): p. 1411-1416.
28. Al-Humadi, F.W., et al., *Comparative assessment of the effects of two intrauterine systems for long-term contraception on some haematological, biochemical, and immunological markers.* Review of Clinical Pharmacology and Pharmacokinetics, International Edition, 2024. **38**(Supplement 2): p. 63
EP-67.
29. Alaa-Mohamed, A., et al., *Uterine artery Doppler indices: pulsatility index and resistance index as predictive tools for the incidence of heavy menstrual bleeding related to copper intrauterine contraceptive device.* Obstetrics & Gynecology Science, 2021: p. 309-316.
30. Alanwar, A., et al., *Oral micronised flavonoised versus tranexamic acid for treatment of heavy menstrual bleeding secondary to copper IUD use: a randomised doubie-blind clinical trial.* European Journal of Contraception and Reproductive Health Care, 2018. **23**(5): p. 365-370.
31. Anand, N.I., et al., *Acceptance of Injectable Contraception at Tertiary Care Hospital.* International Journal of Pharmaceutical and Clinical Research, 2024. **16**(10): p. 1031
EP-1035.
32. Ananda, Y., *Use of injectable contraceptives relation with menstrual disorders on family planning program acceptors in Lubuk Buaya Public Health Center Padang 2018.* Pemakaian alat kontrasepsi suntik berhubungan dengan gangguan menstruasi pada akseptor KB di Wilayah Kerja Puskesmas Lubuk Buaya Padang Tahun 2018., 2018. **4**(2): p. 52-56.
33. Andrade, A. and D. Wildemeersch, *Menstrual blood loss in women using the frameless FibroPlant (R) LNG-IUS.* Contraception, 2009. **79**(2): p. 134-138.
34. Andrade, A.T.L., et al., *GyneFix: um novo dispositivo intrauterino.* Bol. Centro Biol. Reprod, 2007. **26**(1/2): p. 48-45.
35. Anjos, F.C.Q.S., et al., *Clinical Assessment of 3 Intrauterine Devices in Adolescent Girls: A Randomized Clinical Trial.* Journal of Pediatric and Adolescent Gynecology, 2024. **37**(2): p. 165
EP-170.
36. Anwesha, B., et al., *Prevalence and reasons behind use of injectable contraceptive among the women of reproductive age group: a cross-sectional survey in rural areas of Nadia district, West Bengal.* JOURNAL OF FAMILY MEDICINE AND PRIMARY CARE, 2021. **10**(7): p. 2566-2571.
37. Archer, D.F., et al., *Effects of ethinyl estradiol and ibuprofen compared to placebo on endometrial bleeding, cervical mucus and the postcoital test in levonorgestrel subcutaneous implant users.* Contraception, 2008. **78**(2): p. 106-112.
38. Arowojolu, A.O., et al., *Vaginal bleeding patterns in Nigerian users of nomegestrol acetate subdermal contraceptive implant.* African journal of medicine and medical sciences, 2000. **29**(3-4): p. 275-279.
39. Arti, S., et al., *Acceptability and practice of contraception and frequency of induced abortions in married women of reproductive age group, in a tertiary-care hospital.* International Journal of Medical Science and Public Health, 2015. **4**(6): p. 845-848.
40. Austad, K., et al., *A qualitative comparison of long-and short-acting hormonal method: Users' perspectives on method selection in rural guatemala.* International Journal of Women's Health and Reproduction Sciences, 2020. **8**(4): p. 338-346.
41. Ba, M.G., J.C. Moreau, and F. Diadhiou, *Tolerance and acceptability of Norplant contraceptive devices.* Journal de Gynecologie Obstetrique et Biologie de la Reproduction, 2002. **31**(8): p. 765-771.
42. Bachmann, G. and P. Korner, *Bleeding patterns associated with non-oral hormonal contraceptives: a review of the literature.* Contraception, 2009. **79**(4): p. 247-258.
43. Bahamondes, L., et al., *Pregnancy outcomes associated with extended use of the 52-mg 20 mug/day levonorgestrel-releasing intrauterine system beyond 60 months: A chart review of 776 women in Brazil.* Contraception, 2018. **97**(3): p. 205-209.
44. Bahamondes, L., et al., *Associated factors with discontinuation use of combined oral contraceptives.* Fatores associados a descontinuacao do uso de anticoncepcionais orais combinados., 2011. **33**(6): p. 303-309.
45. Balogun, O.R., *Analysis of weight, packed cell volume changes and menstrual pattern in Norplant implant acceptors in Ilorin, Nigeria.* Nigerian Journal of Clinical Practice, 2007. **10**(2): p. 116-119.

46. Balogun, O.R., et al., *Implanon sub-dermal implant: An emerging method of contraception in Ilorin, Nigeria.* Journal of Medical and Biomedical Sciences, 2014. **3**(1): p. 1-5.
47. Balogun, O.R. and H.O. Raji, *Clinical experience with injectable progestogen- only contraceptives at University of Ilorin teaching hospital: a five year review.* The Nigerian postgraduate medical journal, 2009. **16**(4): p. 260-263.
48. Barbieri, M.M., et al., *One-year follow-up of immediate postpartum contraceptive implant insertion in adolescents.* European Journal of Contraception and Reproductive Health Care, 2023. **28**(1): p. 58-64.
49. Barreiros, F.A., et al., *Bleeding patterns of women using extended regimens of the contraceptive vaginal ring.* Contraception, 2007. **75**(3): p. 204-208.
50. Bassol, S., et al., *A 13-month multicenter clinical experience of a low-dose monophasic oral contraceptive containing 20 mug ethinylestradiol and 75 mug gestodene in Latin American women.* Contraception, 2003. **67**(5): p. 367-372.
51. Bassol, S., et al., *Mesigyna R once-a-month combined injectable contraceptive: experience in Latin America.* Contraception (Stoneham), 2000. **61**(5): p. 309-316.
52. Beesham, I., et al., *Contraceptive method preference and reasons for contraceptive discontinuation among women randomized to intramuscular depot medroxyprogesterone acetate, a copper intrauterine device or a levonorgestrel implant: Findings from Durban, South Africa.* Contraception, 2022. **108**: p. 37-43.
53. Beksinska, M.E., H.V. Rees, and J. Smit, *Temporary discontinuation: a compliance issue in injectable users.* Contraception, 2001. **64**(5): p. 309-313.
54. Belete, N., et al., *Prevalence and factors associated with modern contraceptive discontinuation among reproductive age group women, a community based cross-sectional study in Humera town, northern Ethiopia.* BMC Women's Health, 2018. **18**(1): p. 190.
55. Bertrand, J.T., et al., *An observational study to test the acceptability and feasibility of using medical and nursing students to instruct clients in DMPA-SC self-injection at the community level in Kinshasa.* Contraception, 2018. **98**(5): p. 411-417.
56. Bezabih, T., et al., *Determinants of implanon discontinuation among women in Gedeo Zone, South Ethiopia: a case-control study.* Contraception and reproductive medicine, 2024. **9**(1): p. 22.
57. Bharati, U. and V. Shrivastava, *Evaluation of Norplant in Nepalese women.* JNMA, Journal of the Nepal Medical Association, 2000. **39**(133): p. 154-157.
58. Bharia, M., et al., *COMPARISON OF SATISFACTION AND OUTCOME OF PPIUCD AND INTERVAL IUCD AT A TERTIARY CARE CENTER IN WESTERN RAJASTHAN.* Asian Journal of Pharmaceutical and Clinical Research, 2023. **16**(12): p. 249
EP-252.
59. Bhatia, P., et al., *Implanon: Subdermal single rod contraceptive implant.* Journal of Obstetrics and Gynecology of India, 2011. **61**(4): p. 422-425.
60. Boller, M., et al., *Perceptions of intra-uterine device users in mirebalais, haiti: A mixed methods study.* Annals of Global Health, 2018. **84**(4): p. 663-669.
61. Booranabunyat, S. and S. Taneepanichskul, *Implanon use in Thai women above the age of 35 years.* Contraception, 2004. **69**(6): p. 489-491.
62. Bradley, J.E., et al., *Blood, men and tears: keeping IUDs in place in Bangladesh.* Culture, Health & Sexuality, 2009. **11**(5): p. 543-558.
63. Brunie, A., et al., *Making Removals Part of Informed Choice: A Mixed-Method Study of Client Experiences With Removal of Long-Acting Reversible Contraceptives in Senegal.* Global health, science and practice, 2022. **10**(5).
64. Brunie, A., et al., *Expanding long-acting contraceptive options: a prospective cohort study of the hormonal intrauterine device, copper intrauterine device, and implants in Nigeria and Zambia.* The Lancet Global Health, 2021. **9**(10): p. e1431-e1441.
65. Buasang, K. and S. Taneepanichskul, *Efficacy of celecoxib on controlling irregular uterine bleeding secondary to jadelle use.* Journal of the Medical Association of Thailand, 2009. **92**(3): p. 301-307.
66. Burke, H.M., et al., *Effect of self-administration versus provider-administered injection of subcutaneous depot medroxyprogesterone acetate on continuation rates in Malawi: a randomised controlled trial.* The Lancet Global Health, 2018. **6**(5): p. e568-e578.
67. Burke, H.M., et al., *Young Women's Experiences With Subcutaneous Depot Medroxyprogesterone Acetate: A Secondary Analysis of a One-Year Randomized Trial in Malawi.* Journal of Adolescent Health, 2020. **67**(5): p. 700-707.
68. Burke, H.M., et al., *Acceptability of the contraceptive Sayana® Press when injected every four months: Results from a twelve-month trial in Brazil, Chile and the Dominican Republic.* Contraception, 2022. **113**: p. 95-100.

69. Callahan, R.L., et al., *Potential user interest in new long-acting contraceptives: Results from a mixed methods study in Burkina Faso and Uganda.* PLoS One, 2019. **14**(5): p. e0217333.
70. Campos, J.R. and V.H.d. Melo, *Acetado de medroxiprogesterona de depósito como anticoncepcional injetável em adolescentes.* Rev. bras. ginecol. obstet, 2001. **23**(3): p. 181-6.
71. Canto De Cetina, T.E., P. Canto, and M. Ordoez Luna, *Effect of counseling to improve compliance in Mexican women receiving depot-medroxyprogesterone acetate.* Contraception, 2001. **63**(3): p. 143-146.
72. Cartwright, A.F., et al., *Contraceptive Continuation and Experiences Obtaining Implant and IUD Removal Among Women Randomized to Use Injectable Contraception, Levonorgestrel Implant, and Copper IUD in South Africa and Zambia.* Studies in Family Planning, 2023.
73. Cartwright, A.F., et al., *Contraceptive continuation and experiences obtaining implant and IUD removal among women randomized to use injectable contraception, levonorgestrel implant, and copper IUD in South Africa and Zambia.* Studies in Family Planning, 2023. **54**(2): p. 379-401.
74. Casey, P.M., et al., *Management of etonogestrel subdermal implant-related bleeding.* Journal of reproductive medicine, 2014. **59**(3): p. 306-312.
75. Chaovisitsaree, S., et al., *One year study of Implanon on the adverse events and discontinuation.* Journal of the Medical Association of Thailand, 2005. **88**(3): p. 314-317.
76. Chappell, C.A., et al., *Contraceptive method switching among women living in sub-Saharan Africa participating in an HIV-1 prevention trial: a prospective cohort study.* Contraception, 2019. **100**(3): p. 214-218.
77. Chebet, J.J., et al., *"Every method seems to have its problems"- Perspectives on side effects of hormonal contraceptives in Morogoro Region, Tanzania.* BMC Women's Health, 2015. **15**(1): p. 97.
78. Chen, J., et al., *Clinic observation of a levonorgestrel-releasing intrauterine system inserted immediately after artificial abortion.* National Medical Journal of China, 2011. **91**(45): p. 3176-3178.
79. Chen, X., et al., *Bleeding pattern difference between levonorgestrel intrauterine system and copper intrauterine devices inserted immediately post-abortion: a multicenter, prospective, observational cohort study in Chinese women.* Current Medical Research & Opinion, 2018. **34**(5): p. 873-880.
80. Cheng, L., et al., *Once a month administration of mifepristone improves bleeding patterns in women using subdermal contraceptive implants releasing levonorgestrel.* Human Reproduction, 2000. **15**(9): p. 1969-1972.
81. Chin-Quee, D., et al., *How Much Do Side Effects Contribute to Discontinuation? A Longitudinal Study of IUD and Implant Users in Senegal.* Frontiers in global women's health, 2021. **2**: p. 804135.
82. Chotnopparatpattara, P. and S. Taneepanichskul, *Use of depot medroxyprogesterone acetate in Thai adolescents.* Contraception, 2000. **62**(3): p. 137-140.
83. Christelle, K., M.N. Norhayati, and S.H. Jaafar, *Interventions to prevent or treat heavy menstrual bleeding or pain associated with intrauterine-device use.* The Cochrane database of systematic reviews, 2022. **8**: p. CD006034.
84. Citra Dps, K., R. Andrajati, and S. Supardi, *Comparison of adverse drug reactions of second- and third-generation oral contraceptives.* Asian Journal of Pharmaceutical and Clinical Research, 2017. **10**(Special Issue October): p. 134-138.
85. Cordero Calle, M.C. and J.R. Vintimilla Maldonado, *Prevalencia y factores asociados al abandono del implante subdérmico de Etonogestrel, Azogues, Ecuador, 2014-2017.* Rev. méd. Hosp. José Carrasco Arteaga, 2020. **12**(1): p. 14-18.
86. Coutinho, E.M., et al., *Efficacy, acceptability, and clinical effects of a low-dose injectable contraceptive combination of dihydroxyprogesterone acetophenide and estradiol enanthate.* Contraception, 2000. **61**(4): p. 277-80.
87. da Silva Farias, A.G., et al., *Satisfaction of combined and exclusive injectable contraceptive users of progestogen and associated factors.* Rev Rene, 2017. **18**(3): p. 345-352.
88. Dagnaw Melesse, Y., et al., *Discontinuation of implants and associated factors among women in health facilities of Bahir Dar city, Northwest Ethiopia: A cross-sectional study.* International Journal of Africa Nursing Sciences, 2024. **20**: p. 100746.
89. Desai, J., et al., *Safety and performance of a levonorgestrel-releasing intrauterine contraceptive device: One-year outcomes of fiona-1 clinical registry.* Journal of the Indian Medical Association, 2019. **117**(8): p. 23-28.
90. Deshmukh, P., A. Deshmukh, and Y.S. Nandanwar, *Observational Study to Analyze Acceptance and Expulsion Rates of Post Placental Intra Uterine Contraceptive Device (PPIUCD) - CuT380A.* International Journal of Pharmaceutical and Clinical Research, 2024. **16**(2): p. 530
EP-537.
91. Dewan, R., et al., *Early IUD insertion after medically induced abortion.* European Journal of Contraception and Reproductive Health Care, 2018. **23**(3): p. 231-236.

92. Dikke, G.B., *Control of side effects in strategy for increasing adherence to combined oral contraceptives. The role for a three-phase desogestrel-containing drug.* Obstetrics, Gynecology and Reproduction, 2022. **16**(3): p. 244-254.
93. Dilbaz, B., et al., *The efficacy, acceptability and continuation of postpartum, post-abortive progestin-only pill: a pioneering prospective multicentric study from Turkey.* Journal of the Turkish-German Gynecological Association, 2022. **23**(4): p. 255-262.
94. Dimkpa, O.J., O.E. Okwudili, and N.E. Wamadi, *Intrauterine contraceptive device use in Port Harcourt, Southern Nigeria: a retrospective analysis.* British Journal of Medicine and Medical Research, 2014. **4**(16): p. 3132-3139.
95. Diop, S.N., et al., *Norplant contraceptive implant.* Journal de Gynecologie, Obstetrique et Biologie de la Reproduction, 2003. **32**(3 Pt 1): p. 246-251.
96. Doke, G. and J. Kamda, *A study of Centchroman users with special reference to its contraceptive benefit.* 2019. **8**.
97. Du, M.K., et al., *A 10-year follow-up study of contraceptive Norplant implants.* International Journal of Gynecology and Obstetrics, 2000. **68**(3): p. 249-256.
98. Duby, Z., et al., *"I will find the best method that will work for me": navigating contraceptive journeys amongst South African adolescent girls and young women.* Contraception and reproductive medicine, 2024. **9**(1): p. 39.
99. Ehab, A.R., et al., *Discontinuation rates among women using either the combined oral contraceptive pills or an intrauterine contraceptive device for contraception: a comparative study*. 2011. p. 27-33.
100. Ejeta Chibsa, S., et al., *Determinant of Implanon discontinuation among women in southwest Ethiopia: unmatched case control study.* Contraception and reproductive medicine, 2023. **8**(1): p. 54.
101. Ekabua, J.E. and I.H. Itam, *The safety and complications of Norplant use in Calabar.* Tropical Doctor, 2007. **37**(1): p. 37-39.
102. El-Gendy, S.D., et al., *Epidemiological study of the unmet need for contraception in Benha city.* The Journal of American Science, 2012. **8**(5): p. 125-134.
103. Elasy, A.N., A.M. Abdelghany, and A.M. Farag, *Uterine artery Doppler indices - pulsatility index and resistance index as predictive tools to menstrual changes related to levonorgestrel intrauterine system versus copper intrauterine contraceptive device among Egyptian women: a cohort study.* Italian Journal of Gynaecology and Obstetrics, 2023. **35**(4): p. 467
EP-474.
104. Enyindah, C.E. and T. Kasso, *Jadelle subdermal implants. Preliminary experience in a teaching hospital in the Niger Delta Region of Nigeria.* Nigerian journal of medicine : journal of the National Association of Resident Doctors of Nigeria, 2011. **20**(2): p. 270-274.
105. Enyindah, C.E. and F.C. Mmom, *Contraception with depot medroxy progesterone acetate (DMPA) in Port Harcourt, South-South Nigeria.* Journal of Medicine and Biomedical Research, 2015. **14**(2): p. 5-12.
106. Ezegwui, H.U., et al., *The discontinuation rate and reasons for discontinuation of implanon at the family planning clinic of University of Nigeria Teaching Hospital (UNTH) Enugu, Nigeria.* Nigerian journal of medicine : journal of the National Association of Resident Doctors of Nigeria, 2011. **20**(4): p. 448-450.
107. Ezegwui, H.U., et al., *Trend in the use of intra-uterine contraceptive device (IUCD ,TCU 380A), in Enugu, Nigeria.* Nigerian journal of medicine : journal of the National Association of Resident Doctors of Nigeria, 2013. **22**(3): p. 193-197.
108. Ezugwu, F.O. and S.E. Anya, *Five-year experience with depot medroxy progesterone acetate injectable contraception.* Nigerian journal of medicine : journal of the National Association of Resident Doctors of Nigeria, 2005. **14**(4): p. 408-410.
109. Fadiloglu, S., et al., *Relationship between copper IUD complications and ultrasonographic findings.* Archives of Gynecology & Obstetrics, 2018. **297**(4): p. 989-996.
110. Fan, G.S., et al., *Efficacy and safety of the contraceptive vaginal ring (NuvaRing) compared with a combined oral contraceptive in Chinese women: a 1-year randomised trial.* European Journal of Contraception and Reproductive Health Care, 2016. **21**(4): p. 303-309.
111. FardyAzar, Z. and A. Zanghi, *Assessment of reasons for discontinuation of different methods of contraception.* Journal of Medical Sciences (Pakistan), 2006. **6**(5): p. 823-827.
112. Fathizadeh, N., et al., *Comparing the effects of Yasmin R and LD (low-dose estrogen) as contraceptive methods on menstrual cycle changes in women referred to the health care centers of Isfahan.* Iranian journal of nursing and midwifery research, 2010. **15**(4): p. 252-8.
113. Fava, M., et al., *A randomized controlled pilot study of ulipristal acetate for abnormal bleeding among women using the 52-mg levonorgestrel intrauterine system.* International Journal of Gynecology and Obstetrics, 2020. **149**(1): p. 10-15.

114. Ferreira, J.M., et al., *Reasons for Brazilian women to switch from different contraceptives to long-acting reversible contraceptives.* Contraception, 2014. **89**(1): p. 17-21.
115. Firoozeh, V. and Z. Maryam, *Comparison of two different injectable contraceptive methods: depo-medroxy progesterone acetate [DMPA] and cyclofem*. 2013. p. 109-113.
116. Flores, J.B.O., et al., *Clinical experience and acceptability of the etonogestrel subdermal contraceptive implant.* International journal of gynaecology and obstetrics: the official organ of the International Federation of Gynaecology and Obstetrics, 2005. **90**(3): p. 228-33.
117. Garcia-Leon, F.E., et al., *Frequency of adverse effects in users with implants subskin.* Frecuencia de efectos adversos en usuarias de implante subdermico., 2011. **19**(1): p. 21-24.
118. Garg, N., et al., *Demographics and follow up of post partum intra-uterine copper device in tertiary hospital in Delhi, India.* 2019. **8**.
119. Gashaye, K.T., et al., *Reasons for modern contraceptives choice and long-acting reversible contraceptives early removal in Amhara Region, Northwest Ethiopia; qualitative approach.* BMC Women's Health, 2023. **23**(1): p. 273.
120. Gezginc, K., et al., *Contraceptive efficacy and side effects of Implanon.* The European journal of contraception & reproductive health care : the official journal of the European Society of Contraception, 2007. **12**(4): p. 362-5.
121. Gheit, S.A., *Bleeding patterns associated with progestin-only contraceptives: A prospective controlled trial comparing Mirena versus progestin-only pill.* Middle East Fertility Society Journal, 2009. **14**(3): p. 216-219.
122. Giovanelli, S.A., M.R. Torloni, and C.A.F. Guazzelli, *Post-Placental Intrauterine Device Insertion in Brazilian Adolescents: Clinical Outcomes at 12 Months.* Journal of Pediatric and Adolescent Gynecology, 2022. **35**(3): p. 336-340.
123. Godfrey, E.M., et al., *Treatment of bleeding irregularities in women with copper-containing IUDs: A systematic review.* Contraception, 2013. **87**(5): p. 549-566.
124. Godhani, H.P. and D.K. Patel, *Evaluation of post partum intrauterine contraceptive device versus interval intrauterine contraceptive device insertion.* 2019. **8**.
125. Grimes, D.A., et al., *Non-steroidal anti-inflammatory drugs for heavy bleeding or pain associated with intrauterine-device use.* Cochrane Database of Systematic Reviews, 2006: p. N.PAG-N.PAG.
126. Guazzelli, C.A.F., et al., *Manejo do sangramento inesperado em usuárias de métodos contraceptivos hormonais: revisão das recomendações atuais.* Femina, 2010. **38**(6).
127. Guazzelli, C.A.F., et al., *Extended regimens of the vaginal contraceptive ring: cycle control.* Contraception, 2009. **80**(5): p. 430-435.
128. Gunardi, E.R. and S.A. Susilo, *Menstrual Pattern and Characteristics of One-Rod and Two-Rod Levonorgestrel Implant Users.* Obstetrics & Gynecology International, 2021: p. 1-7.
129. Gupta, S., et al., *Correlates of post-partum intra-uterine copper-T devices (PPIUCD) acceptance and retention: an observational study from North India.* Contraception and reproductive medicine, 2023. **8**(1): p. 25.
130. Gupta, S., et al., *Interventional Study to Evaluate and Compare the Reasons for Contraceptive Discontinuation and Menstrual Irregularity Among the Women Using IUCD and DMPA.* Journal of Cardiovascular Disease Research, 2024. **15**(1): p. 3499
EP-3506.
131. Gupta, S., et al., *Twelve month follow-up of a contraceptive implant outreach service in rural Papua New Guinea.* The Australian & New Zealand journal of obstetrics & gynaecology, 2017. **57**(2): p. 213-218.
132. Habte, A., M. Wondimu, and H. Abdulkadir, *Survival time to Implanon discontinuation and its predictors among a cohort of Implanon users who enrolled in public hospitals of southern Ethiopia, 2021: a retrospective cohort study.* Archives of Public Health, 2022. **80**(1): p. 1-16.
133. Haddad, L.B., et al., *Contraceptive adherence among HIV-infected women in Malawi: a randomized controlled trial of the copper intrauterine device and depot medroxyprogesterone acetate.* Contraception, 2013. **88**(6): p. 737-743.
134. Hajikazemi, E., S. Nikpour, and H. Haghani, *Reasons for discontinuation of depot medroxyprogesterone acetate.* International Congress Series, 2004. **1271**(C): p. 315-318.
135. Harrington, E.K., et al., *Priorities for contraceptive method and service delivery attributes among adolescent girls and young women in Kenya: a qualitative study.* Frontiers in reproductive health, 2024: p. 01-13.
136. Hassan, E.E., E.M. Eltomy, and M. Abuzeid Khalefa, *The Impact of Contraceptive use on Women Health: A Study of Rural Area, Minia, Egypt.* Medico-Legal Update, 2021. **21**(3): p. 221-232.

137. Hassanzadeh, R., et al., *Comparison of continuation rates and reasons of discontinuation for Cyclofem and Depot-medroxyprogesterone acetate in rural areas of east Azerbaijan Province, Iran.* Journal of Family and Reproductive Health, 2012. **6**(1): p. 23-27.
138. Helmi, Z., *Potential determinants of early discontinuation of Etonogestrel implant: a cohort prospective study.* Journal of the Pakistan Medical Association, 2024. **74**(10): p. S105
EP-S110.
139. Hidalgo, M., et al., *Bleeding patterns and clinical performance of the levonorgestrel-releasing intrauterine system (Mirena) up to two years.* Contraception, 2002. **65**(2): p. 129-132.
140. HincapiÉ-GarcÍA, J.A., et al., *Causas de abandono, cambio o fallo terapéutico de la anticoncepción hormonal en mujeres universitarias.* CES med, 2013. **27**(2): p. 153-162.
141. Hlongwa, M., C. Mutambo, and K. Hlongwana, *SIGMA in fact, that's when i stopped using contraception': A qualitative study exploring women's experiences of using contraceptive methods in KwaZulu-Natal, South Africa.* BMJ Open, 2023. **13**(4): p. A342.
142. Hofmeyr, G.J., et al., *Effects of the copper intrauterine device versus injectable progestin contraception on pregnancy rates and method discontinuation among women attending termination of pregnancy services in South Africa: A pragmatic randomized controlled trial.* Reproductive health, 2016. **13**(1): p. 153.
143. Hoz, F.J.E.D.L., *One-year incidence of low libido in women using levonorgestrel subdermal implant as contraception. Cohort study. Armenia, Colombia, 2014-2019.* Revista Colombiana de Obstetricia y Ginecologia, 2021. **72**(1): p. 345-354.
144. Hu, E., L.C. Ikeako, and N.C. Obiora-Okafor, *The use of depot medroxyprogesterone acetate (DMPA) injectable contraceptive in Enugu, Nigeria.* Nigerian journal of medicine : journal of the National Association of Resident Doctors of Nigeria, 2012. **21**(3): p. 266-271.
145. Hubacher, D., et al., *Menstrual pattern changes from levonorgestrel subdermal implants and DMPA: systematic review and evidence-based comparisons.* Contraception, 2009. **80**(2): p. 113-118.
146. Hubacher, D., et al., *Preventing copper intrauterine device removals due to side effects among first-time users: Randomized trial to study the effect of prophylactic ibuprofen.* Human Reproduction, 2006. **21**(6): p. 1467-1472.
147. Huda Dr, F.A., S. Chowdhuri, and M.F.R. Sirajuddin, *Importance of appropriate counselling in reducing early discontinuation of norplant in a northern district of Bangladesh.* Journal of Health, Population and Nutrition, 2014. **32**(1): p. 142-148.
148. Hyttel, M., et al., *Use of injectable hormonal contraceptives: diverging perspectives of women and men, service providers and policymakers in Uganda.* Reproductive Health Matters, 2012. **20**(40): p. 148-157.
149. Igwe, N.M., *Intrauterine contraceptive device use in Abakaliki, southeast Nigeria: a 5-year review.* Tropical Journal of Medical Research, 2016. **19**(2): p. 138-143.
150. Imbuki, K., et al., *Factors influencing contraceptive choice and discontinuation among HIV-positive women in Kericho, Kenya.* African Journal of Reproductive Health, 2010. **14**(4 Spec no.): p. 98-109.
151. Iyengar, S., et al., *Observational study of feasibility and acceptability of the levonorgestrel-releasing intrauterine device as a long-acting reversible contraceptive in a primary care setting in India.* Contraception: X, 2022. **4**: p. 100079.
152. Izhar, R., M.A. Tahir, and S. Husain, *Femiject, a once-a-month combined injectable contraceptive: experience from Pakistan.* European Journal of Contraception and Reproductive Health Care, 2020. **25**(5): p. 359-364.
153. Jafari, A., et al., *The effect of vitamin B1 on bleeding and spotting in women using an intrauterine device: A double-blind randomised controlled trial.* European Journal of Contraception and Reproductive Health Care, 2014. **19**(3): p. 180-186.
154. Jain, A., et al., *"Side effects affected my daily activities a lot": a qualitative exploration of the impact of contraceptive side effects in Bangladesh.* OPEN ACCESS JOURNAL OF CONTRACEPTION, 2017. **8**: p. 45-52.
155. Jain, J.K., et al., *Mifepristone for the prevention of breakthrough bleeding in new starters of depo-medroxyprogesterone acetate.* Steroids, 2003. **68**(10-13): p. 1115-1119.
156. Jamali, B., et al., *Comparing the satisfaction and efficacy of cyclofem and contraceptive pills among females in Northern Iran: A randomized controlled trial study.* Journal of Advanced Pharmaceutical Technology and Research, 2014. **5**(4): p. 152-157.
157. Kakaire, O., et al., *Intrauterine Contraception Among Women Living With Human Immunodeficiency Virus: A Randomized Controlled Trial.* Obstetrics & Gynecology, 2015. **126**(5): p. 928-934.
158. Kamalifard, M., et al., *Continuation and discontinuation reasons of LD contraceptives among Iranian Women.* International Journal of Women's Health and Reproduction Sciences, 2014. **2**(5): p. 287-290.

159. Kapur, A. and S. Kumar, *Contraceptive effectiveness of levonorgestrel releasing intrauterine system.* Medical Journal Armed Forces India, 2008. **64**(2): p. 140-142.
160. Kashanian, M., F. Shahpourian, and O. Zare, *A comparison between monophasic levonorgestrel-ethinyl estradiol 150/30 and triphasic levonorgestrel-ethinyl estradiol 50-75-125/30-40-30 contraceptive pills for side effects and patient satisfaction: A study in Iran.* European Journal of Obstetrics and Gynecology and Reproductive Biology, 2010. **150**(1): p. 47-51.
161. Keogh, S.C., et al., *Hormonal contraceptive use in Ghana: The role of method attributes and side effects in method choice and continuation.* Contraception, 2021. **104**(3): p. 235-245.
162. Khan, S.A., et al., *A comparative trial of copper T 380 and Cu 375 IUCD.* Journal of Ayub Medical College, Abbottabad : JAMC, 2010. **22**(3): p. 185-187.
163. Kiriwat, O. and S. Petyim, *The study of cycle control, side effects and acceptability of transdermal patch use in Thai women in Siriraj Hospital.* Siriraj Medical Journal, 2008. **60**(6): p. 324-329.
164. Kouakou, K.P., et al., *Implant JadelleR introduction experience to Cote d'Ivoire: efficacy, incidents et side effects in 300 women.* Experience d'introduction de l'implant contraceptif JadelleR en Cote d'Ivoire: analyse de l'efficacite, des incidents et des effets indesirables chez 300 femmes., 2009. **6**(2): p. 1095-1099.
165. Laban, M., et al., *Endometrial histopathology, ovarian changes and bleeding patterns among users of long-acting progestin-only contraceptives in Egypt.* European Journal of Contraception & Reproductive Health Care, 2012. **17**(6): p. 451-457.
166. Lal, S., et al., *Efficacy of mifepristone in reducing intermenstrual vaginal bleeding in users of the levonorgestrel intrauterine system.* International Journal of Gynecology and Obstetrics, 2010. **109**(2): p. 128-130.
167. Landolt, N.K., et al., *Uptake and continuous use of copper intrauterine device in a cohort of HIV-positive women.* AIDS Care - Psychological and Socio-Medical Aspects of AIDS/HIV, 2013. **25**(6): p. 710-714.
168. Laphikanont, W. and S. Taneepanichskul, *Effects of Jadelle used in Thai women aged between 20 and 45 years in King Chulalongkorn Memorial Hospital.* Journal of the Medical Association of Thailand, 2006. **89**(6): p. 761-766.
169. Laporte, M., et al., *Reasons for satisfaction with the use of the 52-mg levonorgestrel intrauterine system.* International Journal of Gynecology and Obstetrics, 2022. **159**(2): p. 577-582.
170. Laporte, M., et al., *Effectiveness and continuation rates of the etonogestrel-subdermal contraceptive implant versus short-acting contraceptive methods offered at no cost in Campinas, Brazil.* International Journal of Gynecology and Obstetrics, 2024. **166**(1): p. 305
EP-311.
171. Lateef, S.R., M.R. Lateef, and Q.K. Abbas, *The safety and efficacy of implanon implant in a sample of Iraqi women: A cohort study.* Indian Journal of Forensic Medicine and Toxicology, 2020. **14**(1): p. 1095-1099.
172. Li, C.F.I., S.S.N. Lee, and T.C. Pun, *A pilot study on the acceptability of levonorgestrel-releasing intrauterine device by young, single, nulliparous Chinese females following surgical abortion.* Contraception, 2004. **69**(3): p. 247-250.
173. Lin, X., et al., *Preventive treatment of intrauterine device-induced menstrual blood loss with tranexamic acid in Chinese women.* Acta obstetricia et gynecologica Scandinavica, 2007. **86**(9): p. 1126-1129.
174. Liu, P., et al., *Contraception with levonorgestrel-releasing intrauterine system versus copper intrauterine device: a meta-analysis of randomized controlled trials.* EClinicalMedicine, 2024. **78**: p. 102926.
175. M.H, K., *Study of contraceptive user women in D.I.Khan, Pakistan*. 2007. p. 24-26.
176. Maceira, D., et al., *Acceptability and continuation of use of the subdermal contraceptive implant among adolescents and young women in Argentina: a retrospective cohort study.* Sexual and Reproductive Health Matters, 2023. **31**(1): p. 2189507.
177. Madden, T., et al., *Naproxen or estradiol for bleeding and spotting with the levonorgestrel intrauterine system: A randomized controlled trial.* American journal of obstetrics and gynecology, 2012. **206**(2): p. e1-129.
178. Mahboob e, A., S. Hossain, and H. Searing, *Acceptability of Sino-implant (II) in Bangladesh: final report on a prospective study.* The RESPOND Project Study Series: Contributions to Global Knowledge, 2012(8): p. xiii-pp.
179. Malunond Ali A, O., et al., *Scanning electron microscopy and histopathological study of the effect of estriol on the endometrium of women using long acting injectable contraceptives with irregular uterine bleeding*. 2009. p. 35-46.

180. Mani, P., et al., *Comparative evaluation of PPIUCD insertion in post placental vs within 48 hours of delivery.* Indian Journal of Public Health Research and Development, 2018. **9**(5): p. 21-26.
181. Manzouri, L., Z. Farajzadegan, and A.R. Zamani, *Continuation rates and reasons for discontinuing Tcu380A IUD use in Isfahan, Iran.* Journal of Family and Reproductive Health, 2011. **5**(1): p. 25-29.
182. Massai, M.R., et al., *Effect of intermittent treatment with mifepristone on bleeding patterns in Norplant implant users.* Contraception, 2004. **70**(1): p. 47-54.
183. Mercorio, F., et al., *Effectiveness and mechanism of action of desmopressin in the treatment of copper intrauterine device-related menorrhagia: A pilot study.* Human Reproduction, 2003. **18**(11): p. 2319-2322.
184. Mgobhozi, L.N., G.G. McHunu, and P. Mbeje, *Women's perceptions with use of Implanon contraceptive device at a primary healthcare facility in KwaZulu-Natal.* Health SA Gesondheid, 2023. **28**: p. a2016.
185. Mirzaee, N. and A.I. Marakhova, *THE REASONS OF DISCONTINUATION OF TCU380A IUD AND COMPARE IT WITH THE NANO-CU IUD.* Farmatsiya (Moscow), 2018. **67**(3): p. 52-56.
186. Mishra, N., S.K. Behera, and R.P. Bag, *Study of Acceptability of Postpartum Intrauterine Contraceptive Device (PPIUCD) in a Tertiary Care Hospital; Unmasking the Hole and Corner.* International Journal of Pharmaceutical and Clinical Research, 2022. **14**(6): p. 86-94.
187. Moamar, A.J., et al., *Jordanian women's experience with etonogestrel subdermal contraceptive implant in two family planning clinics*. 2015. p. 27-35.
188. Moeti, D.M.P., I. Govender, and T. Bongongo, *Early removal of etonogestrel subcutaneous contraceptive implant at a community health centre in Pretoria.* South African Family Practice, 2022. **64**(1 Part 3): p. 1-6.
189. Mohamed, A., E.S. Amal, and A.A. Samah, *Levonorgestrel releasing IUS [metraplant E] in the management of copper IUD related heavy painful menstrual loss*. 2018. p. 3158-3165.
190. Mohamed, E., H. Ahmed, and F. Ragaa, *Comparison between two types of copper bearing intrauterine device cu375 and cu380ag regarding bleeding pattern: [randomized controlled trial]*. 2018. p. 4938-4945.
191. Mohammad-Alizadeh-Charandabi, S., et al., *The effect of multivitamin supplements on continuation rate and side effects of combined oral contraceptives: A randomised controlled trial.* European Journal of Contraception and Reproductive Health Care, 2015. **20**(5): p. 361-371.
192. Mohebbi-Kian, E., S. Mohammad-Alizadeh-Charandabi, and R. Bekhradi, *Efficacy of fennel and combined oral contraceptive on depot medroxyprogesterone acetate-induced amenorrhea: A randomized placebo-controlled trial.* Contraception, 2014. **90**(4): p. 440-446.
193. Mrwebi, K.P., et al., *Reasons for Discontinuation of Implanon among Users in Buffalo City Metropolitan Municipality, South Africa: A Cross-Sectional Study.* African Journal of Reproductive Health, 2018. **22**(1): p. 113-119.
194. Mukanga, B., et al., *Perspectives on the side effects of hormonal contraceptives among women of reproductive age in Kitwe district of Zambia: a qualitative explorative study.* BMC Women's Health, 2023. **23**(1): p. 436.
195. Mukka, S. and M. Y, *Immediate post placental insertion of intrauterine contraceptive device at caesarean delivery: a prospective study.* 2020. **9**.
196. Mutihir, J.T. and D.D. Nyango, *Indications for removal of etonogestrel implant within two years of use in Jos, Nigeria.* East African Medical Journal, 2010. **87**(11): p. 461-464.
197. Nageso, A. and A. Gebretsadik, *Discontinuation rate of Implanon and its associated factors among women who ever used Implanon in Dale District, Southern Ethiopia.* BMC Women's Health, 2018. **18**(1): p. 189.
198. Nahidi, F. and S. Jalalinia, *Comparing the complications of 2 copper intrauterine devices: T380A and Cu-Safe 300.* Eastern Mediterranean Health Journal, 2008. **14**(1): p. 95-102.
199. Nakalema, S., et al., *Pharmacokinetics of levonorgestrel and etonogestrel contraceptive implants over 48 weeks with rilpivirine- or darunavir-based antiretroviral therapy.* Journal of Antimicrobial Chemotherapy, 2022. **77**(11): p. 3144-3152.
200. Nakhaee, N. and A.R. Mirahmadizadeh, *Five-year continuation rate and reasons for early removal of Norplant in Shiraz, Iran.* European Journal of Contraception and Reproductive Health Care, 2002. **7**(4): p. 223-226.
201. Nalini, N., et al., *Acceptance, safety and efficacy of postpartum intrauterine contraceptive device.* JOURNAL OF FAMILY MEDICINE AND PRIMARY CARE, 2023. **12**(5): p. 868-873.
202. Nanda, G., et al., *Experiences with the levonorgestrel-releasing intrauterine system in Kenya: qualitative interviews with users and their partners.* European Journal of Contraception and Reproductive Health Care, 2018. **23**(4): p. 303-308.
203. Naser O, M., A.R. Ehab S, and G. Ahmed S, *[Why] do depo-provera users discontinue?* 2009. p. 70-74.

204. Nathirojanakun, P., S. Taneepanichskul, and N. Sappakitkumjorn, *Efficacy of a selective COX-2 inhibitor for controlling irregular uterine bleeding in DMPA users.* Contraception, 2006. **73**(6): p. 584-587.
205. Neinavaei, M., et al., *Comparison the effects of naproxen, indomethacin and ibuprofen in treatment of spotting due to IUD insertion.* Iranian Journal of Obstetrics, Gynecology and Infertility, 2014. **17**(110): p. 8-15.
206. Ninama, S.N., T.A. Shah, and M.R. Gandhi, *Prospective study on outcome of post-partum intrauterine contraceptive device insertion at tertiary level rural health institute of Gujarat, India.* 2019. **8**.
207. Ntimani, J.M. and M.B. Randa, *Experiences of women on the use of Implanon NXT in Gauteng province, South Africa: A qualitative study.* Health SA Gesondheid, 2024. **29**: p. a2237.
208. Obsa, M.S., et al., *Lived experience of women who underwent early removal of long-acting family planning methods in bedesa town, wolaita zone, southern ethiopia: A phenomenological study.* International journal of women's health, 2021. **13**: p. 645-652.
209. Odwe, G., et al., *Which contraceptive side effects matter most? Evidence from current and past users of injectables and implants in Western Kenya.* Contraception: X, 2020. **2**: p. 100030.
210. Ohazurike, E., et al., *Predictors of Discontinuation of Subdermal Levonorgestrel Implants (Jadelle) at the Lagos University Teaching Hospital, Lagos, Nigeria: An Analytic Cohort Study.* African Journal of Reproductive Health, 2020. **24**(2): p. 48-63.
211. Ojule, J.D. and E.O. Oranu, *Clinical experience with progestogen only injectable contraceptive in a tertiary institution in southern Nigeria: a ten year review.* East African Medical Journal, 2017. **94**(3): p. 201-206.
212. Ojule, J.D., V.K. Oriji, and C. Okongwu, *A five year review of the complications of progestogen only injectable contraceptive at the University of Port-Harcourt Teaching Hospital.* Nigerian journal of medicine : journal of the National Association of Resident Doctors of Nigeria, 2010. **19**(1): p. 87-95.
213. Okunlola, M.A., et al., *Biosocial Profiles and Pattern of Complaints of New Intrauterine Device Acceptors at the University College Hospital, Ibadan, Nigeria.* Journal of Reproduction and Contraception, 2009. **20**(2): p. 93-100.
214. Olaifa, B.T., et al., *Reasons given by women for discontinuing the use of progestogen implants at Koster Hospital, North West province.* South African Family Practice, 2022. **64**(1): p. a5471.
215. Oliveira, E.C.F.D., et al., *Use of 52-mg Levonorgestrel-Releasing Intrauterine System in Adolescents and Young Adult Women: 3-Year Follow-Up.* Journal of Pediatric and Adolescent Gynecology, 2023. **36**(1): p. 45-50.
216. Oliveira, E.C.F.D. and A.L.L. Rocha, *Five-year Contraceptive Use of 52-mg Levonorgestrel Releasing Intrauterine System in Young Women, Menstrual Patterns, and New Contraceptive Choice.* Revista Brasileira de Ginecologia e Obstetricia, 2023. **45**(11): p. E654
EP-E660.
217. Onyango, G.O., et al., *Perceptions of contraceptives as factors in birth outcomes and menstruation patterns in a rural community in Siaya county, Western Kenya.* Journal of Global Health Reports, 2020. **4**.
218. Oshodi, Y.A., et al., *Weight gain and menstrual abnormalities between users of Depo-provera and Noristerat.* 2019. **8**.
219. Pam, V.C., et al., *Sociodemographic profiles and use-dynamics of Jadelle (levonorgestrel) implants in Jos, Nigeria.* Nigerian medical journal : journal of the Nigeria Medical Association, 2016. **57**(6): p. 314-319.
220. Pandit, S.N., et al., *Multicenter Study of Contraceptive Vaginal Ring (NuvaRing(R)) in Normal Daily Practice in Indian Women.* Journal of obstetrics and gynaecology of India, 2014. **64**(6): p. 409-16.
221. Patiño V, A., et al., *Evolución a un año de los efectos adversos, en una cohorte de pacientes con implante subdérmico de Desogestrel.* Rev. chil. obstet. ginecol, 2006. **71**(3): p. 170-173.
222. Pérez Parra, Z., et al., *Caracterización del uso del implante subdérmico norplant.* Rev. cuba. med. gen. integr, 2001. **17**(4): p. 344-348.
223. Phaliwong, P. and S. Taneepanichskul, *The effect of mefenamic acid on controlling irregular uterine bleeding second to Implanon (R) use.* Journal of the Medical Association of Thailand, 2004. **87**(Suppl. 3): p. S64-S68.
224. Phupong, V., A. Sophonsritsuk, and S. Taneepanichskul, *The effect of tranexamic acid for treatment of irregular uterine bleeding secondary to Norplant use.* Contraception, 2006. **73**(3): p. 253-256.
225. Pillay, D., et al., *User perspectives on Implanon NXT in South Africa: A survey of 12 public-sector facilities.* South African medical journal = Suid-Afrikaanse tydskrif vir geneeskunde, 2017. **107**(10): p. 815-821.
226. Pizzi, R., et al., *Contraceptive vaginal ring (MyRing): multicenter study in Venezuelan women.* Revista de Obstetricia y Ginecologia de Venezuela, 2023. **83**(2): p. 142

EP-151.
227. Polis, C.B., et al., *Preference for Sayana Press versus intramuscular Depo-Provera among HIV-positive women in Rakai, Uganda: A randomized crossover trial.* Contraception, 2014. **89**(5): p. 385-395.
228. Power, J., R. French, and F. Cowan, *Subdermal implantable contraceptives versus other forms of reversible contraceptives or other implants as effective methods of preventing pregnancy.* Cochrane Database of Systematic Reviews, 2007(3).
229. Pradhan, S., et al., *Intrauterine contraceptive discontinuation reasons among female trial participants living with HIV in Cape Town, South Africa: A qualitative analysis.* Frontiers in global women's health, 2023. **4**: p. 1010794.
230. Puri, M.C., et al., *Exploring reasons for discontinuing use of immediate post-partum intrauterine device in Nepal: a qualitative study.* Reproductive health, 2020. **17**(1): p. 1-6.
231. Qin, L., J.M. Goldberg, and G. Hao, *A 4-year follow-up study of women with Norplant-2 contraceptive implants.* Contraception, 2001. **64**(5): p. 301-303.
232. Qiu, M., et al., *Contraceptive Implant Discontinuation in Huambo and Luanda, Angola: A Qualitative Exploration of Motives.* Maternal & Child Health Journal, 2017. **21**(9): p. 1763-1771.
233. Rahman, A., et al., *Contraceptive practice of married women: Experience from a rural community of Bangladesh.* Journal of Medicine (Bangladesh), 2014. **15**(1): p. 9-13.
234. Rai, L., P. Prabakar, and S. Nair, *Injectable depot medroxyprogesterone - A safe and an effective contraception for an indian setting.* Health and Population: Perspectives and Issues, 2007. **30**(1): p. 12-23.
235. Rajabi, N.M., et al., *A comparative study of the effects of cumin and mefenamic acid capsules on menstrual bleeding in iud users: A randomized triple blind clinical trial.* Journal of Medicinal Plants, 2015. **14**(54): p. 159-168.
236. Rajaraman, R., S. Vaithilingan, and T.S. Selvavinayagam, *Acceptance, Adherence, and Side Effects of Depot Medroxyprogesterone Acetate: A Prospective Observational Study.* CUREUS, 2024. **16**(4): p. e58700.
237. Ramos, G. and R. Segovia, *Experiencia con el implante subdérmico en adolescentes en la Catedra y Servicio de Ginecología y Obstetricia.* Med. clín. soc, 2021. **5**(2).
238. Rasheed, S.M. and A.M. Abdelmonem, *Complications among adolescents using copper intrauterine contraceptive devices.* International Journal of Gynecology and Obstetrics, 2011. **115**(3): p. 269-272.
239. Ravolamanana, L.R., P.G. Randaoharison, and D.L. Razafintsalama, *Subcutaneous levonorgestrel (Norplant) implants for contraception at the CHU Mahajanga, Madagascar: preliminary results.* La contraception par les implants sous-cutanes de levonorgestrel (NorplantR) au CHU de Mahajanga, Madagascar: resultats preliminaires., 2000. **47**(10): p. 410-415.
240. Ray, S., et al., *Experiences of "Antara": The Injectable Contraceptive in Rural Indian Women Presenting to a Tertiary Care Hospital of Eastern India.* Journal of Obstetrics and Gynecology of India, 2024. **74**(3): p. 243
EP-249.
241. Rehan, N., A. Inayatullah, and I. Chaudhary, *Norplant: Reasons for discontinuation and side-effects.* European Journal of Contraception and Reproductive Health Care, 2000. **5**(2): p. 113-118.
242. Roberts, A.O., et al., *Profile of Implanon acceptors and pattern of side effects.* Journal of Reproduction and Contraception, 2015. **26**(1): p. 46-52.
243. Rocca, M.L., et al., *Safety and benefits of contraceptives implants: A systematic review.* Pharmaceuticals, 2021. **14**(6): p. 548.
244. Rothschild, C.W., et al., *Contributions of side effects to contraceptive discontinuation and method switch among Kenyan women: a prospective cohort study.* BJOG: An International Journal of Obstetrics and Gynaecology, 2022. **129**(6): p. 926-937.
245. Roy, M., et al., *Progesterone vaginal ring as a new contraceptive option for lactating mothers: Evidence from a multicenter non-randomized comparative clinical trial in India.* Contraception, 2020. **102**(3): p. 159-167.
246. Ruminjo, J.K., et al., *Comparative acceptability of combined and progestin-only injectable contraceptives in Kenya.* Contraception, 2005. **72**(2): p. 138-145.
247. Rwebazibwa, J., et al., *Early contraceptive implant removal and associated factors among women attending public family planning clinics, Mbarara City, Southwestern Uganda: a cross-sectional study.* Contraception and reproductive medicine, 2024. **9**(1): p. 38.
248. Sadeghi-Bazargani, H., et al., *Low-dose oral contraceptive to re-induce menstrual bleeding in amenorrheic women on DMPA treatment: A randomized clinical trial.* Medical Science Monitor, 2006. **12**(10): p. CR420-CR425.

249. Sadeghi-Bazargani, H. and Z. Fardyazar, *Amenorrhea: An advantage rather than a complication of depot medroxy progesterone acetate injectable contraceptive.* International Journal of Pharmacology, 2006. **2**(3): p. 352-356.
250. Saharkhiz, N., et al., *A comparative trial of the efficacy of mefenamic acid and tranexamic acid for treatment of menorrhagia induced by copper T-380A IUD.* International Journal of Women's Health and Reproduction Sciences, 2017. **5**(3): p. 175-180.
251. Saini, V.K., et al., *An Assessment of Knowledge and Attitude of Beneficiaries Attending Tertiary Care Hospital regarding Temporary Family Planning Methods and to Determine their Experienced Side Effects of Temporary Contraceptive Methods in Ahmedabad City, Gujarat, India.* Journal of the Indian Medical Association, 2024. **122**(8): p. 24 EP-28.
252. Santoso, B., et al., *The Effectiveness, Changes in Body Weight, and Menstrual Cycle Between 2 Rods and 1 Rod Contraceptive Implants after 12 Months of Insertion.* Current Women's Health Reviews, 2024. **20**(2): p. 113 EP-119.
253. Sari, R.J., R. Susanti, and Sudarianti, *Determinants of Weight Increase and Menstrual Cycle Irregularities in KB Implant Acceptors in Pematang Cengal, Tanjung Pura District.* International Journal of Public Health Excellence, 2023. **3**(1): p. 1-10.
254. Savabi-Esfahany, M., S. Fadaei, and A. Yousefy, *Use of combined oral contraceptives: Retrospective study in Isfahan, Islamic Republic of Iran.* Eastern Mediterranean Health Journal, 2006. **12**(3-4): p. 417-422.
255. Say, L., N. Ortayli, and H. Nalbant, *Women's acceptance of an injectable progestin-only contraceptive in a free-choice environment in Turkey.* European Journal of Contraception and Reproductive Health Care, 2000. **5**(1): p. 68-70.
256. Schrumpf, L.A., et al., *Side effect concerns and their impact on women's uptake of modern family planning methods in rural Ghana: a mixed methods study.* BMC Women's Health, 2020. **20**(1): p. 1-8.
257. Schwarz, J., et al., *"So that's why I'm scared of these methods": Locating contraceptive side effects in embodied life circumstances in Burundi and eastern Democratic Republic of the Congo.* Social Science and Medicine, 2019. **220**: p. 264-272.
258. Sekler, E. and F. Limongi, *Acetato de medroxiprogesterona de depósito como anticonceptivo en la adolescente.* Rev. obstet. ginecol. Venezuela, 2001. **61**(1): p. 43-47.
259. Senthong, A.J. and S. Taneepanichskul, *The effect of tranexamic acid for treatment irregular uterine bleeding secondary to DMPA use.* Journal of the Medical Association of Thailand, 2009. **92**(4): p. 461-465.
260. Shafaie, F.S., et al., *Compare of continuation rate and reasons for discontinuation of DMPA contraceptive among Iranian women referred to Tabriz and Ardebil health centers.* International Journal of Women's Health and Reproduction Sciences, 2014. **2**(4): p. 240-244.
261. Shakya Shrestha, S., et al., *Hormonal contraceptives use and their adverse effects: A cross-sectional study among the women visiting tertiary care center.* Kathmandu University Medical Journal, 2020. **18**(71): p. 296-302.
262. Shariati, M., et al., *Factors affecting Discontinuation of the Once-a-month Injectable Contraceptive (Cyclofem) in Neyshabur, Iran.* Journal of Midwifery & Reproductive Health, 2018. **6**(2): p. 1244-1252.
263. Sharma, A., et al., *prospective study of immediate postpartum intra uterine device insertion in a tertiary level hospital.* 2015.
264. Sherpa, L.Y., et al., *A prospective cohort study to assess the acceptability of Sayana Press among 18-49-year-old women in Nepal.* Contraception, 2021. **104**(6): p. 623-627.
265. Shrivastava, D. and P. Kumari, *Analysis of Long-acting Injectable Contraceptive (DMPA), Demographic, Acceptance and Future Prospects-A Seven-Year Study.* SSR Institute of International Journal of Life Sciences, 2024. **10**(5): p. 6196 EP-6202.
266. Shruti, G. and G. Shailesh, *Study of efficacy and complications on follow up of post-partum IUCD (PPIUCD) in a tertiary care hospital of Delhi.* National Journal of Medical and Allied Sciences, 2019. **8**(1): p. 12-15.
267. Singal, S., et al., *Clinical outcome of postplacental copper T 380A insertion in women delivering by caesarean section.* Journal of Clinical and Diagnostic Research, 2014. **8**(9): p. OC01-OC04.
268. Sivin, I., et al., *Two-year performance of a Nestorone-releasing contraceptive implant: A three-center study of 300 women.* Contraception, 2004. **69**(2): p. 137-144.
269. Sona Dayan, K., N. Koshy, and K.V. Jaya, *CONTRACEPTIVE PRACTICES AND UNMET NEED FOR FAMILY PLANNING IN CURRENTLY MARRIED WOMEN OF 15-49 YEARS OF AGE IN A RURAL AREA OF THRISSUR DISTRICT.* International Journal of Medicine and Public Health, 2024. **14**(4): p. 428 EP-436.

270. Soni, A., S. Garg, and R. Bangar, *Efficacy, user acceptability, tolerability, and cycle control of a combined contraceptive vaginal ring: The indian perspective.* Journal of Obstetrics and Gynecology of India, 2013. **63**(5): p. 337-341.
271. Sordal, T., et al., *Management of initial bleeding or spotting after levonorgestrel-releasing intrauterine system placement: A Randomized Controlled Trial.* Obstetrics and gynecology, 2013. **121**(5): p. 934-941.
272. Sothornwit, J., Y. Werawatakul, and O. Saenbon, *Mefenamic acid for the prevention of bleeding and spotting from depot-medroxyprogesterone acetate: A randomized controlled trial.* International Journal of Women's Health and Reproduction Sciences, 2021. **9**(4): p. 291-294.
273. Stanback, J., A.K. Mbonye, and M. Bekiita, *Contraceptive injections by community health workers in Uganda: A nonrandomized community trial.* Bulletin of the World Health Organization, 2007. **85**(10): p. 768-773.
274. Steiner, M.J., et al., *Sino-implant (II) - a levonorgestrel-releasing two-rod implant: systematic review of the randomized controlled trials.* Contraception, 2010. **81**(3): p. 197-201.
275. Suárez Espinosa, I. and R. Valdés Carrillo, *Perfil lipídico y alteraciones menstruales en mujeres que usan el anticonceptivo inyectable combinado mesigyna.* Rev. cienc. med. Pinar Rio, 2004. **8**(1): p. 53-62.
276. Subrat, R. and V.B. Sovani, *A postmarketing observational study assessing acceptability and reliability of desogestrel only contrapceptive pill (CerazetteR) in Indian women.* Journal of the Indian Medical Association, 2006. **104**(11): p. 653-656.
277. Sudev, R.O.Y., S. Patra, and S. Chalkrabarty, *A STUDY ON ACCEPTANCE AND COMPLIANCE OF DEPOT MEDROXY PROGESTERONE ACETATE FOR POST-PARTUM CONTRACEPTION IN A TERTIARY CARE CENTRE IN EASTERN INDIA.* Asian Journal of Pharmaceutical and Clinical Research, 2024. **17**(1): p. 61 EP-63.
278. Suleiman, B.U., et al., *The use of levonorgestrel releasing intrauterine system at Ahmadu Bello University Teaching Hospital, Zaria.* Nigerian Journal of Basic and Clinical Sciences, 2017. **14**(1): p. 30-33.
279. Sultana, J., et al., *Long Term Evaluation of Post-Partum Intrauterine Device (PPIUD) In Terms of Safety and Efficacy.* Bangladesh Journal of Obstetrics and Gynecology, 2022. **35**(2): p. 81-89.
280. Sun, D.L., Q.X. Shao, and G.W. Sang, *A multicentered clinical trial of the long-acting injectable contraceptive Depo Provera in Chinese women.* Contraception, 2000. **62**(1): p. 15-18.
281. Suri, V., et al., *Safety of intrauterine contraceptive device (copper T 200 B) in women with cardiac disease.* Contraception, 2008. **78**(4): p. 315-318.
282. Suthipongse, W. and S. Taneepanichskul, *An open-label randomized comparative study of oral contraceptives between medications containing 3 mg drospirenone/30 mug ethinylestradiol and 150 mug levonogestrel/30 mug ethinylestradiol in Thai women.* Contraception, 2004. **69**(1): p. 23-26.
283. Suwanmalee, O. and S. Taneepanichskul, *A clinical study of transdermal contraceptive patch in Thai women.* Journal of the Medical Association of Thailand, 2006. **89**(SUPPL. 4): p. S1-S4.
284. Taneepanichskul, S. and P. Intharasakda, *Efficacy and side effects of Norplant use in Thai women above the age of 35 years.* Contraception, 2001. **64**(5): p. 305-307.
285. Taneepanichskul, S., R. Kriengsinyot, and U. Jaisamrarn, *A comparison of cycle control, efficacy, and side effects among healthy Thai women between two low-dose oral contraceptives containing 20 mu g ethinylestradio1/75 mu g gestodene (Meliane) and 30 mu g ethinylestradio1/75 mu g gestodene (Gynera((R))).* Contraception, 2002. **66**(6): p. 407-409.
286. Taneepanichskul, S., D. Reinprayoon, and S. Phaosavadi, *DMPA use above the age of 35 in Thai women.* Contraception, 2000. **61**(4): p. 281-282.
287. Taneepanichskul, S. and C. Tanprasertkul, *Use of Norplant implants in the immediate postpartum period among asymptomatic HIV-1-positive mothers.* Contraception, 2001. **64**(1): p. 39-41.
288. Tang, G.H., et al., *Efficacy and side effects of immediate postcoital levonorgestrel used repeatedly for contraception.* Contraception, 2000. **61**(5): p. 303-308.
289. Tang, H.y., et al., *Post-marketing Surveillance for Combined Oral Contraceptive Containing Desogestrel (Marvelon®) in Chinese Rural Areas.* Journal of Reproduction and Contraception, 2008. **19**(4): p. 193-200.
290. Tantiwattanakul, P. and S. Taneepanichskul, *Effect of mefenamic acid on controlling irregular uterine bleeding in DMPA users.* Contraception, 2004. **70**(4): p. 277-279.
291. Tesfaye, H., E. Negara, and K. Bayisa, *Early implanon discontinuation and associated factors among women ever used implanon in Mettu district, Oromia regional state, southwest Ethiopia, 2021.* Reproductive health, 2021. **18**(1): p. 1-7.

292. Thamkhantho, M., et al., *One-year assessment of women receiving sub-dermal contraceptive implant at Siriraj Family Planning Clinic.* Journal of the Medical Association of Thailand, 2008. **91**(6): p. 775-880.
293. Thamkhantho, M. and N. Wetphitthayakhom, *A randomized controlled trial of clinical usage between desogestrel and lynestrenol for oral contraception in postpartum women.* Journal of the Medical Association of Thailand, 2017. **100**(12): p. 1249-1254.
294. Thapa, S., *Early discontinuation of intrauterine device in Nepal – a retrospective study.* 2012.
295. Tiras, M.B., et al., *Effects of a monthly injectable steroidal contraceptive, Mesigyna, on menstrual pattern, lipoproteins, and coagulation parameters.* Contraception, 2001. **63**(3): p. 151-153.
296. Todd, C.S., et al., *Influence of culture on contraceptive utilization among HIV-positive women in Brazil, Kenya, and South Africa. [References]*. 2011: AIDS and Behavior. Vol.15(2), 2011, pp. 454-468.
297. Tolley, E., et al., *The impact of menstrual side effects on contraceptive discontinuation: findings from a longitudinal study in Cairo, Egypt.* International Family Planning Perspectives, 2005. **31**(1): p. 15-23.
298. Umukoro, E.K., et al., *Use and Effects of Contraceptives among Female Secondary School Students in Abraka Community, Delta State, Nigeria.* Journal of Applied Sciences & Environmental Management, 2020. **24**(1): p. 153-156.
299. Upawi, S.N., et al., *Management of bleeding irregularities among etonogestrel implant users: Is combined oral contraceptives pills or nonsteroidal anti-inflammatory drugs the better option?* Journal of Obstetrics and Gynaecology Research, 2020. **46**(3): p. 479-484.
300. Urmila, K., S. Mehrunissa, and S. Shaheen, *Effectiveness and complications of multi load [MLU 375] intrauterine device among females attending family planning clinic*. 2004. p. 64-68.
301. van der Heijden, P.A.H.H., et al., *What is the best drug treatment for premenopausal women with bleeding irregularities using the levonorgestrel-releasing intrauterine system? A systematic review.* European Journal of Contraception and Reproductive Health Care, 2020. **25**(6): p. 484-491.
302. Vieira, C.S., et al., *Timing of postpartum etonogestrel-releasing implant insertion and bleeding patterns, weight change, 12-month continuation and satisfaction rates: a randomized controlled trial.* Contraception, 2019. **100**(4): p. 258-263.
303. von Kesseru, E., et al., *Premenopause contraception with monthly injectable Mesigyna with special emphasis on serum lipid and bone density patterns.* Contraception, 2000. **61**(5): p. 317-22.
304. Wanyonyi, S.Z., W.R. Stones, and E. Sequeira, *Health-related quality of life changes among users of depot medroxyprogesterone acetate for contraception.* Contraception, 2011. **84**(5): p. e17-22.
305. Wasim, T., et al., *Outcome of immediate postpartum insertion of intrauterine contraceptive device: Experience at tertiary care hospital.* Journal of the Pakistan Medical Association, 2018. **68**(4): p. 519-525.
306. Weisberg, E., et al., *A randomized controlled trial of treatment options for troublesome uterine bleeding in Implanon users.* Human Reproduction, 2009. **24**(8): p. 1852-1861.
307. Weldekidan, H.A., et al., *Discontinuation rate of long-acting reversible contraceptives and associated factors among reproductive-age women in Butajira town, Central Ethiopia.* Women's Health, 2022. **18**.
308. Wen, J., et al., *Comparative safety and effectiveness of TCu380A versus MLCu375: A systematic review of randomized trials.* Journal of Evidence-Based Medicine, 2009. **2**(4): p. 226-241.
309. Wittmann, B.Z., et al., *Consecutive use of the 52 mg levonorgestrel-releasing intrauterine system: variations in bleeding patterns.* Revista Brasileira de Ginecologia e Obstetricia, 2020. **42**(4): p. 194-199.
310. Wu, S., et al., *Copper T380A intrauterine device for emergency contraception: A prospective, multicentre, cohort clinical trial.* BJOG: An International Journal of Obstetrics and Gynaecology, 2010. **117**(10): p. 1205-1210.
311. Xess, S., et al., *QUALITY IMPROVEMENT INITIATIVE TO INCREASE THE ACCEPTANCE RATE OF TABLET CHHAYA (CENTCHROMAN) IN THE IMMEDIATE POSTPARTUM PERIOD IN THE TERTIARY CARE CENTRE OF RSDKS GMC AMBIKAPUR.* International Journal of Academic Medicine and Pharmacy, 2024. **6**(5): p. 273 EP-276.
312. Yadav, M., A. Bharti, and G. Kour, *Evaluation of Expulsion and Continuation Rate of Immediate Postpartum Intrauterine Contraceptive Devices: A Prospective Hospital-based Study.* Journal of Clinical and Diagnostic Research, 2022. **16**(12): p. QC33-QC36.
313. Yadav, P., et al., *PPIUCD as a choice of long acting contraceptive device: A seven year experience in Indian population.* JK Practitioner, 2020. **25**(1-4): p. 39-43.
314. Yazdanpanah, M., et al., *Acceptability and side effects of Cyclofem© once-a-month injectable contraceptive in Kerman, Iran.* Iranian Journal of Reproductive Medicine, 2010. **8**(4): p. 191-196.
315. Yehuala, T., et al., *Determinants of Implanon Discontinuation among Women Who Use Implanon at Bahir Dar Town Health Institutions, Northwest Ethiopia, 2019: A Case-Control Study.* Evidence-based Complementary and Alternative Medicine, 2020. **2020**: p. 9048609.

316. Yildizbas, B., et al., *Side effects and acceptability of Implanon: A pilot study conducted in eastern Turkey.* European Journal of Contraception and Reproductive Health Care, 2007. **12**(3): p. 248-252.
317. Yu, J., et al., *Comparative study on contraceptive efficacy and clinical performance of the copper/low-density polyethylene nanocomposite IUD and the copper T220C IUD.* Contraception, 2008. **78**(4): p. 319-323.
318. Yusuf, M., M. Zakari, and B.A. Darma, *Side effects, discontinuation, and failure rates of copper T intrauterine contraceptive device in Kano, Northern Nigeria.* Nigerian Journal of Basic and Clinical Sciences, 2024. **21**(2): p. 161-164.
319. Zheng, W., et al., *Three-year observational study of immediate post-abortion insertion versus menstrual insertion of etonogestrel contraceptive implant.* BMC Women's Health, 2021. **21**(436).
320. Zimmerman, L.A., et al., *Association between experience of specific side-effects and contraceptive switching and discontinuation in Uganda: results from a longitudinal study.* Reproductive health, 2021. **18**(1): p. 1-12.
321. Zimmerman, L.A., et al., *Measuring experiences and concerns surrounding contraceptive induced side-effects in a nationally representative sample of contraceptive users: Evidence from PMA Ethiopia.* Contraception: X, 2022. **4**: p. 100074.
